# Macrocyclization of backbone *N*-methylated peptides by a prolyl oligopeptidase with a distinctive substrate recognition mechanism†

**DOI:** 10.1039/d5sc03723a

**Published:** 2025-07-02

**Authors:** Emmanuel Matabaro, Haigang Song, Lukas Sonderegger, Fabio Gherlone, Andrew M. Giltrap, Sam Liver, Alvar D. Gossert, Markus Künzler, James H. Naismith

**Affiliations:** a Institute of Microbiology, Department of Biology, ETH Zürich Vladimir-Prelog-Weg 4 CH-8093 Zürich Switzerland mkuenzle@ethz.ch; b Structural Biology, The Rosalind Franklin Institute Harwell Science & Innovation Campus Didcot OX11 0QS UK; c Division of Structural Biology, University of Oxford, The Wellcome Centre for Human Genetics Headington Oxford OX3 7BN UK james.naismith@strubi.ox.ac.uk; d Biomolecular NMR Spectroscopy Platform, Department of Biology, ETH Zürich Otto-Stern-Weg 5 CH-8093 Zürich Switzerland

## Abstract

Macrocyclization and multiple backbone *N*-methylations can significantly improve the pharmacological properties of peptides. Since chemical synthesis of such compounds is often challenging, enzyme-based production platforms are an interesting option. Here, we characterized OphP, a serine peptidase involved in the cyclization of omphalotins, a group of ribosomally produced dodecapeptides with multiple backbone *N*-methylations. OphP displays robust peptidase and macrocyclase activity towards multiply α-*N*-methylated peptides of various lengths and composition derived from the omphalotin precursor protein OphMA. In addition, OphP processes, with lower efficiency, peptides unrelated to OphMA, containing a ^Me^Gly, ^Me^Ala or Pro residue at the P1 site. Structural analysis reveals that OphP adopts a canonical prolyl oligopeptidase fold but, unlike other enzymes of this enzyme family, recognizes its substrates by their hydrophobic and multiply backbone *N*-methylated core rather than by the follower peptide. The activity of OphP could be harnessed for the enzymatic production of therapeutic peptides.

## Abstract 2

Proposed model of OphP structure and substrate recognition mechanism. OphP adopts a canonical prolyl oligopeptidase (POP) fold but its substrate recognition mechanism is distinct from other members of the POP family such as GmPOPB.

## Graphical abstract

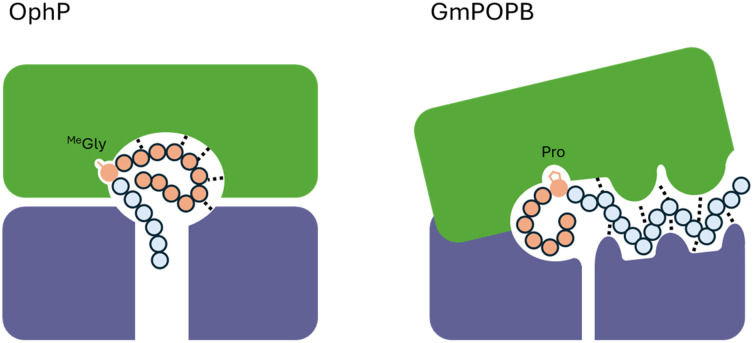

## Introduction

**Fig. 1:**
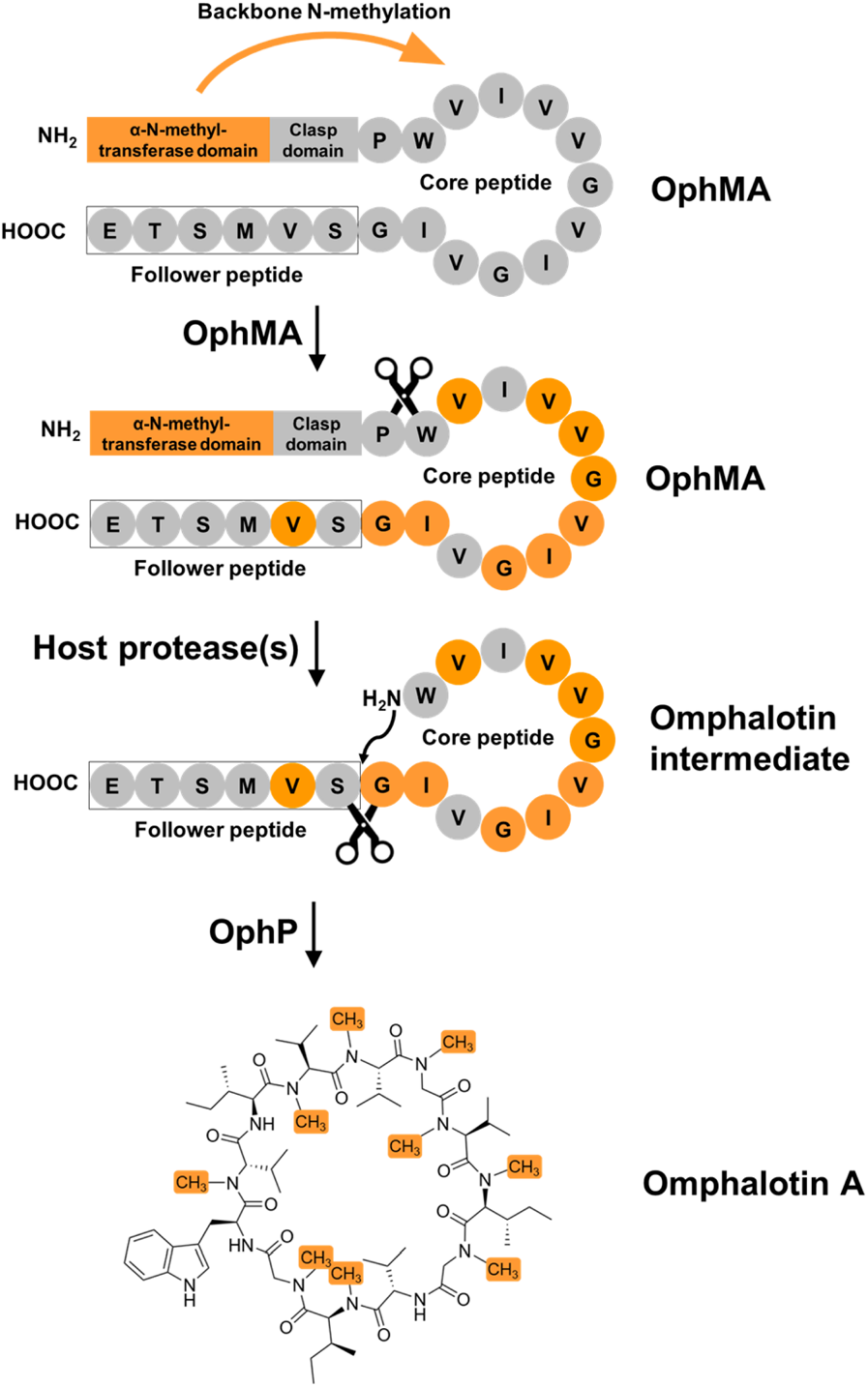
Proposed biosynthetic pathway of omphalotin A. The proposed steps include α-*N*-automethylation of the core peptide in OphMA, proteolytic cleavage of OphMA in the clasp domain (connecting the α-*N*-methyltransferase domain and the core peptide) to release the omphalotin intermediate comprising the core and follower peptide by a yet-to-be identified host protease, and macrocyclization of the core peptide by OphP. Albeit OphP was known to be required for this pathway, its exact function and selectivity remained unclear. α-*N*-Methylated residues are indicated by solid orange circles, non-methylated residues by grey circles, and the follower peptide by a box.

Backbone modifications such as macrocyclization and α-*N*-methylation are known to improve the pharmacological properties of peptide drugs, including membrane permeability, proteolytic stability and intracellular targeting.^1–7^ Interestingly, peptide natural products often display these and other modifications, most likely because they enhance their bioactivity in an ecological context.^8^ The archetypical example is cyclosporin A, a macrocyclic undecapeptide with seven α-*N*-methylations, produced by the fungus *Tolypocladium inflatum*. This peptide natural product was identified as antifungal compound and has been applied as “blockbuster” immunosuppressant since the late 1970s.^9–11^ Cyclosporin A is synthesized by a non-ribosomal peptide synthetase (NRPS), that includes an *S*-adenosyl-methionine (SAM)-dependent α-*N*-methyltransferase domain catalyzing the methylation of specific amino acid building blocks, and a C-terminal esterase domain mediating the release and macrocyclization of the assembled peptide.^12^ Another example are the omphalotins, head-to-tail macrocyclic dodecapeptides with nine backbone *N*-methylations, produced by the fungus *Omphalotus olearius* (Fig. 1 and S1†). Omphalotin A was identified due to its potent and selective toxicity against plant pathogenic nematodes.^13^

Our group and others have previously discovered that omphalotins are synthesized ribosomally.^14,15^ This discovery revealed a new class of ribosomally synthesized and posttranslationally modified peptides (RiPPs), termed borosins, whose hallmark are backbone *N*-methylations.^16–22^ These methylations are installed iteratively by a SAM-dependent peptide α-*N*-methyltransferase domain. In the case of the omphalotins, this domain is part of the peptide precursor protein OphMA.^14,15,23^ It has remained unclear, however, how the methylated omphalotin core peptide is released from OphMA and macrocyclized. OphP, a predicted prolyl oligopeptidase encoded by the omphalotin biosynthetic gene cluster, has been suggested to catalyze both steps.^14,15^ This hypothesis was based on the observation that coexpression of *ophP* and *ophMA* in the yeast *Pichia pastoris* was sufficient to produce omphalotin A.^15,24^ In addition, prolyl oligopeptidases catalyze the processing of other RiPP precursor peptides, including GmPOPB from the fungus *Galerina marginata* and PCY1 from the plant *Gypsophila vaccaria*.^25–27^ GmPOPB has a dual function in that it cleaves off the leader peptide and subsequently macrocyclizes the core peptide of the α-amanitin precursor peptide. In contrast, the PCY1-mediated macrocyclization of the orbitide core peptide requires prior removal of the leader peptide from the precursor peptide by a yet unidentified peptidase.^28^ Since OphP shows high sequence similarity to GmPOPB, it was predicted to also have a dual function, *i.e.* release an omphalotin intermediate from OphMA, and macrocyclize the omphalotin core peptide with concomitant removal of the follower peptide (Fig. 1). Yet, known substrates of POPs are short (<37 amino acids) unmodified peptides, while the omphalotin precursor OphMA is a protein of 417 amino acids with nine backbone *N*-methylations in the core peptide.

In this study, we present the biochemical and structural characterization of OphP. The enzyme efficiently macrocyclizes various multiply backbone *N*-methylated peptides derived from OphMA. We identified favourable residues in the P1 site, where cleavage occurs, and found that OphP processes synthetic peptides unrelated to OphMA, containing either α-*N*-methylated glycine, α-*N*-methylated alanine, or a proline residue at the P1 site, albeit with lower efficiency. The activity of OphP appears to be largely independent of the length and sequence of the follower peptide, in contrast to GmPOPB and PCY1.^25–27^ The biochemical properties of OphP are reflected in its structure, which reveals a canonical POP fold with a novel hydrophobic substrate tunnel through the β-propeller domain and a binding site accommodating the hydrophobic multiply backbone *N*-methylated substrates. The chemical synthesis of such peptides is challenging and OphP may therefore offer an enzymatic alternative for production.

## Results

### OphP macrocyclizes OphMA-derived, multiply backbone *N*-methylated peptides

OphP was produced in *P. pastoris* as an N-terminal His_8_SUMO*-fusion protein (Fig. S2†) and confirmed as catalytically active using a standard chromogenic substrate for POPs, benzyloxycarbonyl-Gly-Pro-*p*-nitroanilide (Z-Gly-Pro-4-pNA)^29^ (Fig. S3†). The activity of OphP was optimal at 30 °C and pH 6.0, and reduced by Z-Pro-prolinal (ZPP), a common covalent inhibitor for POPs^30^ (Fig. S4†). The peptide precursor protein, OphMA was produced as an N-terminal His_8_-fusion protein in *E. coli* as previously described.^14,23,31,32^ Upon 72 hours of induction, recombinant OphMA displayed predominantly 9 backbone *N*-methylations in the omphalotin core peptide.

Co-incubation of OphP with fully or partially methylated (less than 9 backbone *N*-methylations) purified OphMA did neither yield detectable peptide products (Fig. S5†) nor did the two proteins form a complex (Fig. S6†). This suggests that, in the omphalotin biosynthesis pathway, a yet-to-be-identified host protease is required to release the omphalotin intermediate from OphMA for subsequent macrocyclization by OphP (Fig. 1). Preliminary *in vivo* and *in vitro* experiments using a panel of various protease inhibitors indicate a role of the eukaryotic proteasome in the recognition and proteolytic processing of fully methylated OphMA, at least in yeast (Fig. S4†).

**Fig. 2:**
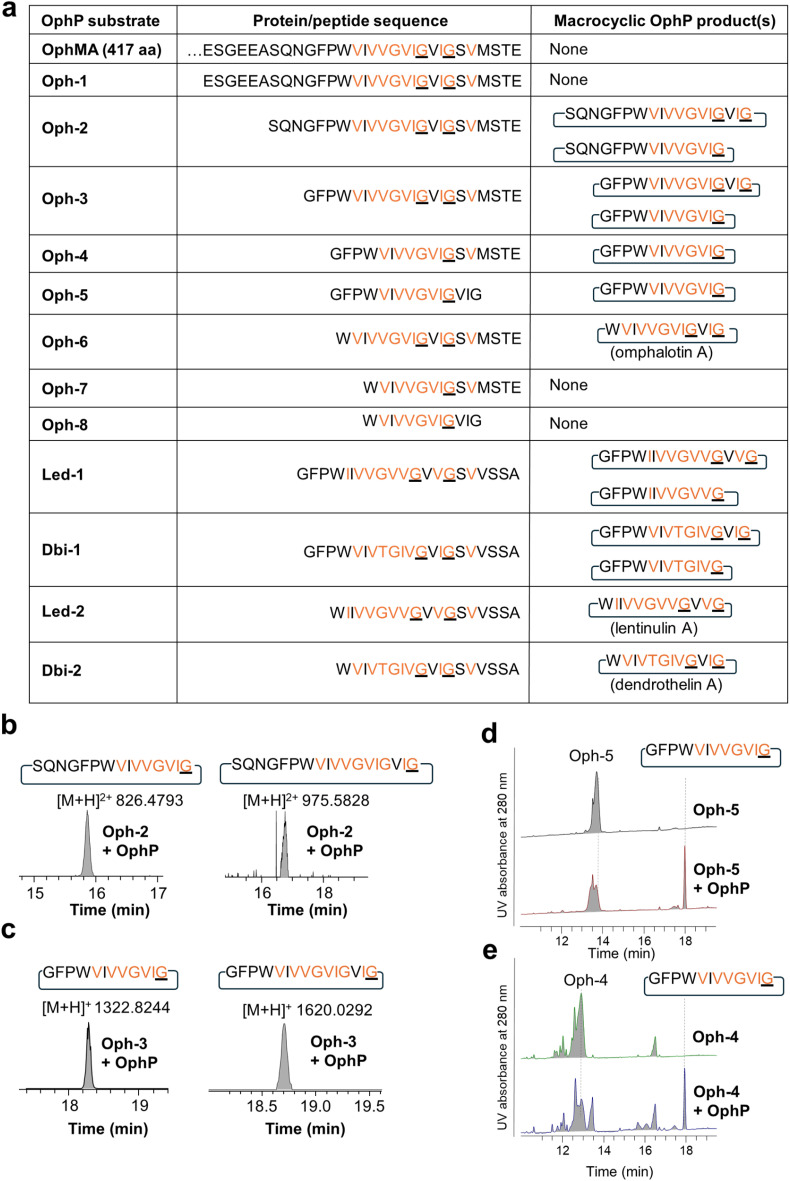
*In vitro* processing of OphMA-derived peptides by OphP. (a) Sequences of OphMA-derived OphP peptide substrates and products produced in this work, with α-*N*-methylated residues highlighted in orange. The P1 residues recognized by OphP are underlined. (b and c) Extracted ion chromatograms (EICs) of macrocyclic peptides produced by incubation of Oph-2 (b) and Oph-3 (c) with OphP. (d and e) RP-HPLC UV absorption profiles at 280 nm for Oph-5 (d) or Oph-4 (e) in the absence and presence of OphP showing the production of the same macrocyclic peptide.

We next set out to produce various OphMA-derived peptides to characterize the substrate range of OphP. Attempts to produce α-*N*-methylated or non-methylated peptides comprising the core peptide by chemical synthesis failed. Therefore, peptides were generated by proteolytic cleavage of recombinant native and genetically modified OphMA protein using trypsin and TEV protease, respectively (Fig. 2). These LC purified peptides varied in length (12 to 30 amino acids), composition, and degree of methylation (Fig. 2). The peptide substrates Oph-1 to Oph-5, included core peptides with varied length of additional N-terminal (clasp domain) or C-terminal (follower peptide) residues. Led-1 and Dbi-1 contained the 12-residue core peptides and the follower peptides (SVVSSA) of the lentinulin A and dendrothelin A precursor proteins, LedMA and DbiMA1,^24,32^ respectively (Fig. 2a and S7†). MS analysis showed that peptides Oph-1, Oph-2, Oph-3, Dbi-1, and Led-1 contained up to ten methylations, while Oph-4 and Oph-5 displayed up to 8 and 7 methylations, respectively (Fig. S7 and S8†). To produce peptides Oph-6, Led-2 and Dbi-2, we removed the clasp domain residues from Oph-3, Led-1 and Dbi-1 using GmPOPB and Proalanase®, which both cleaved the peptides after the Pro residue, but did not form macrocyclic products (Fig. 2a, S7 and S9†). Finally, Oph-7 and Oph-8 were obtained by GmPOPB or Proalanase^®^ treatment of Oph-4 and Oph-5, respectively.

Intriguingly, except for Oph-1, Oph-7 and Oph-8, incubation of all peptides with OphP yielded peptide macrocycles (Fig. 2). These macrocycles comprised, in addition to the multiply α-*N*-methylated core peptide, the respective non-methylated clasp domain residues but were devoid of the follower peptide (SVMSTE, SVVSSA) and, in case of Oph-5, the last three (non-methylated) residues of the core peptide (Fig. 2a). Incubation of Oph-2 with OphP yielded a mixture of two peptide macrocycles of 18 and 15 residues, by macrocyclization at either of the two available α-*N*-methylated glycine (^Me^Gly) residues of the omphalotin core peptide (Fig. 2b). Similarly, incubation of substrates Oph-3, Led-1 and Dbi-1, all yielded a 15-residue and a 12-residue macrocycle (Fig. 2c and S10†). Oph-4 and Oph-5 were both converted to the same 12-residue peptide macrocycle by macrocyclization at the only available ^Me^Gly residue (Fig. 2d and e). The incubation of the peptides Oph-6, Led-2 and Dbi-2 with OphP yielded omphalotin A, lentinulin A and dendrothelin A, respectively (Fig. S9†). In all reactions where peptide macrocycles were detected, respective linear peptide products were also detected (Fig. S8†). This is typical for peptide macrocyclases and a consequence of their reaction mechanism which involves cleavage of the peptide substrate after the site of macrocyclization.^25^ Incubation of peptides Oph-7 and Oph-8 with OphP only yielded linear peptide products, suggesting that OphP cannot produce a nonapeptide macrocycle, possibly due to steric hindrance.

In summary, OphP is not a dual-function prolyl oligopeptidase, but is able to efficiently cleave and macrocyclize OphMA-derived peptides containing a multiply backbone *N*-methylated core. Linear peptides of 15 to 24 residues were converted into peptide macrocycles comprising a total of 12 to 18 residues with up to six non-methylated residues preceding the core.

### OphP prefers ^Me^Gly over Gly at the P1 site of OphMA-derived peptide substrates

**Fig. 3:**
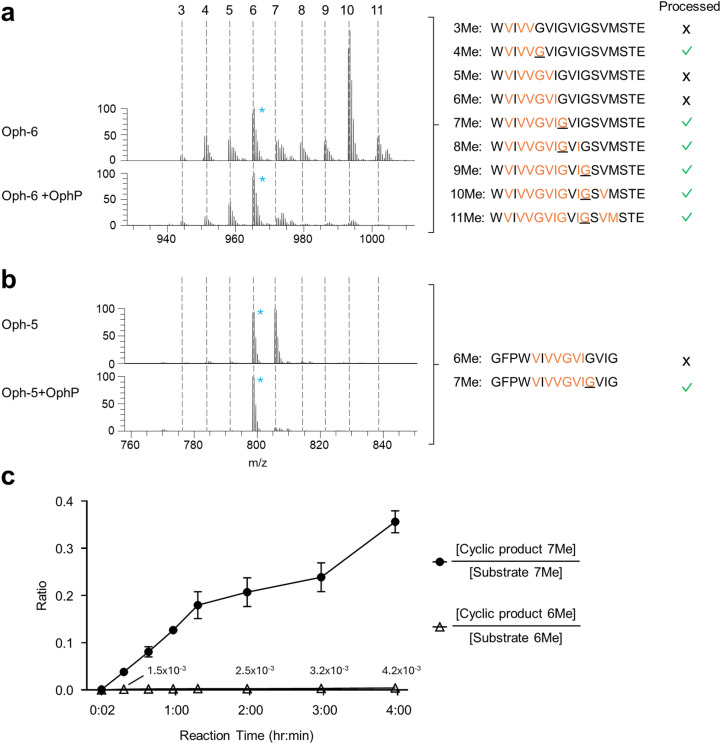
Substrate preference of OphP. (a and b) Mass spectrometric analysis showing ion chromatograms of peptide substrates Oph-6 (a) and Oph-5 (b) before and after processing by OphP. Dashed lines indicate the various methylation states of the individual peptides. For comparison, the peak height of the most abundant non-reactive species was drawn at the same level (6-fold methylated species of Oph-5 and Oph-6). These species were marked with blue asterisks. The sequence of the various peptide species and their processing by OphP is indicated on the right. Green ticks stand for processing and crosses for lack of processing by OphP. (c) Time course of *in vitro* reaction between 10 μM OphP and 100 μM total Oph-5 substrate (mixture of 6- and 7-fold methylated species) in HEPES buffer at pH 7.0. EIC peak areas of 6Me and 7Me peptide substrate and product species were measured at 8 time points over 4 hours. The ratios of EIC peak areas of cyclic product to the respective substrate with the same methylation state are shown. In comparison to the experiment shown in panels (a) and (b), a batch of Oph-5 with a higher ratio of 7Me to 6Me species was used in this experiment (see Fig. S7† for a comparison between the two batches).

Sequence analysis of substrates processed by OphP indicated that the macrocyclization occurred at ^Me^Gly residues at the P1 site. This became particularly evident by analyzing the processing of Oph-6 and Oph-5 (Fig. 3a and b).

MS analysis of purified Oph-6 showed that the peptide was predominantly 10-fold methylated but contained also less methylated peptide species in lower amounts.^14^ Incubation of this peptide mixture with OphP showed a preferred consumption of all species containing a ^Me^Gly followed by a non-methylated residue, notably the species with 4, 7, 8, 9, 10 and 11 backbone *N*-methylations (Fig. 3a).

In addition, we purified Oph-5 with equimolar amounts of 6-fold and 7-fold methylated peptide species, with the seventh methylation located at the P1 Gly. Incubation of this Oph-5 with OphP showed that the 7-fold species was much more readily consumed over the 6-fold species (Fig. 3b and S11†). The preference of OphP for the 7-fold species with a ^Me^Gly at P1 was supported by a time course experiment using another batch of Oph-5 consisting of a 1 : 10 mixture of 6- and 7-fold methylated species. After two hours of incubation with OphP the 7-fold methylated macrocycle reached over 20% of its respective substrate, compared to <1% for the 6-fold methylated species (Fig. 3c and S12†).

### OphP macrocyclizes peptide substrates unrelated to OphMA at ^Me^Gly, ^Me^Ala or Pro residues

**Fig. 4:**
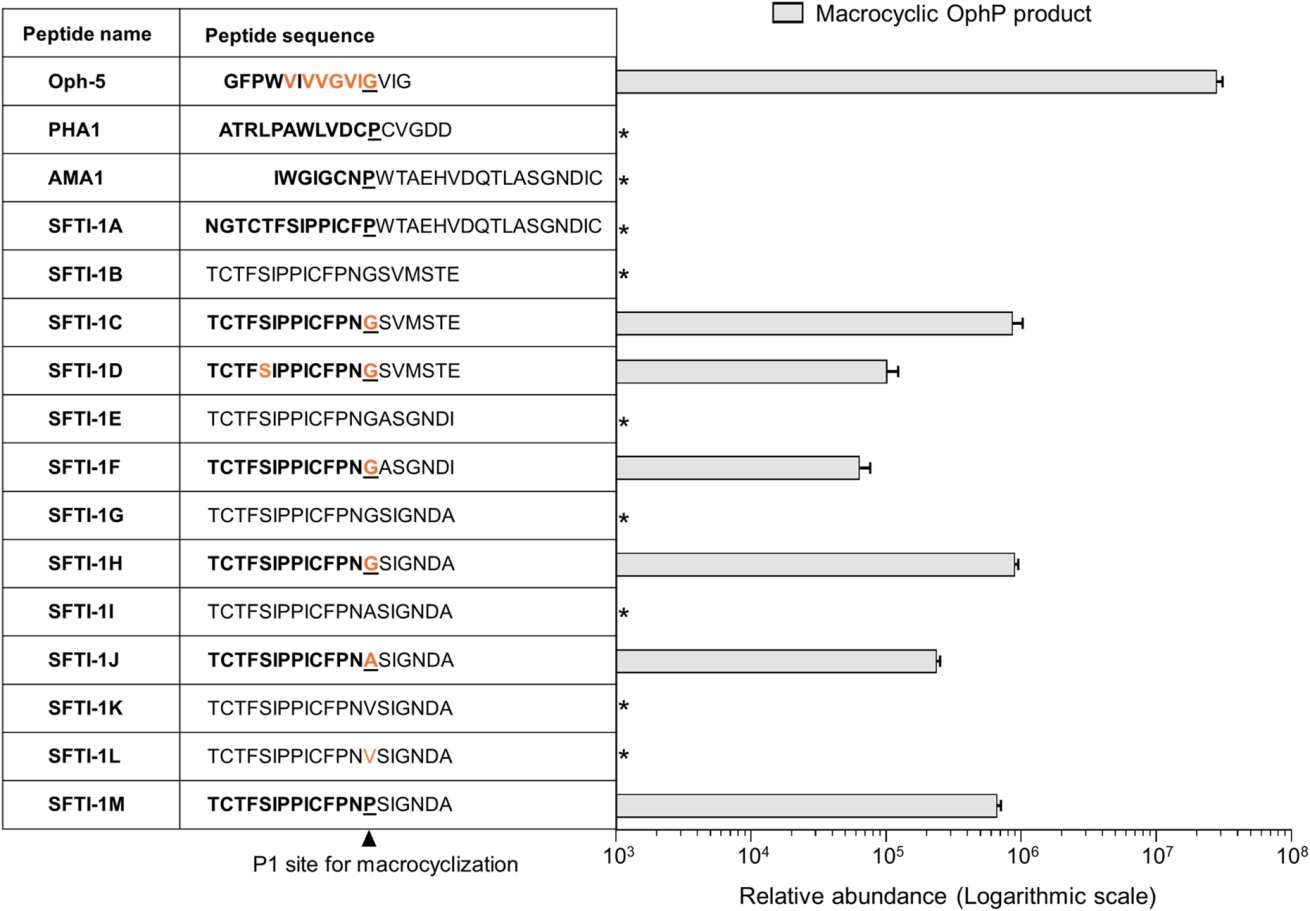
Macrocyclization of a chymotrypsin-specific Sunflower Trypsin Inhibitor-1 (SFTI-1) variant by OphP. The SFTI-1 variant was engineered by replacing the Asn residue at position 12 of variant Cpd6 (ref. 33) by the more hydrophobic Phe found in wildtype SFTI-1 and various other SFTI-1 variants. This core peptide variant was permutated to place the Gly or Pro residue at the P1 positions of the anticipated macrocyclase cleavage sites. For the other variants, the P1 residue was replaced with ^Me^Gly, Ala, ^Me^Ala, Val, ^Me^Val and Pro. Follower peptides of OphMA, AMA1, and the six amino acid sequence SIGNDA or ASGNDI were fused to the core peptides. The biotechnologically produced Oph-5 peptide was used as a comparison for processing efficiency. Formation of all products was confirmed with MS/MS (Fig. S14†). Relative comparison of macrocyclic product (in bold) formation from the SFTI-1 fusion peptides at the expected P1 site by the fungal macrocyclase OphP. Relative abundance was measured as EIC peak area of the respective products. *Product not observed.

To explore the substrate recognition by OphP further, we assessed the *in vitro* processing of synthetic peptides whose sequences were unrelated to OphMA (Fig. 4).

First, we tested the processing of α-amanitin (AMA1) and phallacidin (PHA1) precursors by OphP. Neither of these peptides, which were both readily processed by GmPOPB, was processed by OphP (Fig. 4, S13 and S14†).

Next, we chemically synthesized a panel of 13 peptides whose core sequences are related to the cyclotide sunflower trypsin inhibitor-1 (SFTI-1), a plant RiPP^33^ (Fig. 4 and S14†). We permutated the SFTI-1 core peptide sequence to have either a proline (SFTI-1A) or a glycine residue at the C-terminus (SFTI-1B) and added the α-amanitin and the omphalotin A follower peptide sequences, respectively. Of SFTI-1B, two additional versions, one with an ^Me^Gly at the P1 site (SFTI-1C) and another one with both an ^Me^Gly at the P1 site and an ^Me^Ser in the core peptide (SFTI-1D) was synthesized. Neither SFTI-1A nor SFTI-1B was processed by OphP. OphP did, however, macrocyclize the core peptides of SFTI-1C and SFTI-1D, using the ^Me^Gly as P1 site, albeit with an approximately 100-fold lower efficiency than Oph-5 (Fig. 4).

Next, we tested variants of SFTI-1B with either Gly or ^Me^Gly residues at the P1 site and six residue follower peptides ASGNDI and SIGNDA (derived from the AMA1 follower peptide). From these peptides (SFTI-1E to SFTI-1H), again only the variants containing ^Me^Gly at the P1 site were macrocyclized by OphP (Fig. 4). The processing efficiency of these peptides was similar to that of SFTI-1C and SFTI-1D carrying the follower peptide of OphMA. For peptides with Ala, ^Me^Ala, Val, ^Me^Val or Pro at P1 site (SFTI-1I to SFTI-1M), OphP successfully macrocyclized substrates containing ^Me^Ala or Pro at the P1 site, but not those with ^Me^Val, Val or Ala (Fig. 4 and S14†). Based on the processing efficiency, the preference of OphP for residues at the P1 position of this peptide substrate is ^Me^Gly > Pro > ^Me^Ala.

**Fig. 5:**
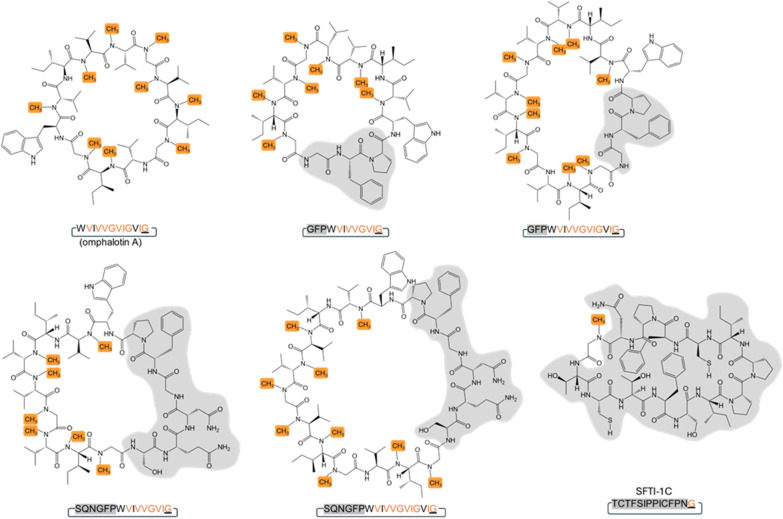
Structures of peptide macrocycles produced by OphP. Peptide macrocycles obtained by *in vitro* processing of OphMA-derived and the OphMA-unrelated SFTI-1C peptide substrates. Backbone *N*-methylated residues are highlighted in orange and residues differing from omphalotin A are highlighted in grey.

These results show that OphP can macrocyclize peptides that are not related to the omphalotin A core peptide if they contain an ^Me^Gly, ^Me^Ala, or a Pro residue at the P1 site (Fig. 4, 5). Interestingly, GmPOPB processes the same peptides but with different preference for the P1 residues (Pro > ^Me^Ala > ^Me^Gly) (Fig. S14†). However, GmPOPB does not process the multiply backbone *N*-methylated peptide Oph-5 whilst OphP does not convert AMA1 and PHA1, indicating substrate recognition is not simply the P1 residue. In case of GmPOPB and PCY1, the follower peptide of the native substrates plays an important role, which is in agreement with the different processing efficiency of GmPOPB for peptides SFTI-1A and SFTI-1M (Fig. S14†). Conversely, the processing efficiency of OphP was not obviously influenced by the follower peptide sequence (Fig. 4).

### OphP adopts a canonical POP-fold with a wide and hydrophobic central tunnel in the β-propeller domain and a small and hydrophobic P1 binding pocket in the hydrolase domain

**Fig. 6:**
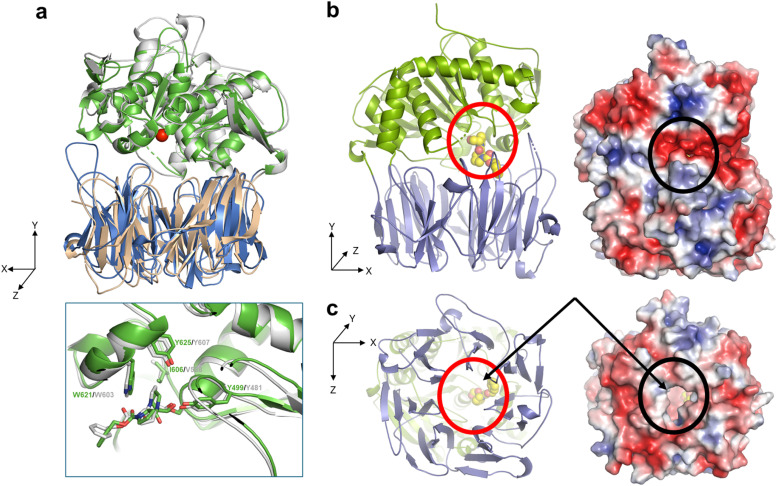
Crystal structures of OphP complexed with ZPP covalently bound to S580. (a) Overlay of OphP:ZPP and PCY1:ZPP complex (RCSB 5UW6 (ref. 26)) with the domains of OphP colored green and blue. The active site serine (580 in OphP) is shown as red ball. Inset: close up comparison of the ZPP:serine adducts of OphP and PCY1, residues surrounding the ZPP proline closest to the serine are shown and labelled. (b) In all structures in this study, OphP is found with the two domains close together (so called closed state). The β-propeller domain is in blue and the hydrolase domain in green. The active site is located at the interface of the domains. A molecule of the inhibitor ZPP (carbon atoms yellow, oxygen red, and nitrogen blue) shown in sphere form has been placed at the active site circled in red to aid visualization. Experimental details of the ZPP complex structure are shown in Fig. S16.† On the right is the electrostatic surface calculated by the APBS-PDB2PQR software suite^40^ under the default setting and drawn in PyMol. The electrostatic potential is set at ±5*kT*/*e*. There is no access to the active site (black circle) from the side of the structure in the closed state. (c) Rotation of 90° around the *X*-axis shows there is a wide and hydrophobic tunnel through the β-propeller domain (circled in red in cartoon). On the right is the electrostatic surface calculated as above, the tunnel allows access to the active site (black circle). The yellow carbon atoms of ZPP can be seen through the pore in both representations.

To assess the structural basis for the substrate recognition by OphP, the crystal structure of the catalytically inactive OphP(S580A) was determined in space group P1 (eight monomers in the asymmetric unit) at 1.9 Å by molecular replacement using GmPOPB (PDB entry 5N4C) as a search model. OphP adopts a canonical POP-fold with two domains, an α/β hydrolase domain (residues 1–82, 453–738) and a seven-bladed β-propeller domain (residues 83–452) The structure of OphP is most similar to GmPOPB (5N4B) with a root-mean-square-deviation (RMSD) of 1.5 Å over 686 residues and closely related to PCY1 (5O3W, RMSD of 1.7 Å over 650 residues), and more distant to porcine muscle POP (1QFS, RMSD of 1.7 Å over 650 residues). The PISA server^34^ suggests that OphP, like other POPs, is a monomer. The eight OphP(S580A) monomers in the asymmetric unit differ only in flexible loops, of which some are involved in crystal contacts. They all adopt a “closed” conformation in the crystal with the hydrolase and the β-propeller domains tightly packed against each other, preventing peptide substrate added to the crystal from entering the active site through a “side entrance” (Fig. 6b). Based on these structural features, we speculate that peptide substrates reach the active site of OphP *via* an alternative route, that is independent of the “hinging” between the hydrolase and the β-propeller domain claimed for other POPs^25,35–39^(Fig. 6c and S15†).

OphP crystals soaked with covalent inhibitor ZPP overnight showed density at the active site. The structure of the protein was resolved at 2.0 Å with clear electron density for covalently linked ZPP (Fig. S16†). The OphP:ZPP complex structure is largely unchanged from apo OphP (0.5 Å of RMSD over 716 Cα atoms). As with the apo structure, significant structural rearrangement of loops is required to position His701 to act in the triad (7.6 Å to Ser580). Non-optimal arrangement of the triad residues was also seen in PCY1 ^26,27^ and GmPOPB.^25^ A comparison with the PCY1:ZPP structure reveals a very similar arrangement of the adduct at the active site. The hydroxyl group of Tyr499 and the backbone amide of Asn581 are well-positioned to function as the canonical oxyanion hole pair to stabilize the tetrahedral acyl enzyme intermediate with possible additional stabilization by Arg667. The proline group of ZPP adjacent to the covalent link sits in a pocket formed by Phe502, Ile606, Trp621 and Tyr625. The second proline of ZPP makes no contacts with the protein. The benzene ring of ZPP is partially disordered and located in a hydrophobic pocket formed by Phe204, Ile264, and Phe617. The residues surrounding the proline group adjacent to the covalent link are identical and in similar positions as in all the other POP enzymes (Fig. S17 and S18†). The only exception is Ile606 in OphP, which is found as Val in the other POPs *e.g.* as Val588 in PCY1 and Val604 in GmPOPB. The larger side chain of Ile makes the P1 binding pocket of OphP smaller which may select for P1 residues with a small side chain.

### Structures of complexes between OphP(S580A) and OphMA-derived substrates Oph-6 and Oph-5 reveal a large hydrophobic substrate binding site

**Fig. 7:**
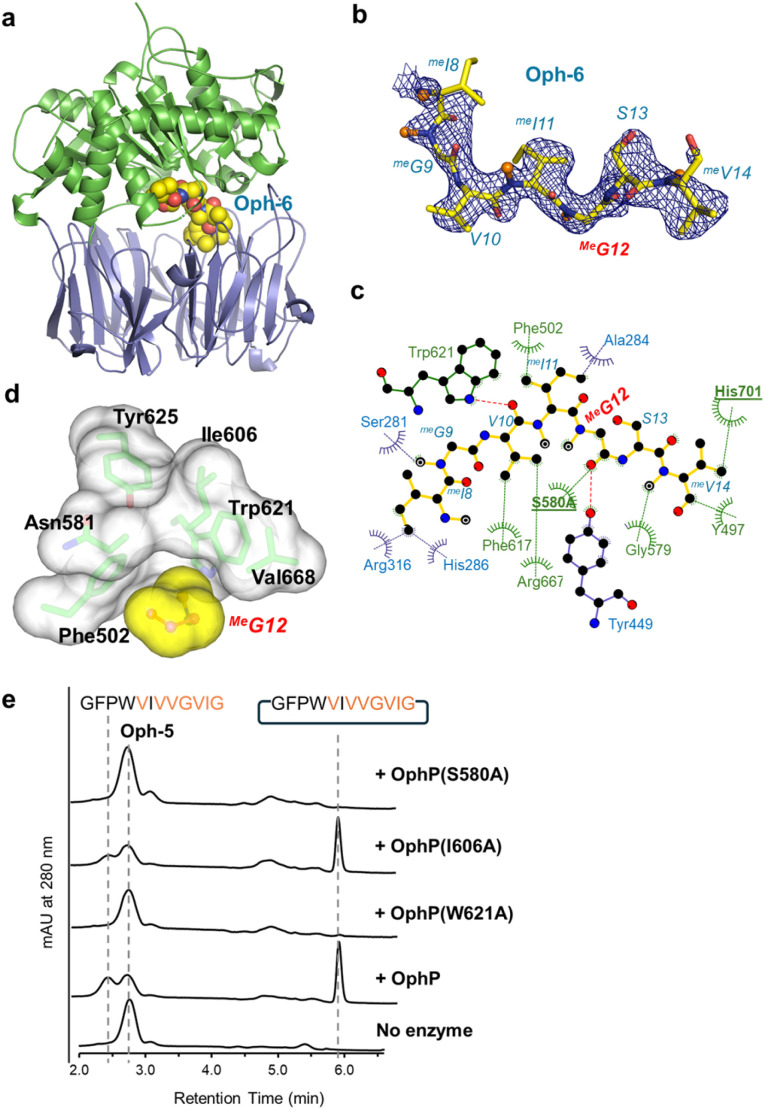
Structure of the P1 recognition site of OphP. (a) OphP S580A complex with peptide substrate Oph-6 is shown in spheres (the color scheme is as in Fig. 6a). (b) The σ_A_-weighted 2mFo-DFc map (contoured at 1σ) for Oph-6 (c) LigPlot diagram^45^ showing interactions of Oph-6 with OphP. (d) Surfaces of ^Me^Gly12 and its surrounding hydrophobic pocket in the Oph-6 complex. The pocket is composed of Tyr625, Trp621, Val668, Phe502, Asn581 and Ile606. (e) HPLC analysis of assays using 7-methylated Oph-5 as substrate for OphP and its variants. The control reaction without OphP shows the absorption profile of the starting material. While the W621A mutation abolished macrocyclization, I606A showed an activity comparable to wildtype OphP. The reactions were performed at 25 °C in buffer containing 50 mM HEPES pH 7.0, 100 mM NaCl and 5 mM DTT at a concentration of 20 μM enzymes and 200 μM Oph-5.

Substrate complexes of OphP were obtained by soaking apo OphP(S580A) crystals with Oph-6 (10× Me) or Oph-5 (7× Me) at 2.0 and 2.5 Å resolution, respectively (Fig. 7a–c and S18†). The protein structures superimpose with the apo structure with an RMSD of 0.4 Å over 715 Cα atoms (Oph-6) and 0.8 Å over 714 Cα atoms (Oph-5). For the OphP:Oph-6 complex structure, we were able to fit residues ^Me^I8 to ^Me^V14 to the electron density whilst the remainder were presumed to be disordered (Fig. 7b) (for ease of discussion, peptide residues are referred to by single letter code while three letter codes are used for protein residues). The peptide substrates were located in the active site between the hydrolase and the β-propeller domains, with their N-termini extending into the central tunnel of the β-propeller domain and their C-termini contacting the hydrolase domain (Fig. 7a and S18†). In comparison to other structurally characterized POPs, the central tunnel in the β-propeller domain of OphP(S580A) is significantly wider at its entrance and more hydrophobic.

Attempts to soak synthetic peptides corresponding to the non-methylated clasp domain and the follower peptide failed with no observable density for peptides. Similarly, isothermal titration calorimetry (ITC) measurements of OphP to synthetic peptides corresponding to the non-methylated clasp domain and the follower peptide did not indicate any binding, in contrast to Oph-5 which was bound with μM affinity (Fig. S20†). The hydrophobic peptide and its α-*N*-methyl groups are accommodated by a series of aliphatic or aromatic amino acids of OphP. The side chain of ^Me^I8 packs against the aliphatic part of the Arg316 side chain whilst ^Me^G9 makes almost no contact. The side chain of V10 interacts strongly with the aromatic ring of Phe617, ^Me^I11 stacks with Phe502 whilst its *N*-methyl group points to the solvent (Fig. 7c). ^Me^G12 sits in the P1 pocket with its carbonyl group positioned for nucleophilic attack by S580. The α-*N*-methyl of the amide of ^Me^G12 makes van der Waal contacts with the indole of Phe502 and sits in a pocket formed by Phe502, Ile606 and Trp621 (Fig. 7d). The Cα of ^Me^G12 is surrounded by Ile606, Val668, Ser580 (<5 Å), Trp621 (6 Å) and the hydroxyl group of Tyr625 (7 Å). In the Oph-5 complex, residues G1 to ^Me^I11 were placed in the electron density (Fig. S19†). However, the positions of the peptide bonds of the substrate near Ser580 are inconsistent with a pre-attack state (Fig. S19†), perhaps representing another conformation during catalysis.

To confirm the functional relevance of some of the interacting residues in the P1 site, we produced, in addition to S580A, OphP active site variants I606A and W621A. Incubation with Oph-5 showed that I606A is only slightly less active than the native enzyme, while W621A had, similar to S580A, no activity (Fig. 7e). We were unable to purify substrate variants of Oph-5 with G12V, G12L or G12A variations at the P1 site, whose processing would have provided information about the preference of OphP regarding this residue in the context of its native substrate. This is one of the reasons why we switched to SFTI-1-related substrates (Fig. 4).

Taken together, binding of the hydrophobic, multiply backbone *N*-methylated native substrates appears to be mediated by a plethora of interactions with residues at and near the active site of OphP. In contrast to the other characterized RiPP macrocyclases,^26,27,41–44^ OphP appears to not utilize the C-terminal follower peptides (or N-terminal leader peptides) as a recognition sequence but rather binds to the hydrophobic, multiply backbone *N*-methylated core peptide.

## Discussion

In this study, we present the biochemical and structural characterization of the hitherto uncharacterized S9A serine protease OphP involved in the biosynthesis of omphalotin A. We show that the recombinant enzyme is a bona-fide peptide macrocyclase acting on OphMA-derived multiply backbone *N*-methylated peptides. We could not, however, detect any activity of OphP towards the full-length precursor protein OphMA (Fig. S4†), suggesting that the initial endoproteolytic cleavage of OphMA and release of the omphalotin A intermediate is accomplished by a yet-to-be-identified host protease (Fig. 1). Our preliminary results indicating the involvement of the eukaryotic proteasome in this process (Fig. S4†) are in accordance with the finding that bacteria coexpressing OphMA and OphP, are much less efficient in the production of omphalotin A than yeast (Fig. S21†).

Structures of OphP-substrate complexes were obtained by soaking crystals of the apo enzyme with peptide substrates (Fig. 6, 7 and S19†). The cavity created by “hinging” of the hydrolase and the β-propeller domain in other POPs^25,35–39^ was not accessible in the OphP crystals (Fig. 6c and S15†) and, thus, the substrate had to enter OphP *via* a different route. One possible route is through a side portal between the hydrolase and β-propeller domain, seen in dipeptidyl peptidase IV.^35–37^ However, the portal in this enzyme is much larger than the one in OphP. Additionally, the disordered loops Pro222 to Gly230 and Leu697 to Gly704 in OphP are expected to further constrict this route. Moreover, the side cavity in OphP is highly polar and thus hydrophilic, which is in marked contrast to the OphMA-derived substrates (Fig. 6b). Instead, we favour the tunnel as the substrate entry route, supported by the unusually wide central hydrophobic tunnel (Fig. 6b and S18†) which matches the substrate. Such a tunnel entry route has not been observed for any member of the POP family.

The use of a variety of OphMA-derived and non-OphMA-related peptide substrates for OphP showed that a ^Me^Gly residue is favoured over non-methylated Gly, ^Me^Ala and Pro at the P1 site (Fig. 2–4). In contrast, GmPOPB prefers Pro over ^Me^Gly and ^Me^Ala (Fig. S14†). Backbone *N*-methylated amino acids resemble Pro in the sense that both are tertiary amides and thus occupy the P1 pocket in a similar fashion (Fig. 7). Our structural data shows that there are favourable hydrophobic interactions for the methyl group of *N*-methyl amides which are absent in non-methylated Gly or Ala residues or the OphP(W621A) variant (Fig. 7). A comparison between OphP and GmPOPB shows that the P1 binding pockets are overall very similar except for the substitution of Val604 in GmPOPB (and other POPs) by Ile606 in OphP that might account for the slight difference in P1 residue preference of the two enzymes (Fig. S18†).

Analysis of the processing of the various peptide substrates by OphP showed that the efficiency of the enzyme is largely independent on the length or sequence of the follower peptide (Fig. 2a and 4). Consistent with this, the structural analysis of the enzyme–substrate complexes showed no specific interaction of OphP with residues C-terminal to the core peptide (Fig. 6 and 7). Taking the ITC measurements (Fig. S20†) into account, we conclude that OphP, in contrast to the other characterized RiPP macrocylases,^27,46–49^ does not recognize a follower or leader sequence, but rather binds to the hydrophobic, multiply backbone *N*-methylated core peptide. PCY1 was previously shown to macrocyclize peptides with up to two backbone *N*-methylations, although at the cost of increased hydrolysis compared to the desired transamidation and requiring a C-terminal recognition sequence.^49^

In summary, we determined the catalytic activity and specificity as well as the crystal structure of the prolyl oligopeptidase OphP involved in the biosynthesis of the fungal RiPP omphalotin. The distinctive structural features of OphP include a wide hydrophobic substrate tunnel, an active site with a small P1 binding pocket and a large hydrophobic substrate binding site. These features account for the unique recognition of the core instead of a follower, with a preference for hydrophobic, backbone *N*-methylated core peptides with a ^Me^Gly at the P1 position. The provided molecular insights may pave the way to the use of OphP as a tool for the biotechnological production of multiply α-*N*-methylated peptide macrocycles for pharmaceutical and agricultural applications.

## Author contributions

The individual author contributions (according to CRediT taxonomy) were: EM, HS and LS: methodology, validation, formal analysis, investigation, writing (original draft preparation) and visualization; FG: validation and investigation; AMG and SL: investigation and formal analysis; ADG: methodology, resources, supervision and writing (reviewing and editing); MK and JHN: conceptualization, methodology, funding acquisition, project administration, resources, supervision, writing (reviewing and editing).

## Conflicts of interest

MK is an inventor on a patent application filed by ETH Zurich (no. WO2017174760A1, priority date: 7 April 2016). The authors declare no other competing interests.

## Supplementary Material

SC-016-D5SC03723A-s001

SC-016-D5SC03723A-s002

## Data Availability

Crystallographic and structural data of OphP are available from the RCSB (https://www.rcsb.org/); 7ZB2 (apo-structure), 7ZAZ (ZPP complex), 7ZB0 (Oph-5 complex), 7ZB1 (Oph-6 complex). Raw data for the figures are shown in the ESI.† UniProt accession numbers of described proteins; OphMA: A0A2R2JFI5, OphP: P9WEN5, GmPOPB: H2E7Q8, PCY1: R4P353.
