# Supplementary material for: Macrocyclization of backbone N-methylated peptides by a prolyl oligopeptidase with a distinctive substrate recognition mechanism
Source: Chem Sci. 2025 Jul 2;16(31):14196–206. doi: 10.1039/d5sc03723a (PMC12235650; doi:10.1039/d5sc03723a)
Supplement: SC-016-D5SC03723A-s001 [file SC-016-D5SC03723A-s001.pdf]

Supplementary Information (SI) for Chemical Science.
This journal is © The Royal Society of Chemistry 2025

Contents

Figure S1. Conservation of omphalotin-related biosynthetic gene clusters............................2

Figure S2. OphP production and purification in *Pichia pastoris*...3

Figure S3. OphP activity towards the chromogenic substrate Z-Gly-Pro-pNA. 4

Figure S4. Susceptibility of *in vivo* and *in vitro* production of omphalotin-related peptides towards protease inhibitors. 6

Figure S5. Lack of conversion of completely methylated, full-length OphMA by OphP *in vitro*. 8

Figure S6. Analysis of complex formation between His_8OphMA and His_8SUMO*OphP 10

Figure S7. HPLC-MS analysis of OphP substrates...11

Figure S8. LC-MS/MS spectra of OphP peptide substrates and products. 13

Figure S9. Activities of OphP, LedP, GmPOPB and ProAlanase towards Oph-5 23

Figure S10. HPLC-MS of macrocyclic OphP peptide products. 25

Figure S11. Preference of OphP for highly methylated peptide substrates by variation of enzyme:substrate ratio...26

Figure S12. Preference of OphP for highly methylated peptide substrates by a time course of substrate depletion and product formation. 27

Figure S13. Activities of GmPOPB and OphP towards synthetic peptides PHA1 and AMA1. 32

Figure S14. Overview, relative abundance and LC-MS/MS spectra of macrocyclic peptide products 33

Figure S15. Cartoon and electrostatic surfaces of apo OphP (PDB 7ZB2), apo GmPOPB (PDB 5N4F) and apo human dipeptidyl peptidase IV (DPP-IV, PDB 1NU6). 41

Figure S16. Crystal structure of the OphP:ZPP covalent inhibitor complex. 42

Figure S17. Multiple sequence alignment of OphP with post-proline cleaving prolyl oligopeptidases (POPs). 43

Figure S18. Surface of the P1 pocket of GmPOPB (PDB 5N4C), PCY1 (PDB 5UW6) and OphP (PDB 7ZAZ and 7ZB1) 45

Figure S19. Crystal structures of OphP in complex with Oph-5. 46

Figure S20. ITC experiments between OphP(S580A) and residues from the clasp domain (MPSSLLDAARESGEEASQNGFP), follower peptide (SVMSTE, and VIGSVMSTE), and Oph-5. 47

Figure S21. Production of omphalotin A in *P. pastoris* and *E. coli*. 48

References 49

Figure S1. Conservation of omphalotin-related biosynthetic gene clusters. Top right: Schematic representation of the homologous biosynthetic gene clusters from *Omphalotus olearius* (CBS102282, scaffold 137), *Dendrothele bispora* (CBS962.96_v1.0, scaffold 621) and *Lentinula edodes* (Le(bin)0899 ss11, scaffold 10). Genes in the conserved regions of the clusters are shown with colored block arrows. The legend indicates predicted functions or domains of the encoded proteins and the respective protein names in *O. olearius*. NTF2: Nuclear transport factor 2, MBOAT: Membrane bound O-acyltransferase. The DNA scaffolds and gene models were obtained from JGI Mycocosm (https://mycocosm.jgi.doe.gov/) for *D. bispora* and *L. edodes*, and from the Syngenta natural products consortium[1] (GenBank PV026309.1) for *O. olearius*. Left and bottom right: Structure of omphalotin variant A and the modifications of variants B-I.

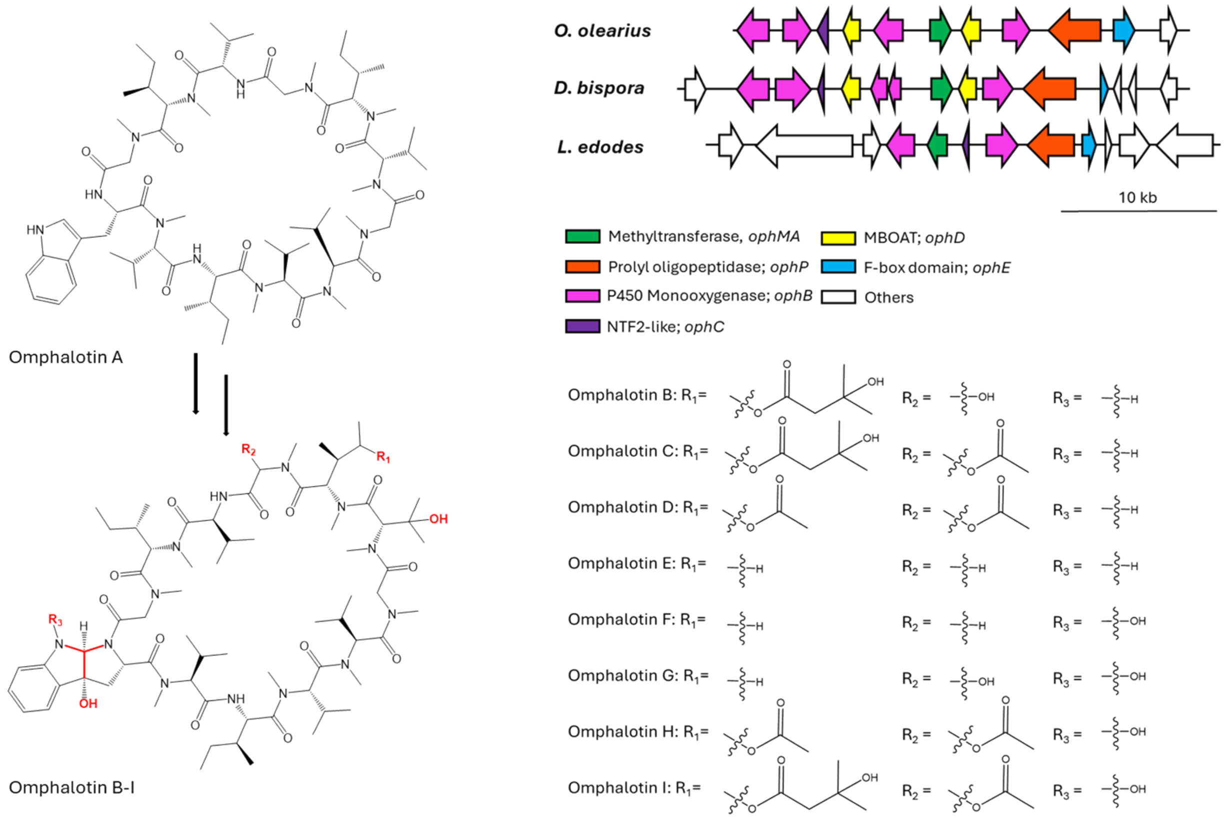

[1] BBSRC-Syngenta Natural Products sLoLa consortium (BB/K002341/1)

Figure S2. OphP production and purification in *Pichia pastoris*. (**a**) OphP was fused to a His8-SUMO* via a cleavage site for TEV protease (in red). Residues Thr and Ser after the TEV cleavage site resulted from the insertion of the restriction site *Spe*I between OphP and the tag. The arrow indicates the cleavage site for TEV protease. (**b**) Purification of OphP. After purification of His$_8$SUMO*-OphP using metal affinity chromatography (lane MA), the tag was cleaved off by incubation of the purified fusion protein with TEV protease at room temperature (lane RT+TEV). We noted that the tag was also slowly cleaved off even without the addition of TEV protease (lane RT). The P1 site of latter cleavage was mapped to residue Thr117 (**a**) by whole protein MS. The OphP protein without tag was further purified by size exclusion chromatography (SEC) using an Aekta FPLC system. **(c)** OphP peak from SEC purification consistent with a monomer. mAU: Milli Absorbance Unit. (**d**) Heterologous production and purification of OphP homologs LedP and GmPOPB. Recombinant LedP was produced in *P. pastoris* (this study) and GmPOPB in *E. coli*[1].

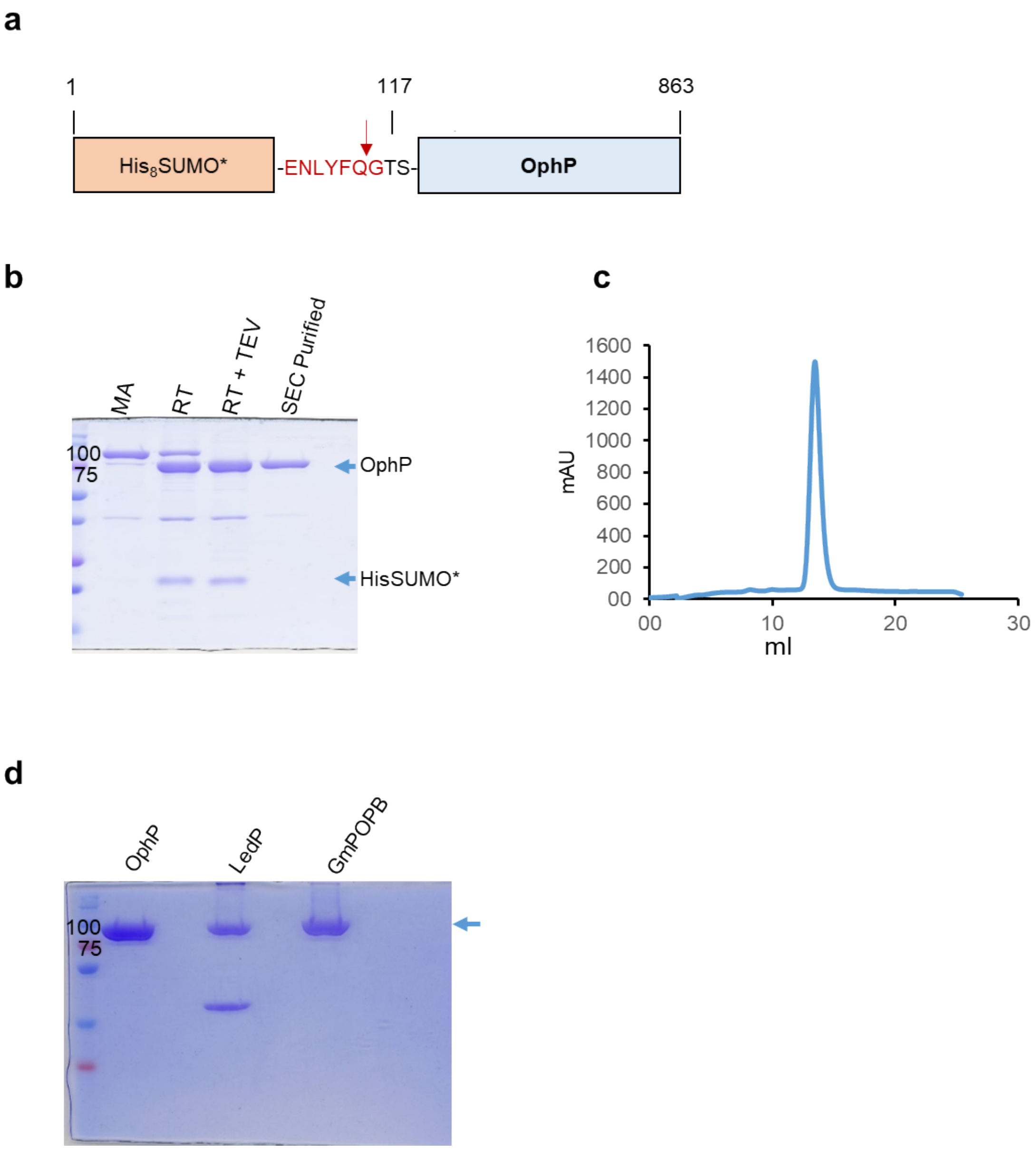

Figure S3. OphP activity towards the chromogenic substrate Z-Gly-Pro-pNA. (**a**) The effect of temperature. The reactions were run in the standard assay buffer (50 mM HEPES pH 6.0 + 10 mM DTT). (**b**) The effect of pH (at 30°C). In both assays, (a) and (b), the concentrations of the substrate and the enzyme were 1 mM and 8 µM, respectively. All assays were performed in triplicates overnight. The enzymatic activity was monitored by measuring the absorbance at 410 nm on an Infinite 200 Pro M Plex spectrophotometer. Error bars indicate standard errors of the mean. (**c**) Conversion kinetics of Z-Gly-Pro-pNA by OphP. On the left, the calibration curve of pNA, and on the right, enzyme saturation assay are shown. For the substrate calibration curve, the reactions were incubated at 30°C with 8 µM OphP for 22h. A linear curve was fitted to the data points and the resulting linear equation A_{410} = εm x C + b was used to calculate pNA concentration as a function of absorbance (C = (A – b) / εm, where εm is the molar extinction coefficient of pNA. The curve was fitted with an R-squared value of 0.9831. For the enzyme saturation assay, pNA product was measured at various times for four different OphP concentrations, 4 µM (0.5x[Enz]), 8 µM (1x[Enz]), 16 µM (2x(Enz]), 40 µM (5x[Enz]) and 1mM of substrate. The curves for the 2x and 5x relative to substrate concentrations of OphP were outside of the linear range after 4h and 10h, respectively.

a

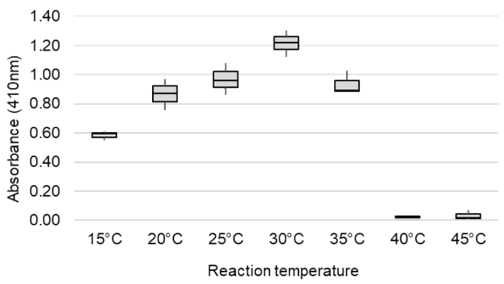

b

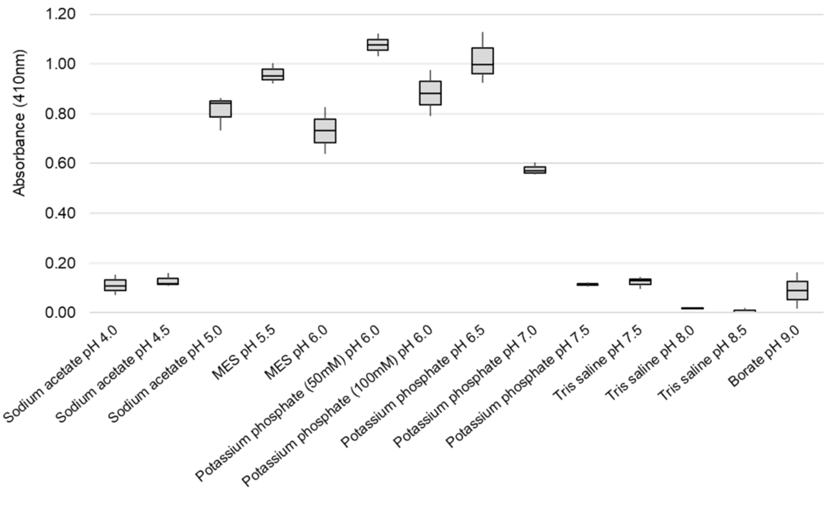

c

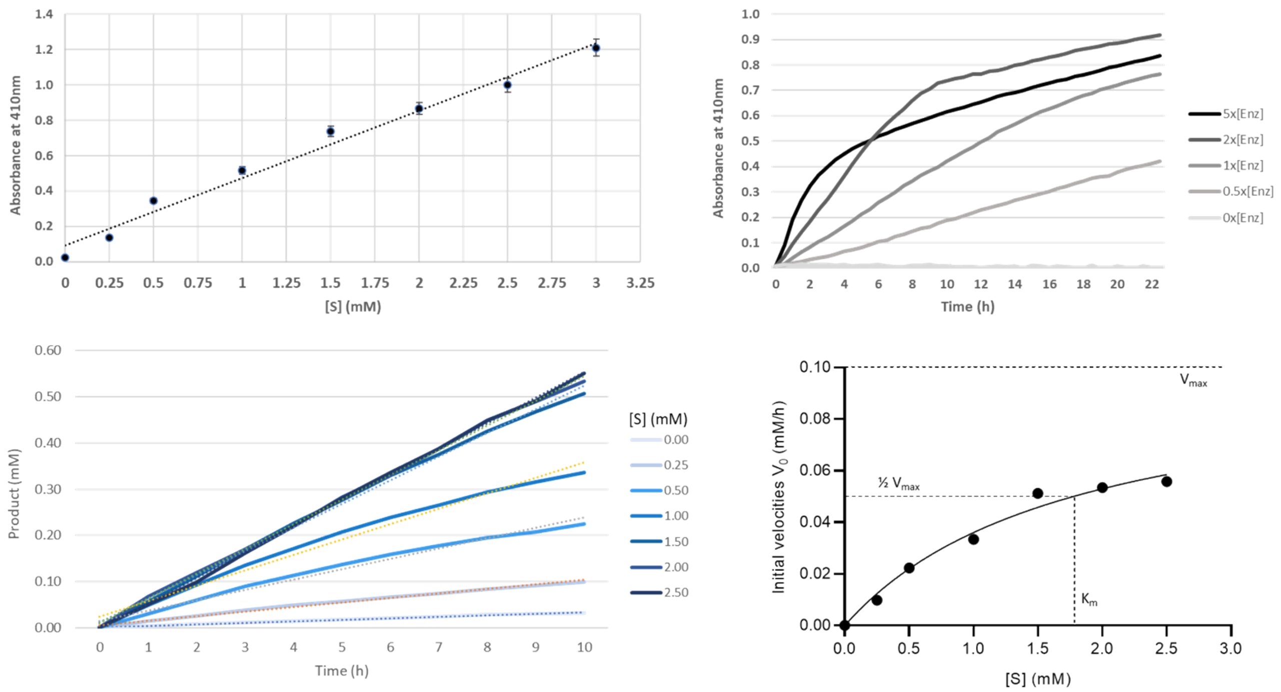
Absorbance at 410nm
[S] (mM)
Absorbance at 410nm
Time (h)
5x[Enz]
2x[Enz]
1x[Enz]
0.5x[Enz]
0x[Enz]
Product (mM)
Time (h)
[S] (mM)
0.00
0.25
0.50
1.00
1.50
2.00
2.50
Initial velocities V0 (mM/h)
[S] (mM)
Vmax
½ Vmax
Km

Figure S4. Susceptibility of *in vivo* and *in vitro* production of omphalotin-related peptides towards protease inhibitors. (**a**) *In vivo* production of omphalotin A in *P. pastoris* co-expressing *ophMA* and *ophP* as described previously[2], with addition of different protease inhibitors in DMSO at the time of induction of expression. Omphalotin A concentrations were quantified by integrating the Extracted Ion Chromatogram (EIC) peak area of HPLC-MS measurements of cell lysates of triplicate cultures. Ctrl: Control. (**b**) *In vitro* production of 6- and 7-fold methylated linear and macrocyclic peptide product species with 100 µM total Oph-5^{high} peptide substrate and 10 µM OphP concentration, in presence of different protease inhibitors. NOTE: The batch of Oph-5 ^{high} used for these experiments was the same as used in Figure 3bcd (1:10 batch, see Figure S4a). The reaction was performed in HEPES buffer at pH 7.0 and stopped after 30 minutes. Products were quantified using the EIC peak area of HPLC-MS measurements. (**c**) *In vitro* production of 6- and 7-fold methylated linear and macrocyclic peptide products with 100µM total Oph-5^{high} peptide substrate and 10µM OphP enzyme concentration, in presence of two different concentrations of ZPP. The reaction was performed in HEPES buffer at pH 7.0 and stopped after 30 minutes. Peptide species were quantified by integrating the EIC peak area of the HPLC-MS measurements.

a

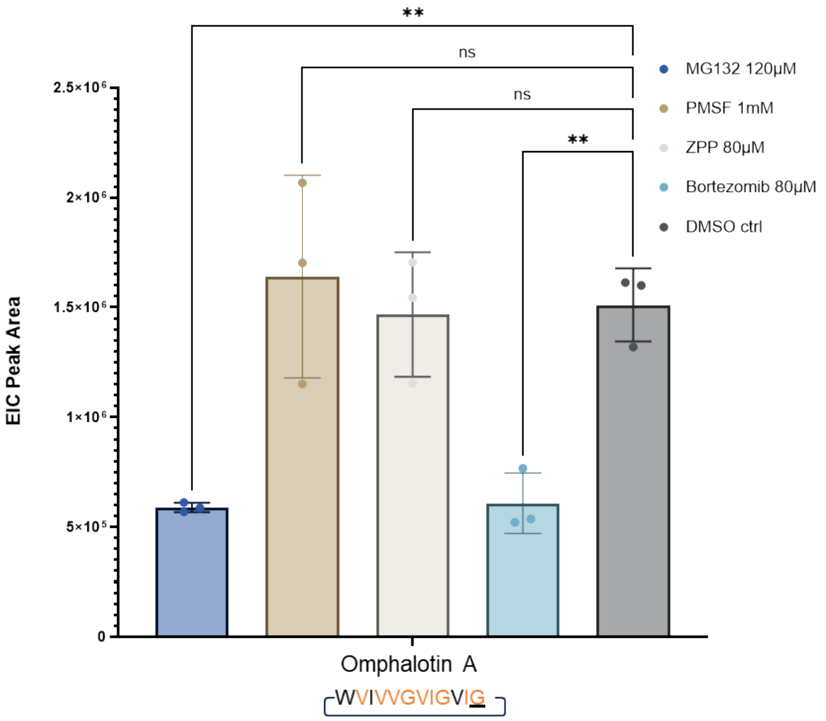

b

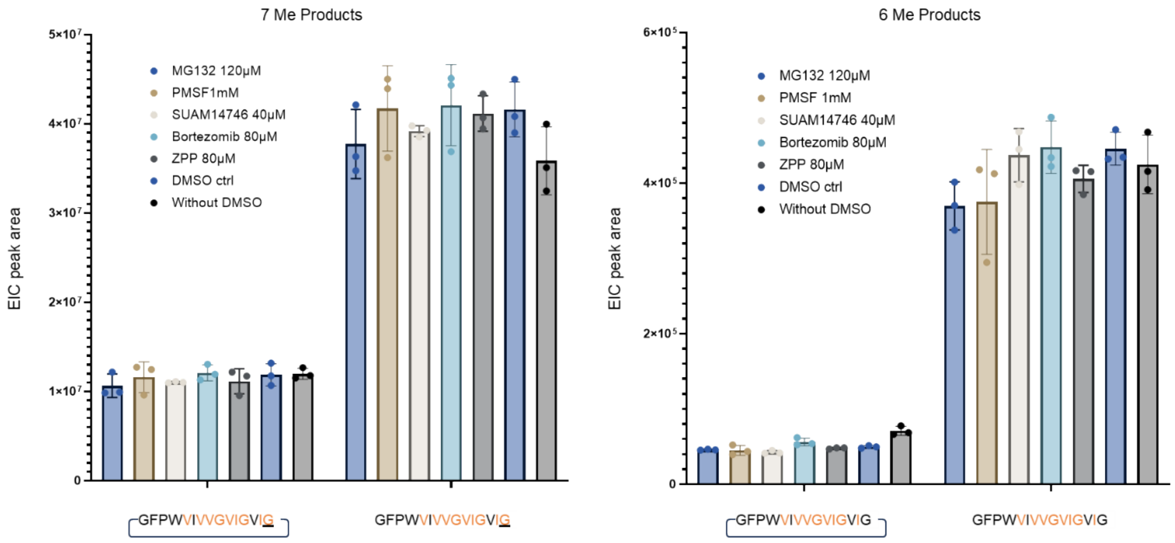

7 Me Products
6 Me Products
EIC peak area
MG132 120μM
PMSF1mM
SUAM14746 40μM
Bortezomib 80μM
ZPP 80μM
DMSO ctrl
Without DMSO
PMSF 1mM
GFPWVIVVGVIGVIG

c

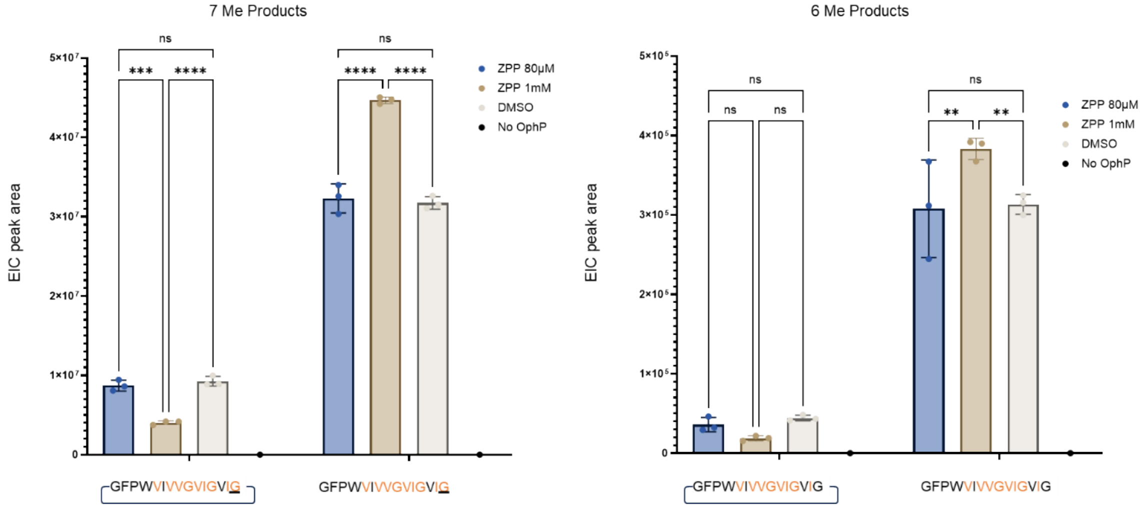

7 Me Products
6 Me Products
EIC peak area
ZPP 80μM
ZPP 1mM
DMSO
No OphP
ns
GFPWVIVVGVIGVIG

Figure S5. Lack of conversion of completely methylated, full-length OphMA by OphP *in vitro*. (**a**) ESI-MALDI-ToF analysis of purified OphMA (His_8-tag cleaved off by TEV protease after metal affinity purification from *E. coli* as described in[3]) coincubated with methyl donor substrate SAM and OphP (His_8SUMO*-tag cleaved off by TEV protease after metal affinity purification from *P. pastoris*) *in vitro*. Full spectra can be found in panels b, c and d. Dashed red lines indicate the various methylation states of OphMA. (**b**) Intact mass of full length (fl) OphMA expressed in *E. coli* for 2h at 16°C. (**c**) Intact mass of full length OphMA-2h incubated at 4 mg/ml with 5 mM SAM at room temperature in 50 mM Tris pH 8.0, 100 mM NaCl and 1 mM TCEP for 2h. (**d**) Intact mass of full length OphMA-2h incubated at 4 mg/ml with 5 mM SAM and 0.9 mg/ml OphP (10 mM) at room temperature in 50 mM Tris pH 8.0, 100 mM NaCl and 1 mM TCEP for 2h. The molecular mass of OphP (after TEV cleavage) is 84611 Da and the peak at 42303 Da corresponds to doubly charged OphP. In panels b, c, d the y axis is the % of the highest signal and the deconvoluted spectrum (thus mass not m/z is used) is shown. The raw data from experiments are shown in red above the spectra.

a

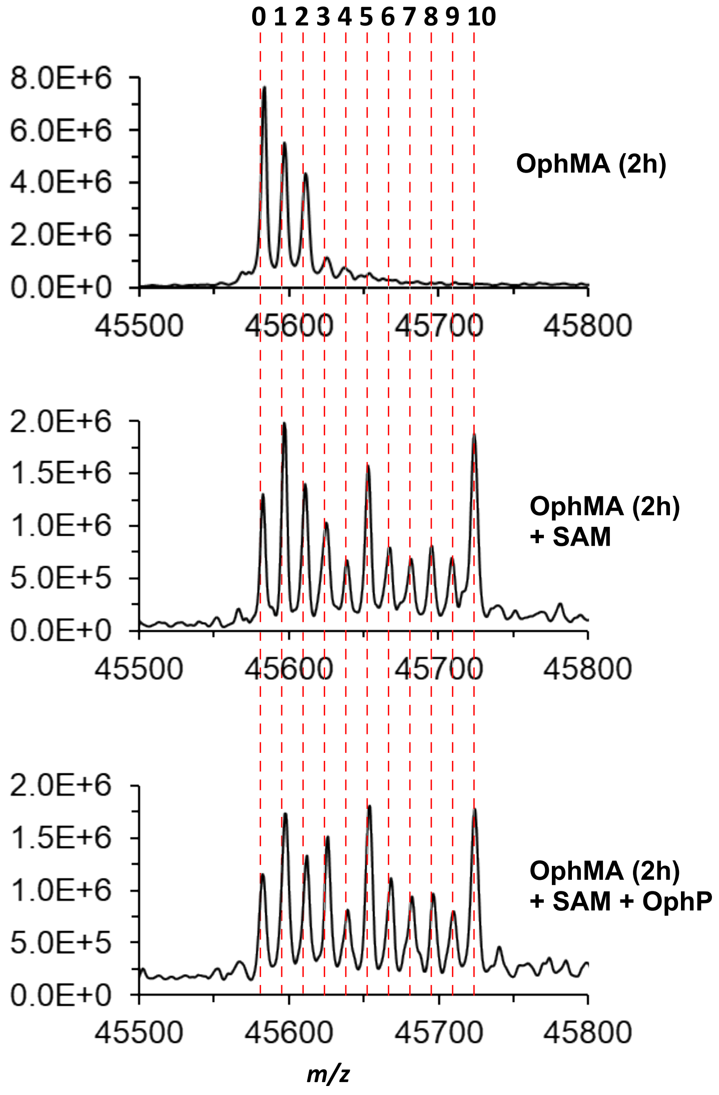

b

OphMA (2h)

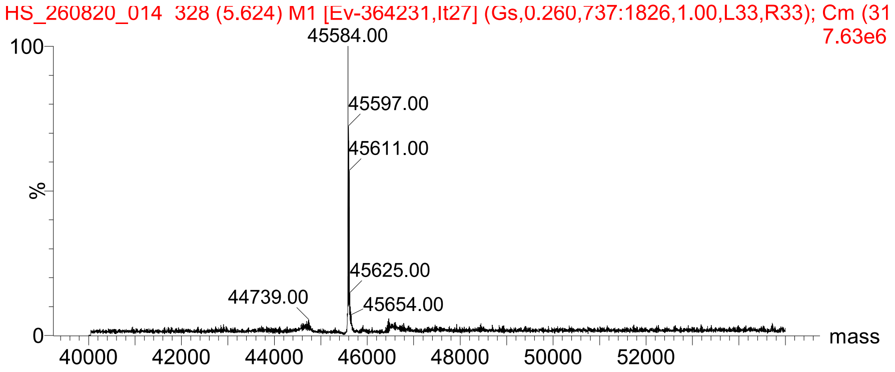

c

OphMA (2h) + SAM

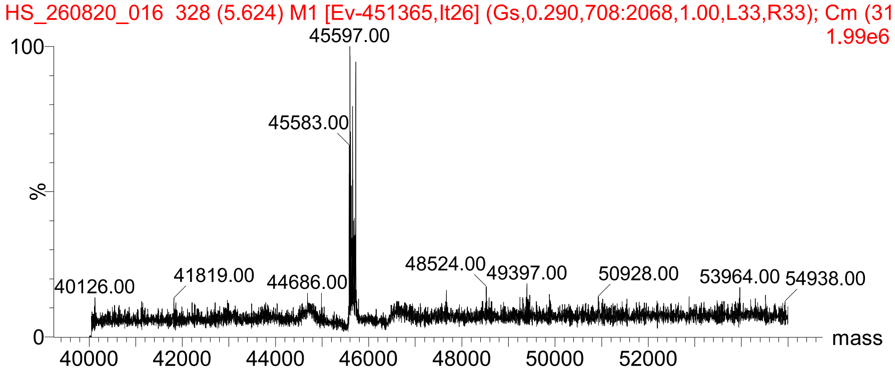

d

OphMA (2h) + SAM + OphP

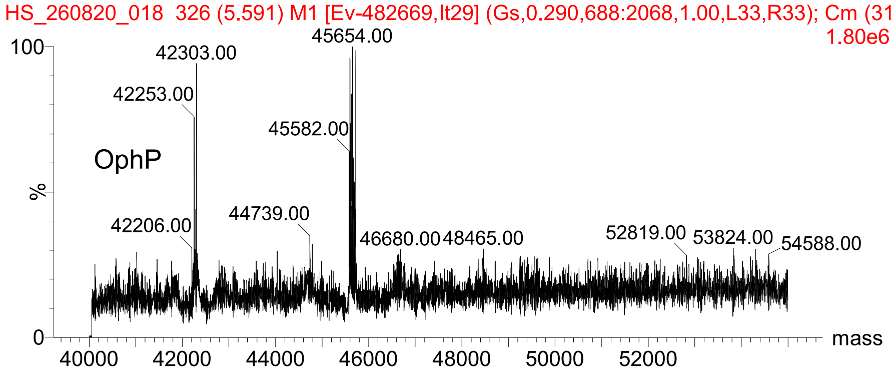

Figure S6. Analysis of complex formation between His$_8$OphMA and His$_8$SUMO*OphP(S580A). The two proteins were coexpressed in *Pichia pastoris* GS115 as described in[2,4] (using pPIC3.5K-His$_8$-SUMO*-TEVcs-OphP(S580A) instead of pPIC3.5K-His$_8$-OphP), purified from cell extracts using metal affinity chromatography and analyzed by size exclusion chromatography. (a) Size exclusion chromatography (HiLoad 16/600 Superdex 200 pg) of purified His$_8$OphMA and His$_8$SUMO*OphP(S580A). (b) SDS-PAGE of the corresponding fractions from the gel filtration. (c) Intact protein mass determination of His$_8$OphMA confirming integrity and methylation. The y-axis is the % of the highest signal and the deconvoluted spectrum (thus mass not m/z is used) is shown. The spectrum on the right is a zoom-in of the spectrum on the left. The raw data from experiments are shown in red above the spectra.

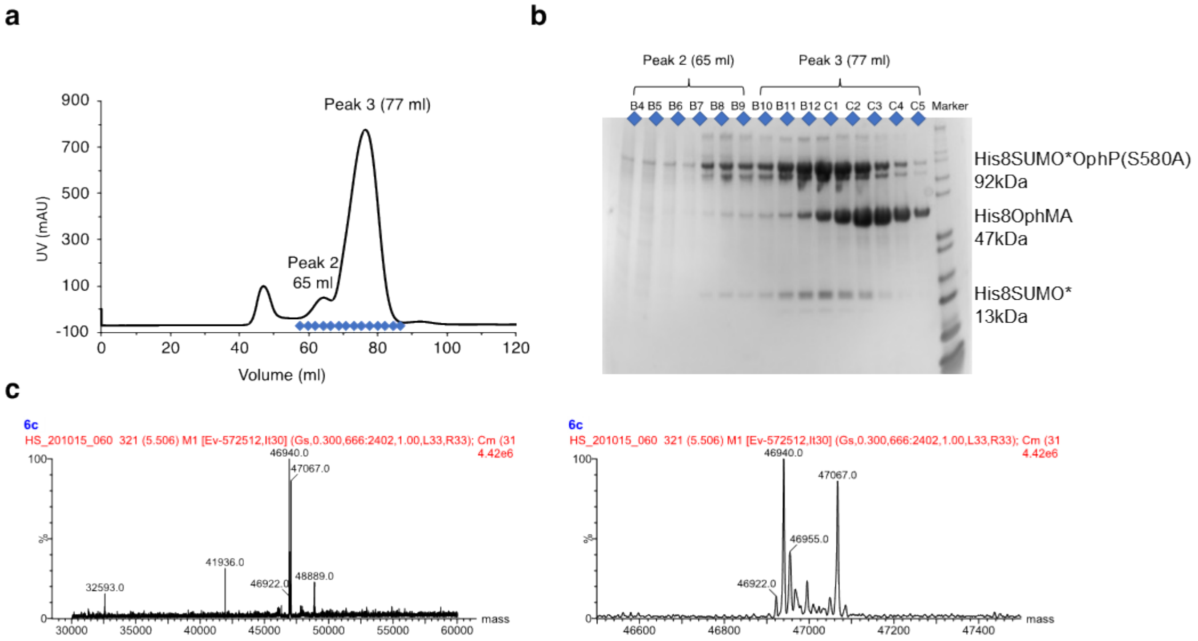

Figure S7. HPLC-MS analysis of OphP substrates. (a, b) Ion chromatograms of OphMA-derived, backbone N-methylated peptide substrates produced using TEV protease-cleavable OphMA-variants and TEV protease only (a) or a combination of TEV protease and GmPOPB (b). The number of methylated residues is indicated by green numbers and red dashed lines to facilitate a comparison. The different peptides have been aligned by extent of methylation rather than by absolute mass. The core peptide is underlined. Note that the follower peptide is different for the non-omphalotin peptides. In case of Oph-5, two different peptide batches with different ratios between 6- and 7-fold methylated species (1:1 and 1:10) were used as substrates. The one with a ratio of 1:10 is labeled 'Oph-5^{high}' for the rest of the Supplementary Information.

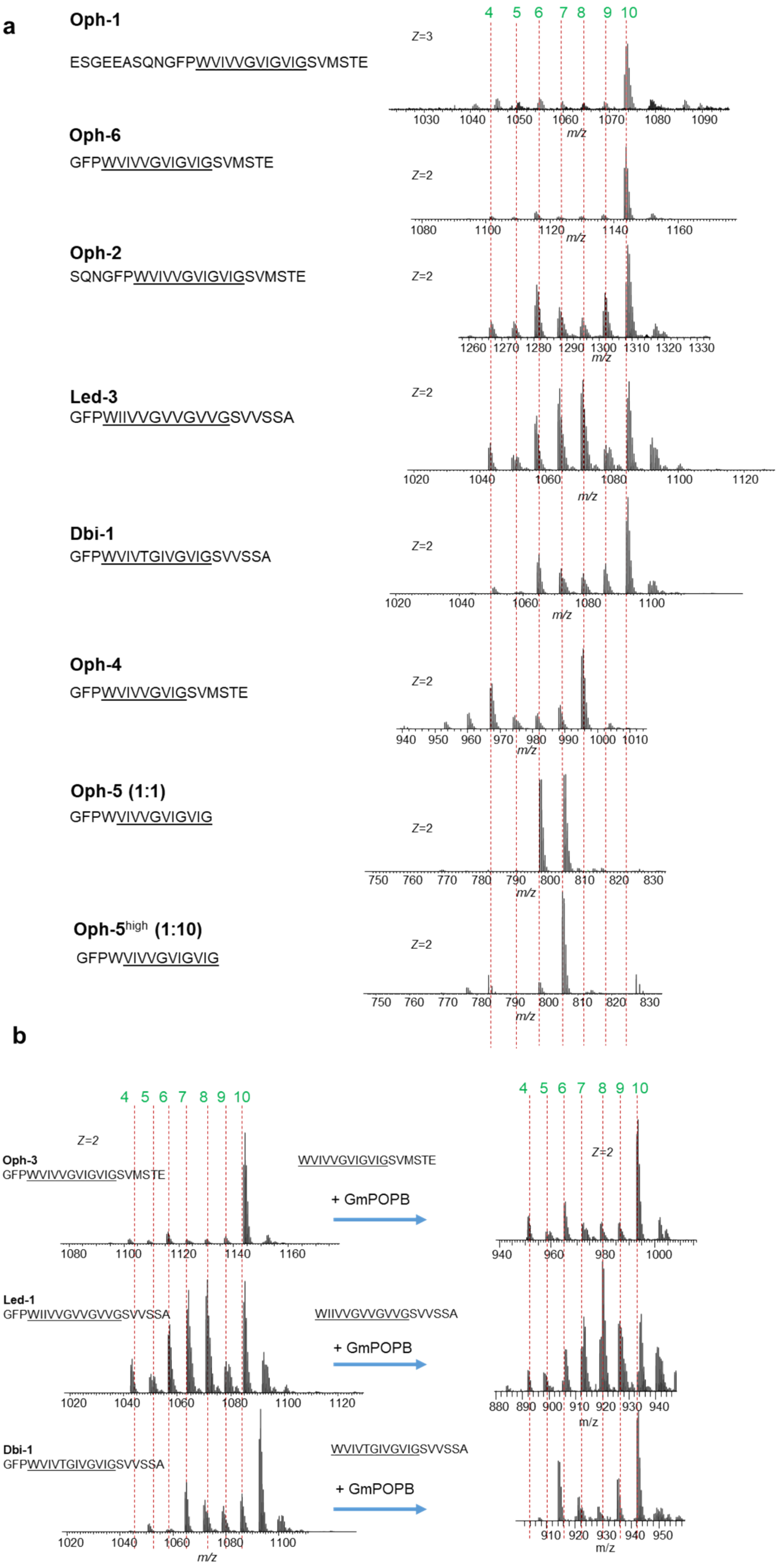
a
Oph-1
ESGEEASQNGFPWVIVVGVIGVIGSVMSTE
Oph-6
GFPWVIVVGVIGVIGSVMSTE
Oph-2
SQNGFPWVIVVGVIGVIGSVMSTE
Led-3
GFPWIIVVGVVGVVGSVVSSA
Dbi-1
GFPWVIVTGIVGVIGSVVSSA
Oph-4
GFPWVIVVGVIGSVMSTE
Oph-5 (1:1)
GFPWVIVVGVIGVIG
Oph-5high (1:10)
GFPWVIVVGVIGVIG
4 5 6 7 8 9 10
Z=3
Z=2
m/z
b
Oph-3
GFPWVIVVGVIGVIGSVMSTE
Led-1
GFPWIIVVGVVGVVGSVVSSA
Dbi-1
GFPWVIVTGIVGVIGSVVSSA
WVIVVGVIGVIGSVMSTE
WIIVVGVVGVVGSVVSSA
WVIVTGIVGVIGSVVSSA
+ GmPOPB

Figure S8. LC-MS/MS spectra of OphP peptide substrates and products. (**a-e**) Peptide substrates obtained by cleaving OphMA-variants with TEV protease: Oph-3, Led-1, Dbi-1, Oph-6, Oph-4, Oph-2 and Oph-5. (**f-l**) Linear peptide products obtained by incubation of the peptide substrates with OphP: linear 15mer and 12mer derived from Oph-3 (**f-g**), linear 12mer and 15mer derived from Led-1 (**h-i**), 15mer derived from Dbi-1 (**j**), linear 15mer and 18mer derived from Oph-2 (**k-l**). (**m-o**) Linear omphalotin A, lentinulin A and dendrothelin A intermediates. Methylated residues are highlighted in green color. b-ions and y-ions are shown in blue color. Mass tolerance between observed masses and the theoretical masses was set at 10 ppm. This difference is indicated in parentheses. Mass precision was set at 4 decimals. (**p**) Sequences of OphMA-derived OphP peptide substrates and products produced in this work, with α-N-methylated residues highlighted in orange. Note that methylation by OphMA proceeds from N- to C-terminus and that only the completely methylated forms of the peptides are displayed. The P1 residues recognized by OphP are underlined.

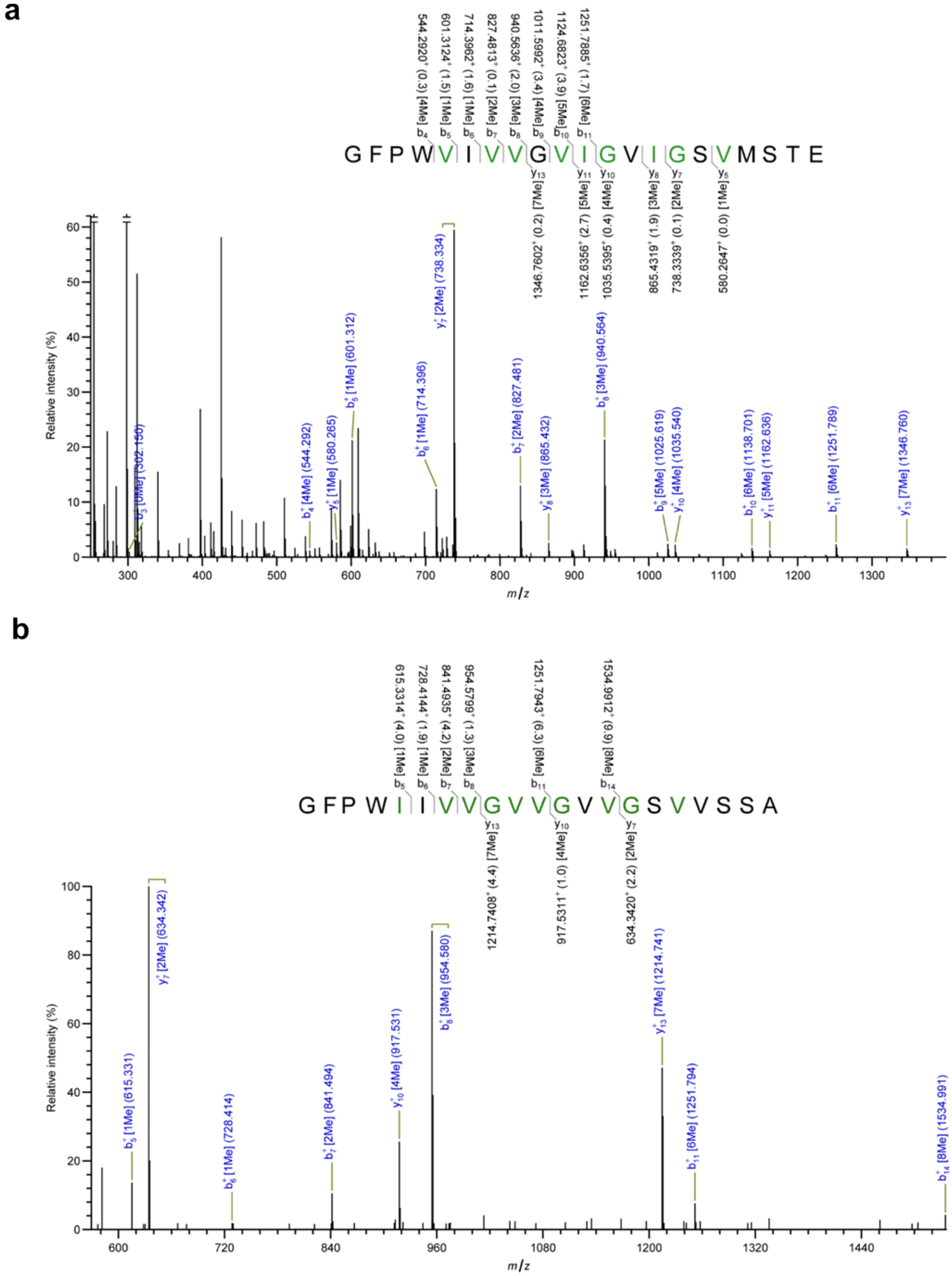
a
GFPWVIVVGVIGVIGSVMSTE
544.2920⁺ (0.3) [4Me] b4
601.3124⁺ (1.5) [1Me] b5
714.3962⁺ (1.6) [1Me] b6
827.4813⁺ (0.1) [2Me] b7
940.5636⁺ (2.0) [3Me] b8
1011.5992⁺ (3.4) [4Me] b9
1124.6823⁺ (3.9) [5Me] b10
1251.7885⁺ (1.7) [6Me] b11
y13 [7Me] (0.2) 1346.7602⁺
y11 [5Me] (2.7) 1162.6356⁺
y10 [4Me] (0.4) 1035.5395⁺
y8 [3Me] (1.9) 865.4319⁺
y7 [2Me] (0.1) 738.3339⁺
y5 [1Me] (0.0) 580.2647⁺
Relative intensity (%)
m/z
b3⁺ [0Me] (302.150)
b4⁺ [4Me] (544.292)
y5⁺ [1Me] (580.265)
b5⁺ [1Me] (601.312)
b6⁺ [1Me] (714.396)
y7⁺ [2Me] (738.334)
b7⁺ [2Me] (827.481)
y8⁺ [3Me] (865.432)
b8⁺ [3Me] (940.564)
b9⁺ [5Me] (1025.619)
y10⁺ [4Me] (1035.540)
b10⁺ [6Me] (1138.701)
y11⁺ [5Me] (1162.636)
b11⁺ [6Me] (1251.789)
y13⁺ [7Me] (1346.760)
b
GFPWIIVVGVVGVVGSVVSSA
615.3314⁺ (4.0) [1Me] b5
728.4144⁺ (1.9) [1Me] b6
841.4935⁺ (4.2) [2Me] b7
954.5799⁺ (1.3) [3Me] b8
1251.7943⁺ (6.3) [6Me] b11
1534.9912⁺ (6.9) [8Me] b14
y13 [7Me] (4.4) 1214.7408⁺
y10 [4Me] (1.0) 917.5311⁺
y7 [2Me] (2.2) 634.3420⁺
Relative intensity (%)
m/z
b5⁺ [1Me] (615.331)
y7⁺ [2Me] (634.342)
b6⁺ [1Me] (728.414)
b7⁺ [2Me] (841.494)
y10⁺ [4Me] (917.531)
b8⁺ [3Me] (954.580)
y13⁺ [7Me] (1214.741)
b11⁺ [6Me] (1251.794)
b14⁺ [8Me] (1534.991)

c

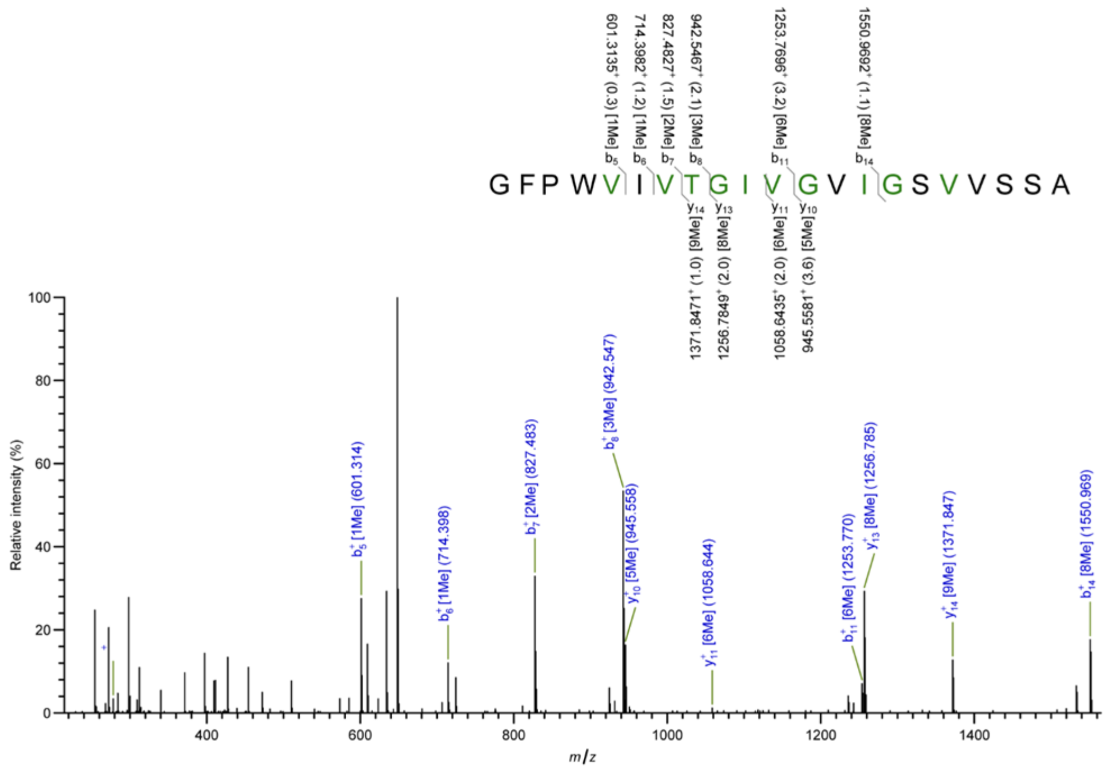
G F P W V I V T G I V G V I G S V V S S A
601.3135+ (0.3) [1Me] b5
714.3982+ (1.2) [1Me] b6
827.4827+ (1.5) [2Me] b7
942.5467+ (2.1) [3Me] b8
1253.7696+ (3.2) [6Me] b11
1550.9692+ (1.1) [8Me] b14
y14 1371.8471+ (1.0) [9Me]
y13 1256.7849+ (2.0) [8Me]
y11 1058.6435+ (2.0) [6Me]
y10 945.5581+ (3.6) [5Me]
b5+ [1Me] (601.314)
b6+ [1Me] (714.398)
b7+ [2Me] (827.483)
b8+ [3Me] (942.547)
y10+ [5Me] (945.558)
y11+ [6Me] (1058.644)
b11+ [6Me] (1253.770)
y13+ [8Me] (1256.785)
y14+ [9Me] (1371.847)
b14+ [8Me] (1550.969)
Relative intensity (%)
m/z
0
20
40
60
80
100
400
600
800
1000
1200
1400

d

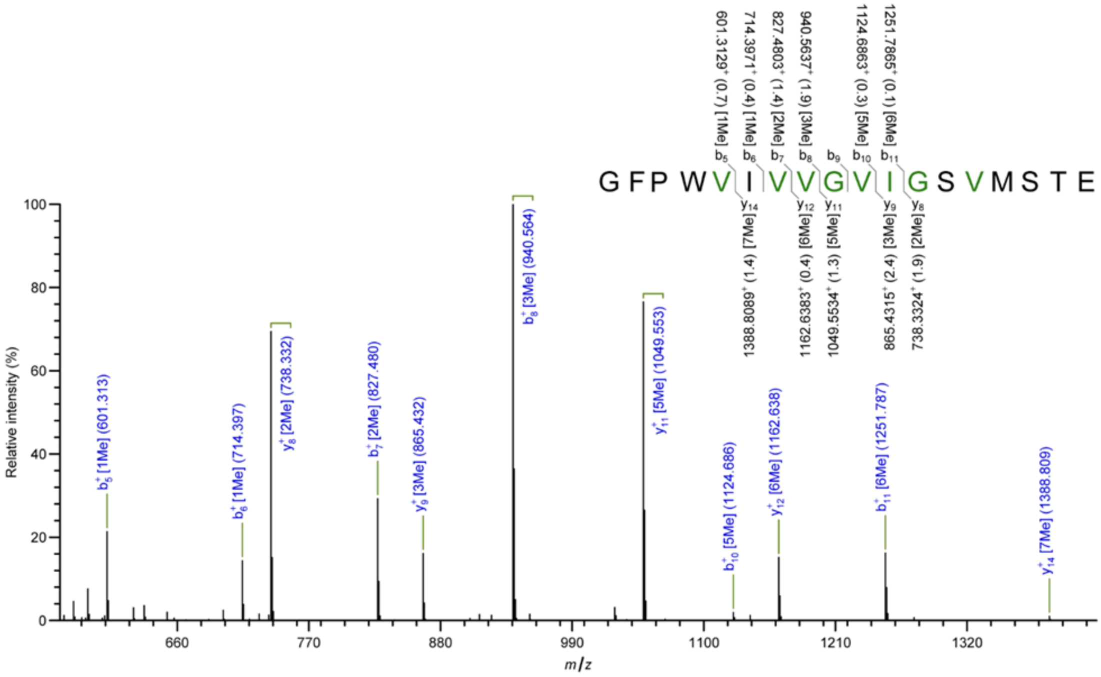
G F P W V I V V G V I G S V M S T E
601.3129+ (0.7) [1Me] b5
714.3971+ (0.4) [1Me] b6
827.4803+ (1.4) [2Me] b7
940.5637+ (1.9) [3Me] b8
b9
1124.6863+ (0.3) [5Me] b10
1251.7865+ (0.1) [6Me] b11
y14 1388.8089+ (1.4) [7Me]
y12 1162.6383+ (0.4) [6Me]
y11 1049.5534+ (1.3) [5Me]
y9 865.4315+ (2.4) [3Me]
y8 738.3324+ (1.9) [2Me]
b5+ [1Me] (601.313)
b6+ [1Me] (714.397)
y8+ [2Me] (738.332)
b7+ [2Me] (827.480)
y9+ [3Me] (865.432)
b8+ [3Me] (940.564)
y11+ [5Me] (1049.553)
b10+ [5Me] (1124.686)
y12+ [6Me] (1162.638)
b11+ [6Me] (1251.787)
y14+ [7Me] (1388.809)
Relative intensity (%)
m/z
0
20
40
60
80
100
660
770
880
990
1100
1210
1320

e

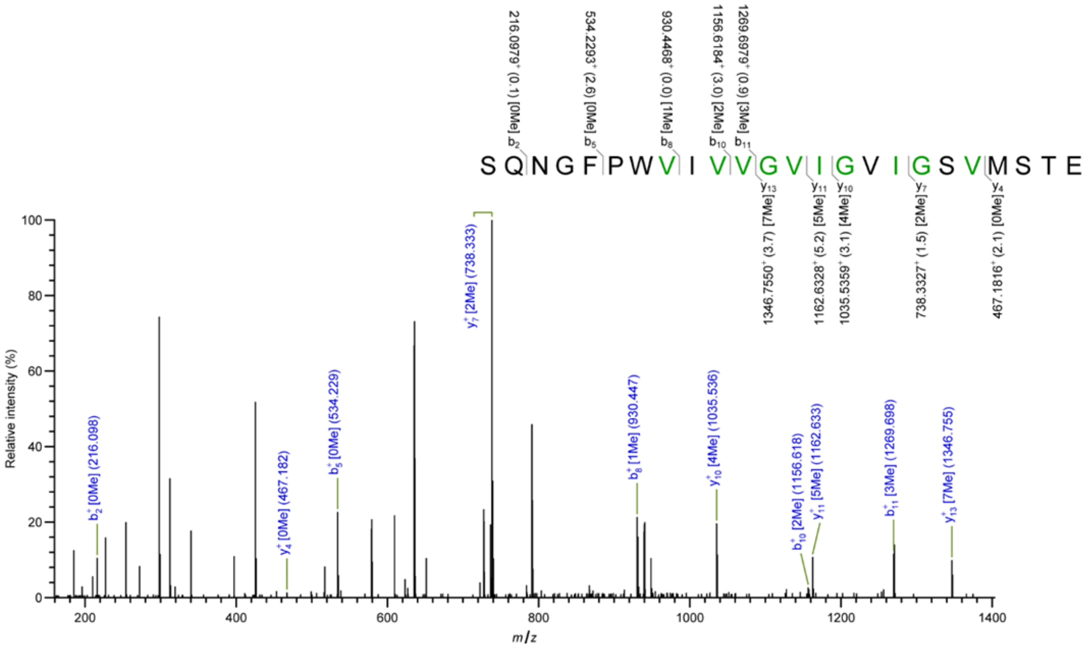

SQNGFPWVIVVGVIGVIGSVMSTE
Relative intensity (%)
m/z
y7+ [2Me] (738.333)
b2+ [0Me] (216.098)
y4+ [0Me] (467.182)
b5+ [0Me] (534.229)
b8+ [1Me] (930.447)
y10+ [4Me] (1035.536)
b10+ [2Me] (1156.618)
y11+ [5Me] (1162.633)
b11+ [3Me] (1269.698)
y13+ [7Me] (1346.755)

f

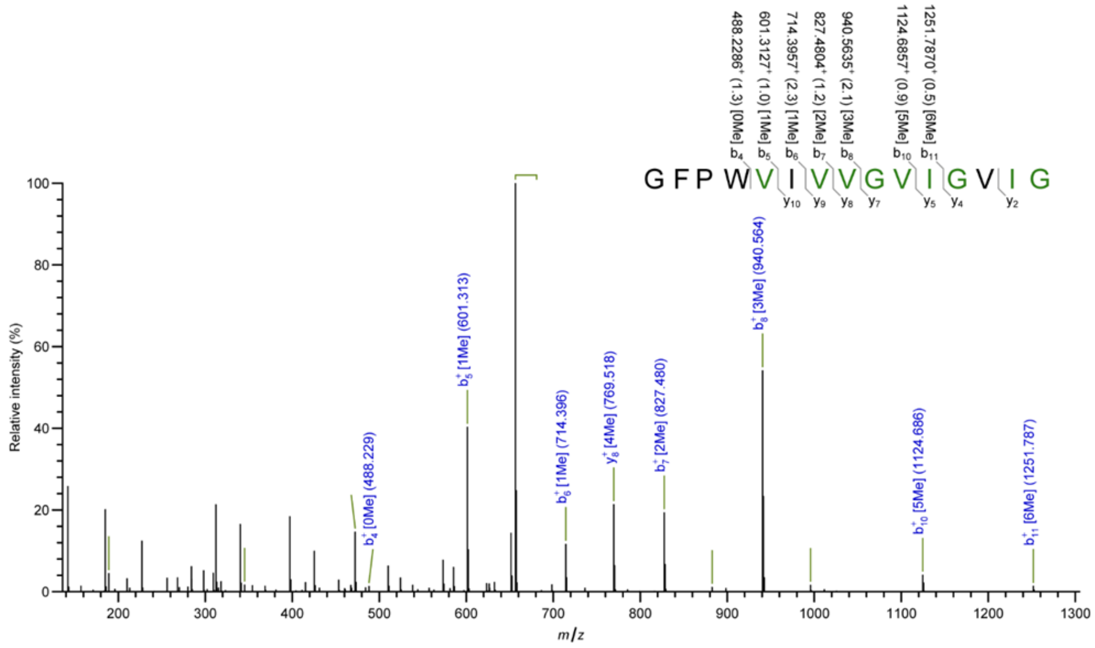

g

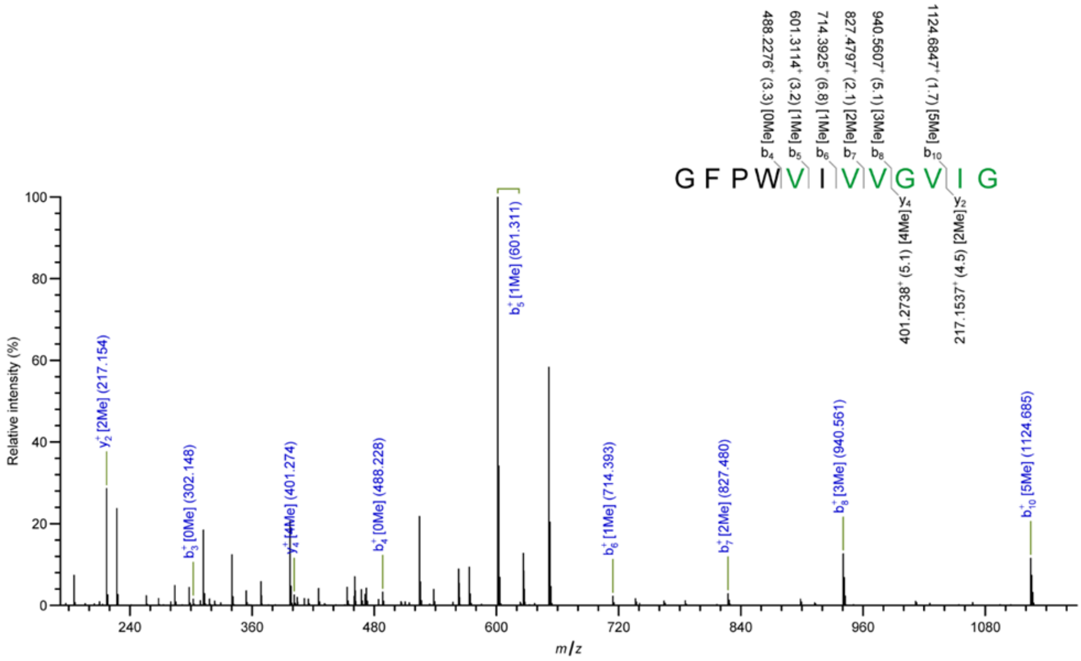

h

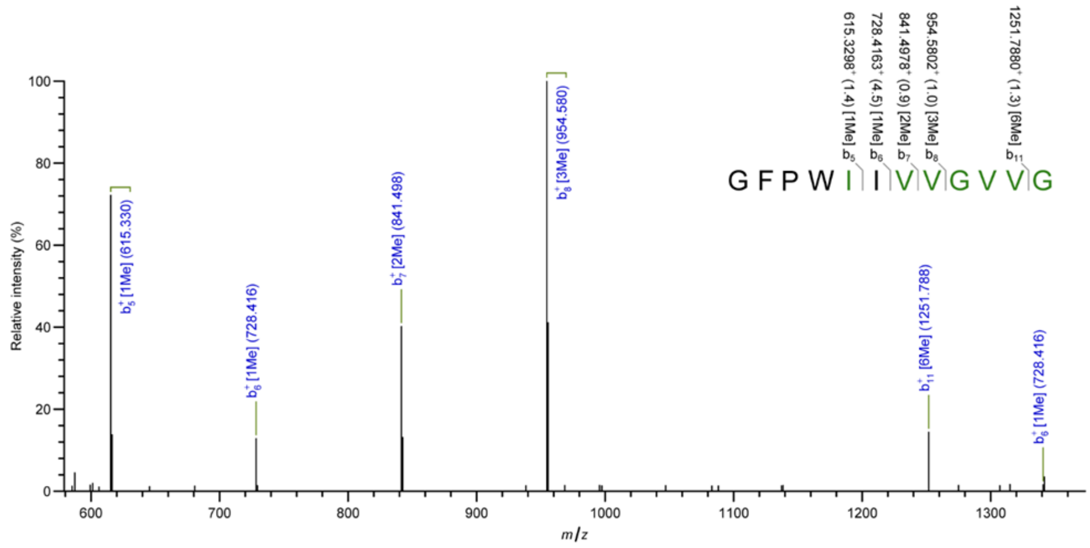
GFPWIIVVGVVG
615.3298+ (1.4) [1Me]
728.4163+ (4.5) [1Me]
841.4978+ (0.9) [2Me]
954.5802+ (1.0) [3Me]
1251.7880+ (1.3) [6Me]
Relative intensity (%)
m/z
b5+ [1Me] (615.330)
b6+ [1Me] (728.416)
b7+ [2Me] (841.498)
b8+ [3Me] (954.580)
b11+ [6Me] (1251.788)

i

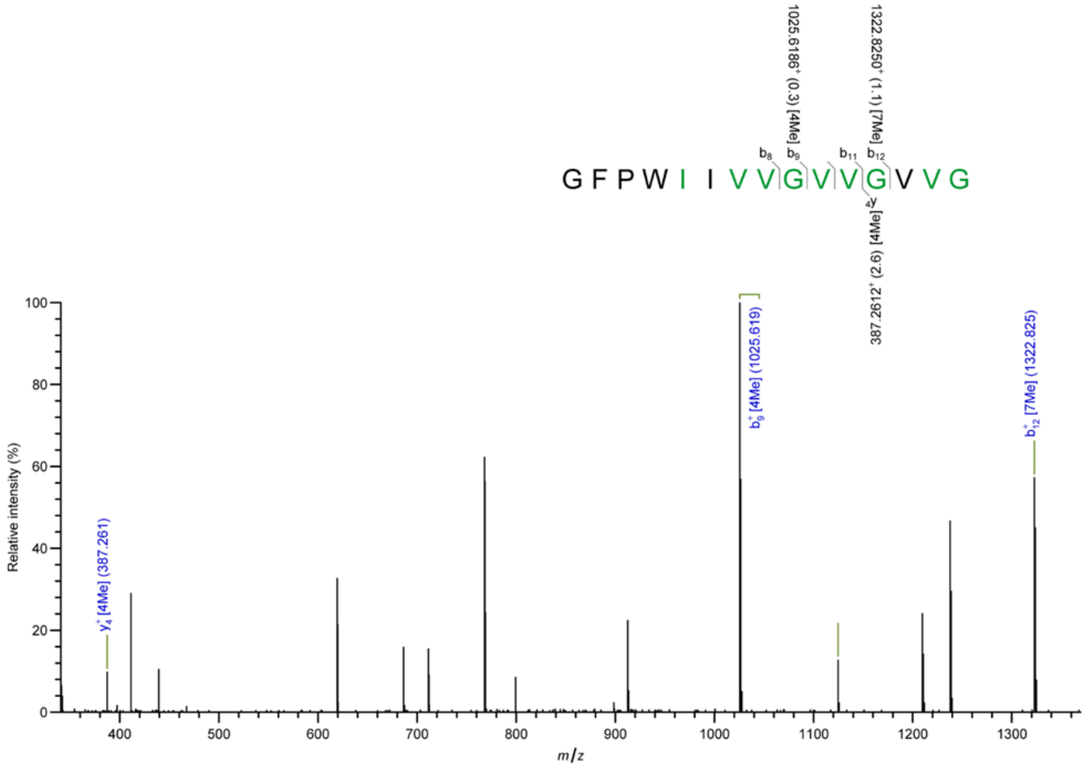
GFPWIIVVGVVGVVG
1025.6186+ (0.3) [4Me]
1322.8250+ (1.1) [7Me]
387.2612+ (2.6) [4Me]
Relative intensity (%)
m/z
y4+ [4Me] (387.261)
b9+ [4Me] (1025.619)
b12+ [7Me] (1322.825)

j

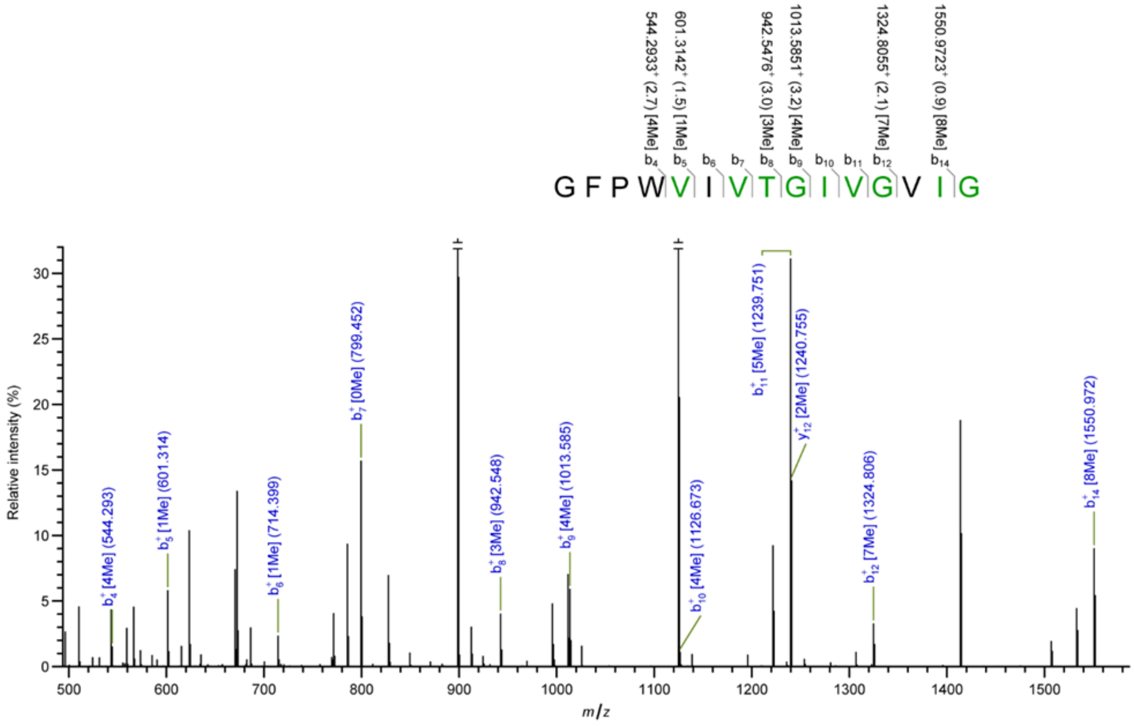

G F P W V I V T G I V G V I G
544.2933+ (2.7) [4Me]
601.3142+ (1.5) [1Me]
942.5476+ (3.0) [3Me]
1013.5851+ (3.2) [4Me]
1324.8055+ (2.1) [7Me]
1550.9723+ (0.9) [8Me]
Relative intensity (%)
m/z
b4+ [4Me] (544.293)
b5+ [1Me] (601.314)
b6+ [1Me] (714.399)
b7+ [0Me] (799.452)
b8+ [3Me] (942.548)
b9+ [4Me] (1013.585)
b10+ [4Me] (1126.673)
b11+ [5Me] (1239.751)
y12+ [2Me] (1240.755)
b12+ [7Me] (1324.806)
b14+ [8Me] (1550.972)

k

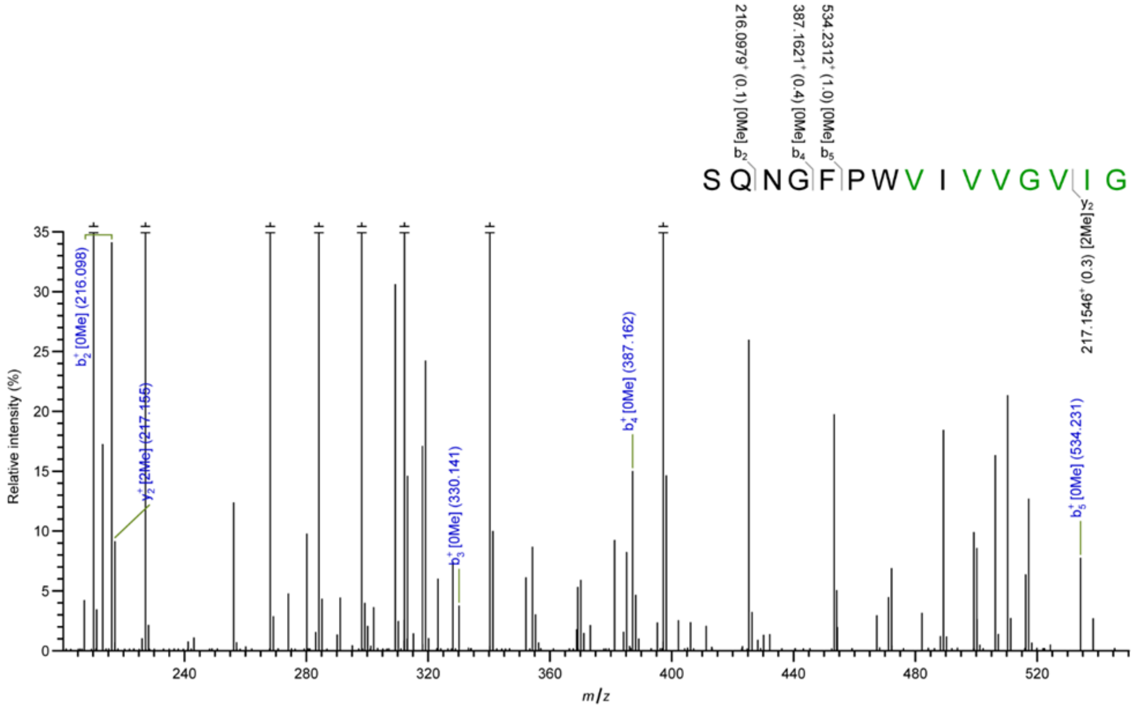

S Q N G F P W V I V V G V I G
216.0979+ (0.1) [0Me]
387.1621+ (0.4) [0Me]
534.2312+ (1.0) [0Me]
217.1546+ (0.3) [2Me]
Relative intensity (%)
m/z
b2+ [0Me] (216.098)
y2+ [2Me] (217.155)
b3+ [0Me] (330.141)
b4+ [0Me] (387.162)
b5+ [0Me] (534.231)

l

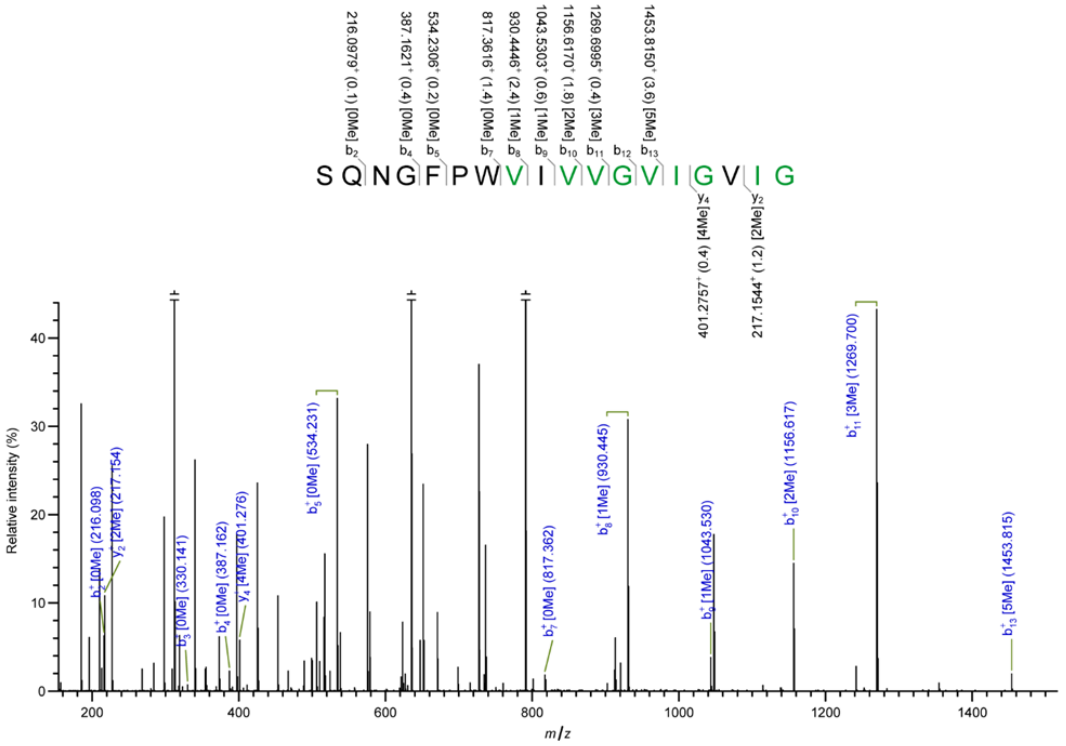

m

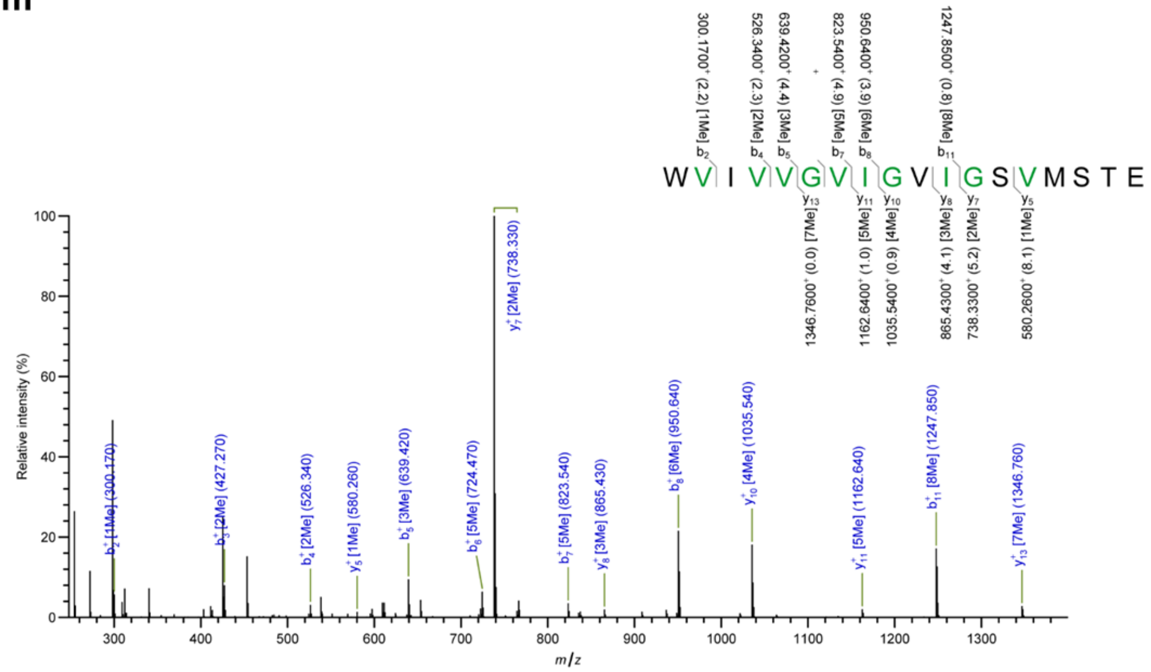

n

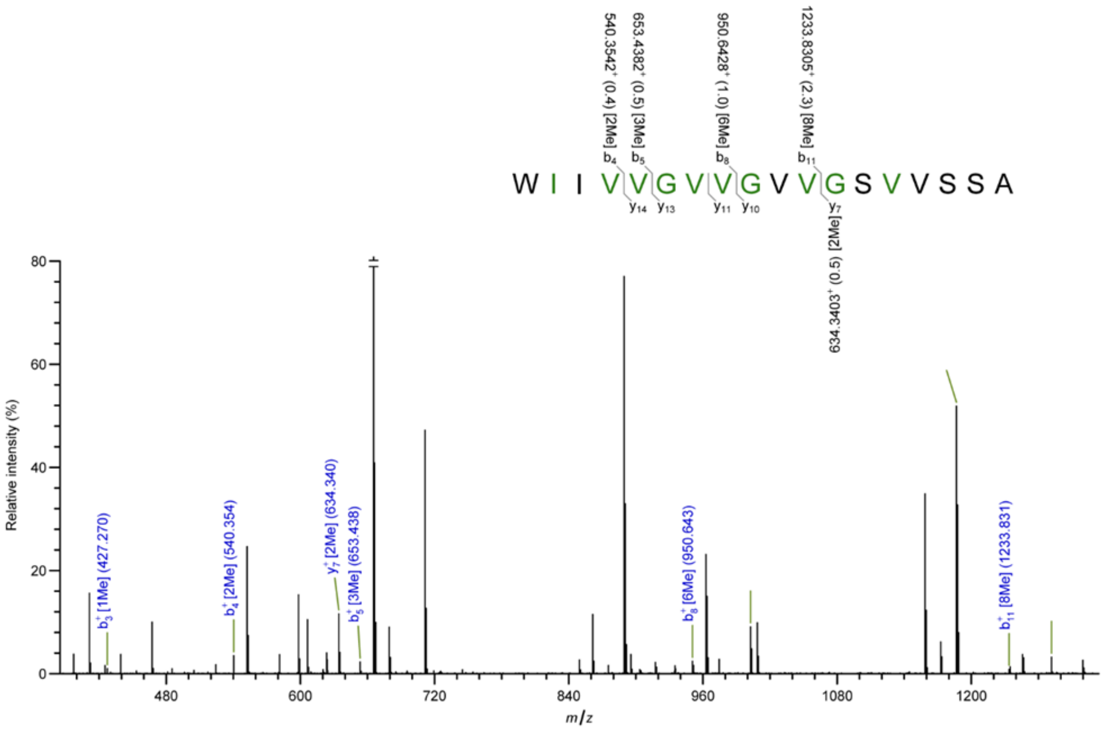
W I I V V G V V G V V G S V V S S A
540.3542+ (0.4) [2Me] b4
653.4382+ (0.5) [3Me] b5
950.6428+ (1.0) [6Me] b8
1233.8305+ (2.3) [8Me] b11
634.3403+ (0.5) [2Me] y7
b3+ [1Me] (427.270)
b4+ [2Me] (540.354)
y7+ [2Me] (634.340)
b5+ [3Me] (653.438)
b8+ [6Me] (950.643)
b11+ [8Me] (1233.831)
Relative intensity (%)
m/z

o

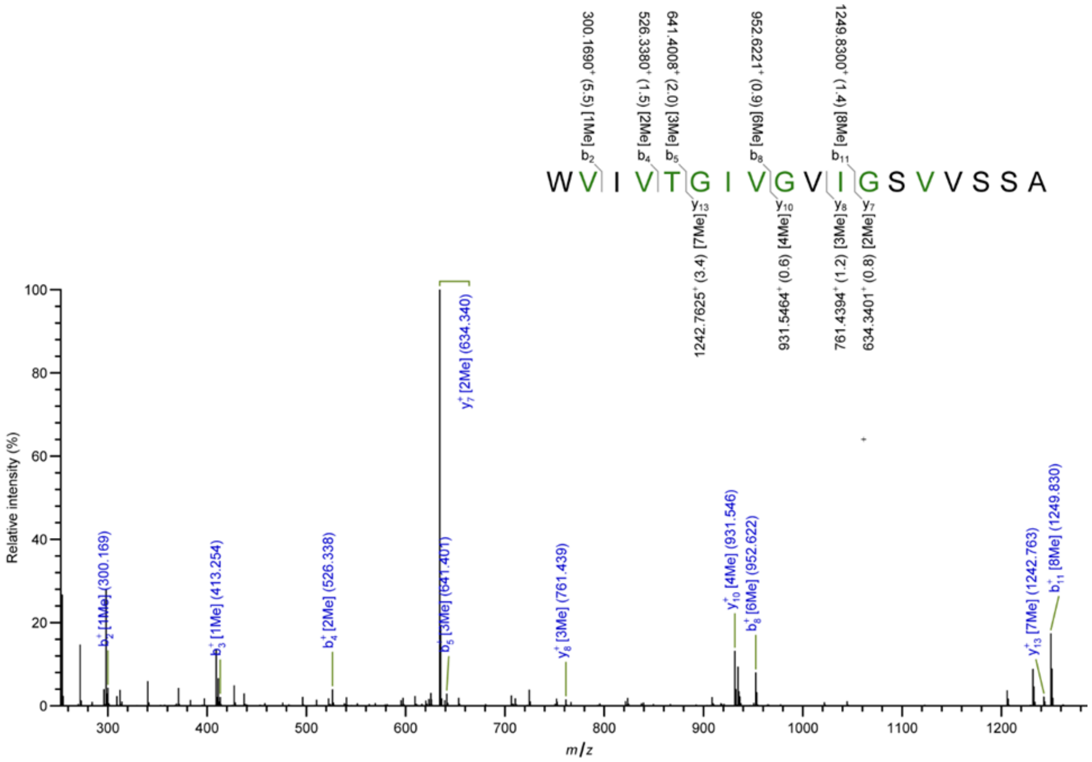
W V I V T G I V G V I G S V V S S A
300.1690+ (5.5) [1Me] b2
526.3380+ (1.5) [2Me] b4
641.4008+ (2.0) [3Me] b5
952.6221+ (0.9) [6Me] b8
1249.8300+ (1.4) [8Me] b11
1242.7625+ (3.4) [7Me] y13
931.5464+ (0.6) [4Me] y10
761.4394+ (1.2) [3Me] y8
634.3401+ (0.8) [2Me] y7
y7+ [2Me] (634.340)
b2+ [1Me] (300.169)
b3+ [1Me] (413.254)
b4+ [2Me] (526.338)
b5+ [3Me] (641.401)
y8+ [3Me] (761.439)
y10+ [4Me] (931.546)
b8+ [6Me] (952.622)
y13+ [7Me] (1242.763)
b11+ [8Me] (1249.830)
Relative intensity (%)
m/z

p

**OphP substrate**	**Protein/peptide sequence**	**Linear OphP product(s)**	
**OphMA (417 aa)**	…ESGEEASQNGFPWVIVVGVIGVIGSVMSTE	None	None
**Oph-1**	ESGEEASQNGFPWVIVVGVIGVIGSVMSTE	None	None
**Oph-2**	SQNGFPWVIVVGVIGVIGSVMSTE	SQNGFPWVIVVGVIGVIG<br>SQNGFPWVIVVGVIG	cyclo-SQNGFPWVIVVGVIGVIG<br>cyclo-SQNGFPWVIVVGVIG
**Oph-3**	GFPWVIVVGVIGVIGSVMSTE	GFPWVIVVGVIGVIG<br>GFPWVIVVGVIG	cyclo-GFPWVIVVGVIGVIG<br>cyclo-GFPWVIVVGVIG
**Oph-4**	GFPWVIVVGVIGSVMSTE	GFPWVIVVGVIG	cyclo-GFPWVIVVGVIG
**Oph-5**	GFPWVIVVGVIGVIG	GFPWVIVVGVIG	cyclo-GFPWVIVVGVIG
**Oph-6**	WVIVVGVIGVIGSVMSTE	WVIVVGVIGVIG<br>WVIVVGVIG	cyclo-WVIVVGVIGVIG<br>(omphalotin A)
**Oph-7**	WVIVVGVIGSVMSTE	WVIVVGVIG	None
**Oph-8**	WVIVVGVIGVIG	WVIVVGVIG	None
**Led-1**	GFPWIIVVGVVGVVGSVVSSA	GFPWIIVVGVVGVVG<br>GFPWIIVVGVVG	cyclo-GFPWIIVVGVVGVVG<br>cyclo-GFPWIIVVGVVG
**Dbi-1**	GFPWVIVTGIVGVIGSVVSSA	GFPWVIVTGIVGVIG<br>GFPWVIVTGIVG	cyclo-GFPWVIVTGIVGVIG<br>cyclo-GFPWVIVTGIVG
**Led-2**	WIIVVGVVGVVGSVVSSA	WIIVVGVVGVVG<br>WIIVVGVVG	cyclo-WIIVVGVVGVVG<br>(lentinulin A)
**Dbi-2**	WVIVTGIVGVIGSVVSSA	WVIVTGIVGVIG<br>WVIVTGIVG	cyclo-WVIVTGIVGVIG<br>(dendrothelin A)

Figure S9. Activities of OphP, LedP, GmPOPB and ProAlanase towards Oph-5 (1:1 batch, see Figure S7a), Oph-6, Led-2 and Dbi-2 . (a) GmPOPB compared to OphP and LedP activities. (**b**) ProAlanase activity. The enzymatic activity was assessed by determining the UV absorbance at 280 nm. (**c**) MS-analysis of omphalotin A and its orthologues lentinulin A and dendrothelin A produced from Oph-6, Led-2 and Dbi-2 using OphP. The MS chromatograms show masses of both protonated cyclic peptides and corresponding sodium and ammonium adducts.

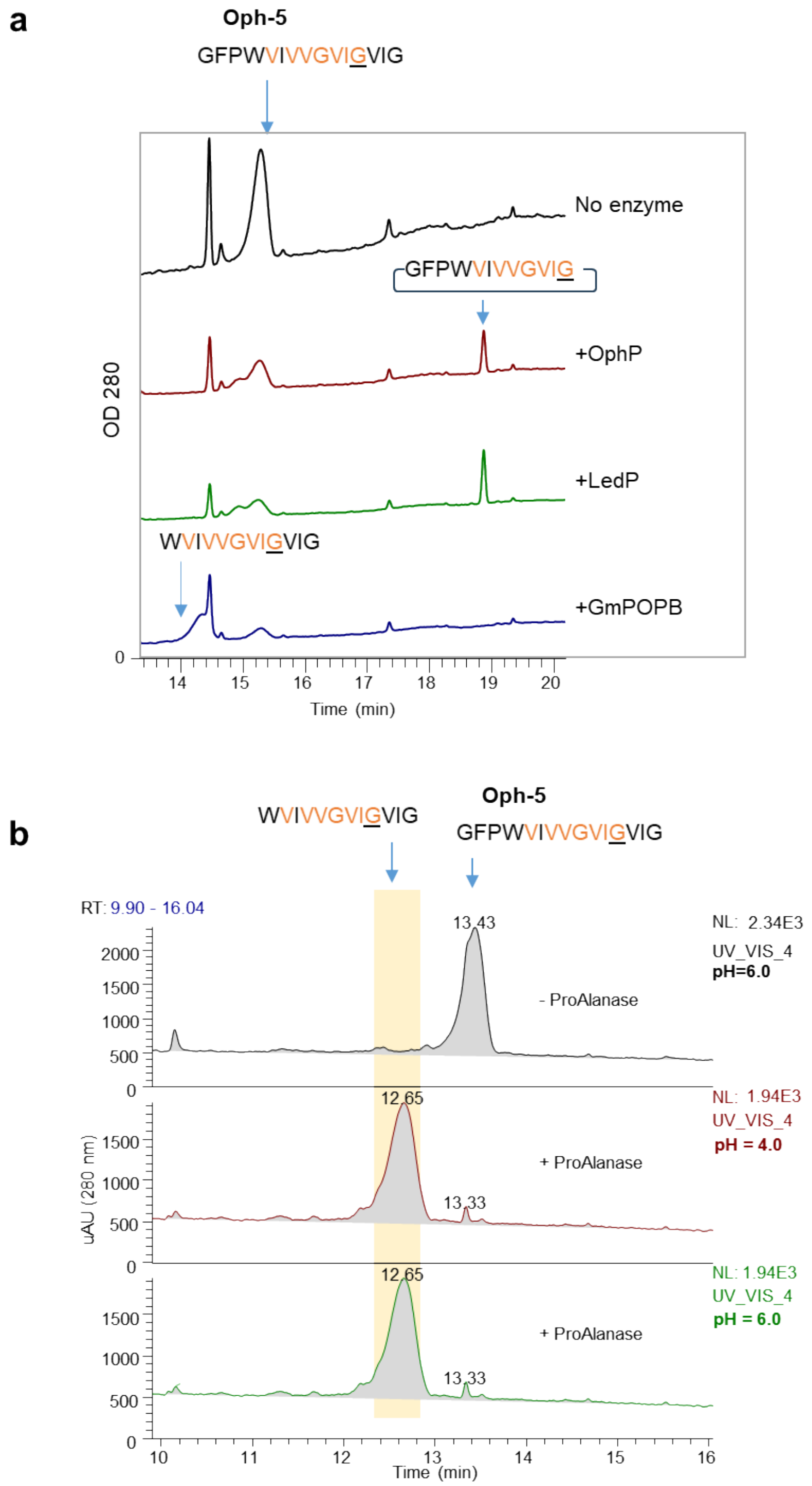

c

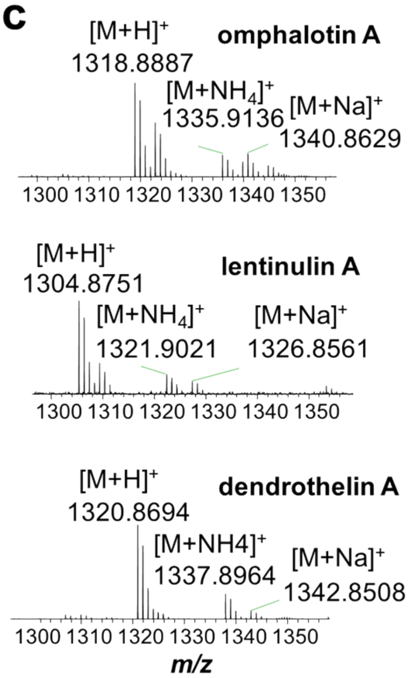

[M+H]+
1318.8887
omphalotin A
[M+NH4]+
1335.9136
[M+Na]+
1340.8629
1300 1310 1320 1330 1340 1350
[M+H]+
1304.8751
lentinulin A
[M+NH4]+
1321.9021
[M+Na]+
1326.8561
1300 1310 1320 1330 1340 1350
[M+H]+
1320.8694
dendrothelin A
[M+NH4]+
1337.8964
[M+Na]+
1342.8508
1300 1310 1320 1330 1340 1350
m/z

Figure S10. HPLC-MS of macrocyclic OphP peptide products. Extracted ion chromatograms (EICs) of peptide products derived from Led-1 and Dbi-1 by the action of OphP. Methylated residues are highlighted in green. Macrocyclization is indicated by a bracket below the peptide sequence.

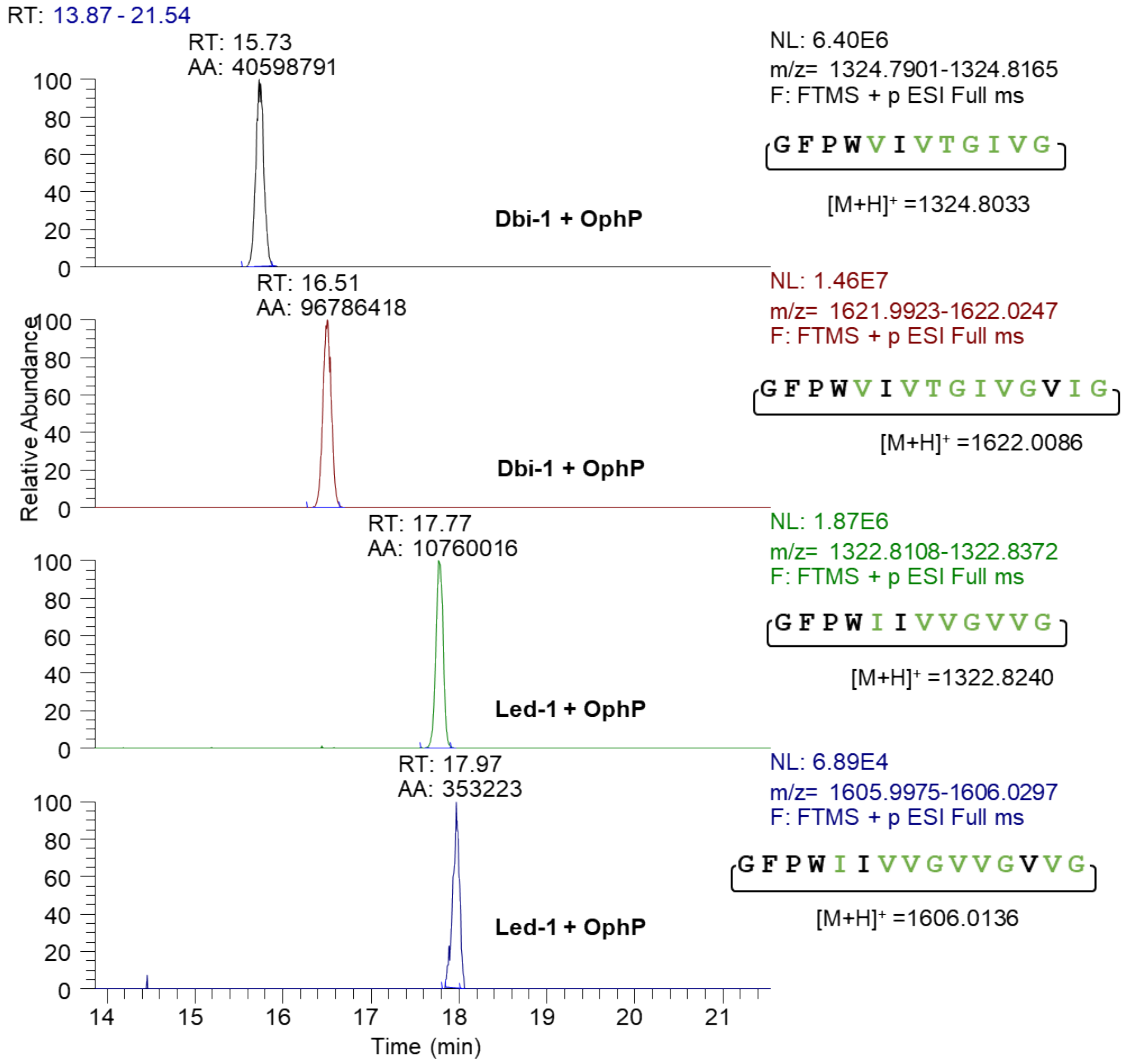

Figure S11. Preference of OphP for highly methylated peptide substrates by variation of enzyme:substrate ratio. The reaction was conducted in the standard buffer [50 mM HEPES pH 7.0, 10 mM DTT] at 30°C, overnight**.** NOTE: The batch of Oph-5 used for these experiments was the same as used for Figure 3a (1:1 batch, see Figure S4a). (**a**) RP-HPLC UV absorption profile of 100 µM of Oph-5 with varying concentrations of OphP (0, 1, 2, 5, 10, 20 µM). Linear products resulting from the peptidase activity of OphP can be detected but overlapped with the starting material. 6-fold methylated cyclo(GFPW^{me}VI^{me}V^{me}V^{me}G^{me}V^{me}IG) can only be detected when high concentrations of OphP are used. Dashed lines show the retention times of the various peptide species. (**b**) Mass spectrometric analysis showing ion chromatograms of the consumption of Oph-5 mixture (100 µM) when subjected to different concentrations of OphP. The 7-fold methylated Oph-5 is depleted at high concentrations of OphP (10 and 20 µM).

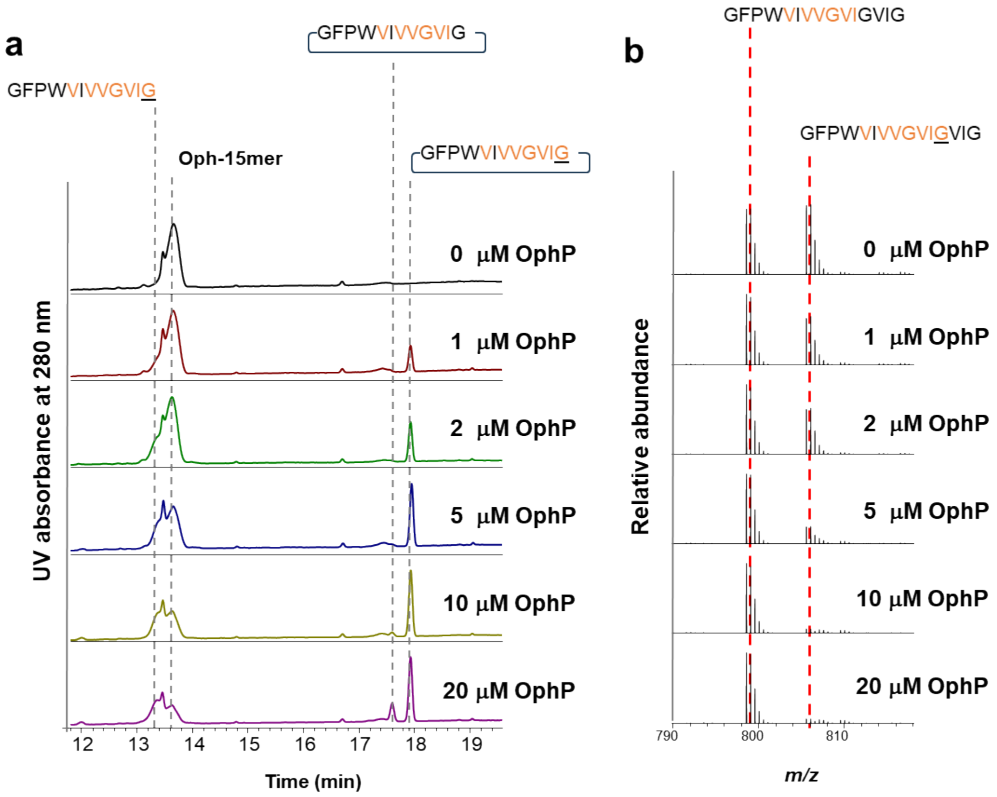

Figure S12. Preference of OphP for highly methylated peptide substrates by a time course of substrate depletion and product formation. NOTE: The batch of Oph-5^{high} used for these experiments was the same as used for Figure 3bcd (1:10 batch, see Figure S4a). (**a,b**) EIC peak area of Oph-5^{high} substrate and product species of 30 min *in vitro* reaction with 10µM OphP and Oph-5^{high} substrate (mixture of 6Me and 7Me) in HEPES buffer at pH 7.0. (**a**) Comparison of 6- and 7-fold methylated substrate species at 100µM total substrate concentration without addition of OphP. (**b**) Comparison of 6- and 7-fold methylated product species at equimolar (90µM) substrate concentration and 10µM OphP concentration. (**c-f**) Time course of *in vitro* reaction between 10µM OphP and 100µM total Oph-5^{high} substrate (mixture of 6Me and 7Me species) in HEPES buffer at pH 7.0. EIC peak areas of 6- and 7-fold methylated peptide substrate (**c**) and product (**d**) species were measured at 8 time points over 4 hours. Left panels in (**c, d**) show the absolute EIC peak areas and the right panels the ratio between the 7- to 6-fold methylated peptide species over the 7 time points. A batch of Oph-5 with a higher ratio of 7Me to 6Me species was used in this experiment (see Fig. S5 for a comparison between the batches). (**e, f**) EICs of the 7-fold (**e**) and 6-fold (**f**) methylated substrates and respective cyclic products over the time course (z-axis). The relative abundance is set to 100 for the respective substrate at each time point.

a

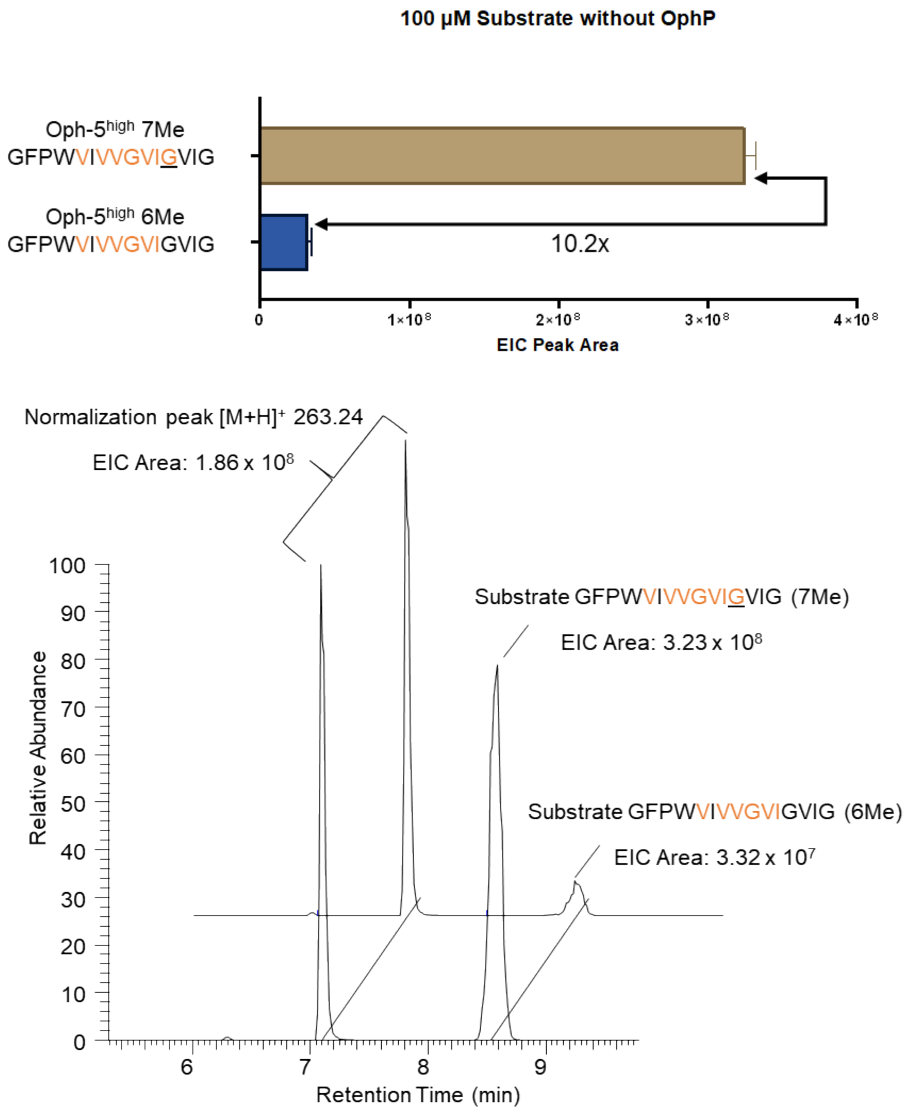
100 µM Substrate without OphP
Oph-5high 7Me
GFPWVIVVGVIGVIG
Oph-5high 6Me
GFPWVIVVGVIGVIG
10.2x
0
1×10⁸
2×10⁸
3×10⁸
4×10⁸
EIC Peak Area
Normalization peak [M+H]+ 263.24
EIC Area: 1.86 x 10⁸
Substrate GFPWVIVVGVIGVIG (7Me)
EIC Area: 3.23 x 10⁸
Substrate GFPWVIVVGVIGVIG (6Me)
EIC Area: 3.32 x 10⁷
Relative Abundance
100
90
80
70
60
50
40
30
20
10
0
6
7
8
9
Retention Time (min)

b

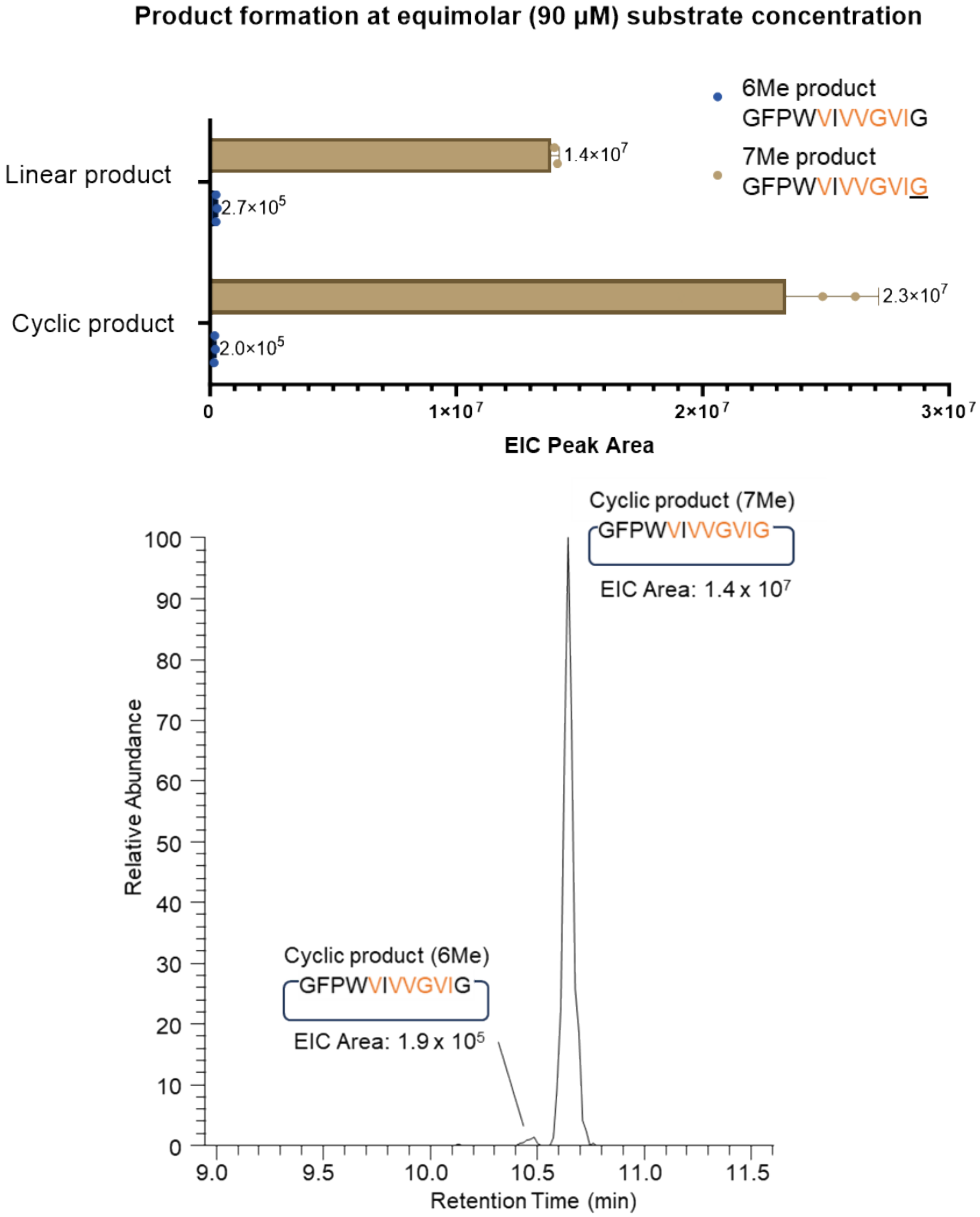
Product formation at equimolar (90 µM) substrate concentration
6Me product
GFPWVIVVGVIG
7Me product
GFPWVIVVGVIG
Linear product
Cyclic product
1.4×10^7
2.7×10^5
2.3×10^7
2.0×10^5
0
1×10^7
2×10^7
3×10^7
EIC Peak Area
Cyclic product (7Me)
GFPWVIVVGVIG
EIC Area: 1.4 x 10^7
Cyclic product (6Me)
GFPWVIVVGVIG
EIC Area: 1.9 x 10^5
Relative Abundance
100
90
80
70
60
50
40
30
20
10
0
9.0
9.5
10.0
10.5
11.0
11.5
Retention Time (min)

c

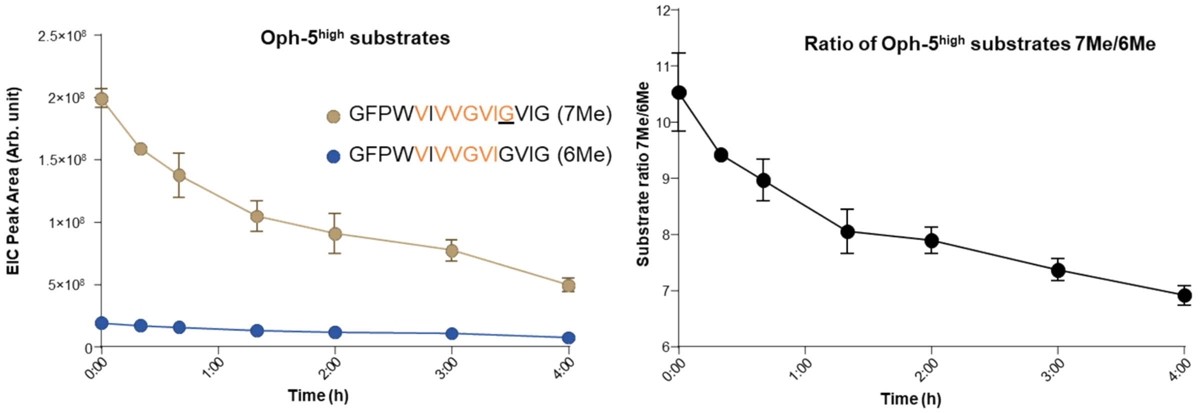
Oph-5high substrates
GFPWVIVVGVIGVIG (7Me)
GFPWVIVVGVIGVIG (6Me)
EIC Peak Area (Arb. unit)
2.5×10^8
2×10^8
1.5×10^8
1×10^8
5×10^8
0
0:00
1:00
2:00
3:00
4:00
Time (h)
Ratio of Oph-5high substrates 7Me/6Me
Substrate ratio 7Me/6Me
12
11
10
9
8
7
6
Time (h)

d

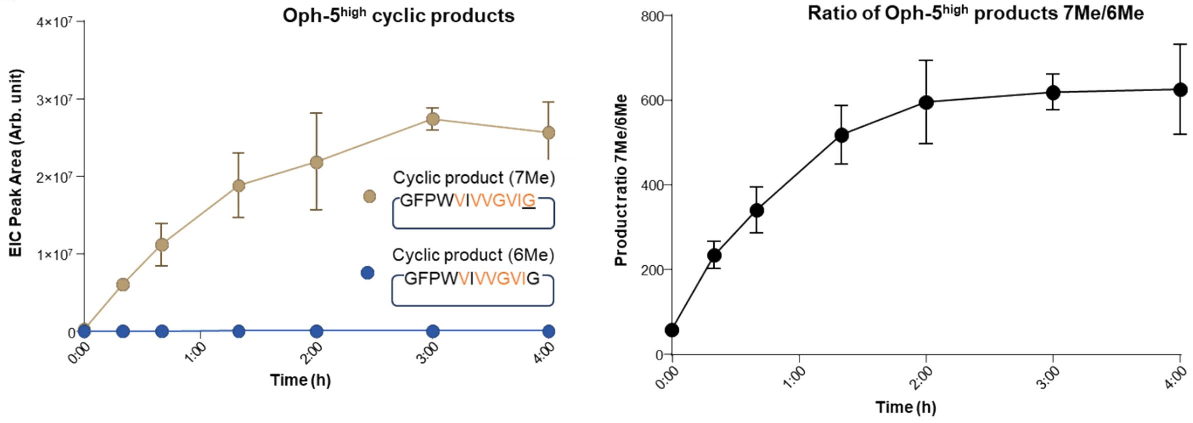
Oph-5high cyclic products
Cyclic product (7Me)
GFPWVIVVGVIG
Cyclic product (6Me)
GFPWVIVVGVIG
EIC Peak Area (Arb. unit)
4×10^7
3×10^7
2×10^7
1×10^7
0
0:00
1:00
2:00
3:00
4:00
Time (h)
Ratio of Oph-5high products 7Me/6Me
Product ratio 7Me/6Me
800
600
400
200
0
Time (h)

e

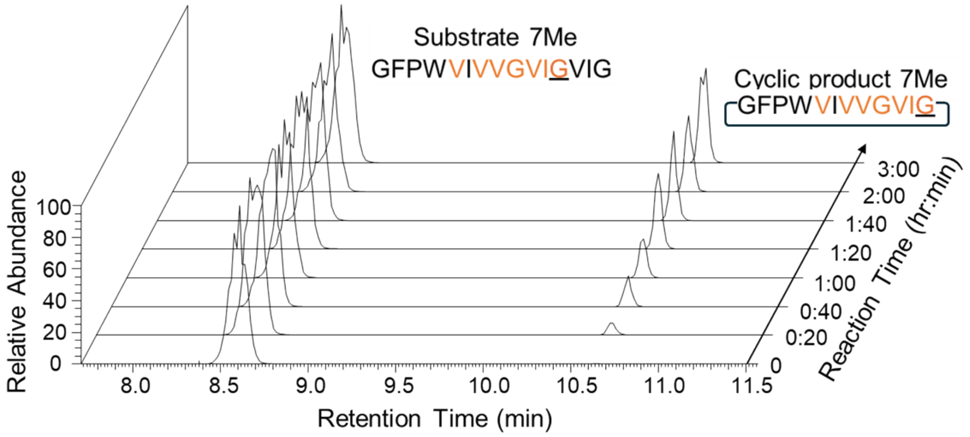

Substrate 7Me
GFPWVIVVGVIGVIG
Cyclic product 7Me
GFPWVIVVGVIG
Relative Abundance
100
80
60
40
20
0
8.0
8.5
9.0
9.5
10.0
10.5
11.0
11.5
Retention Time (min)
3:00
2:00
1:40
1:20
1:00
0:40
0:20
0
Reaction Time (hr:min)

f

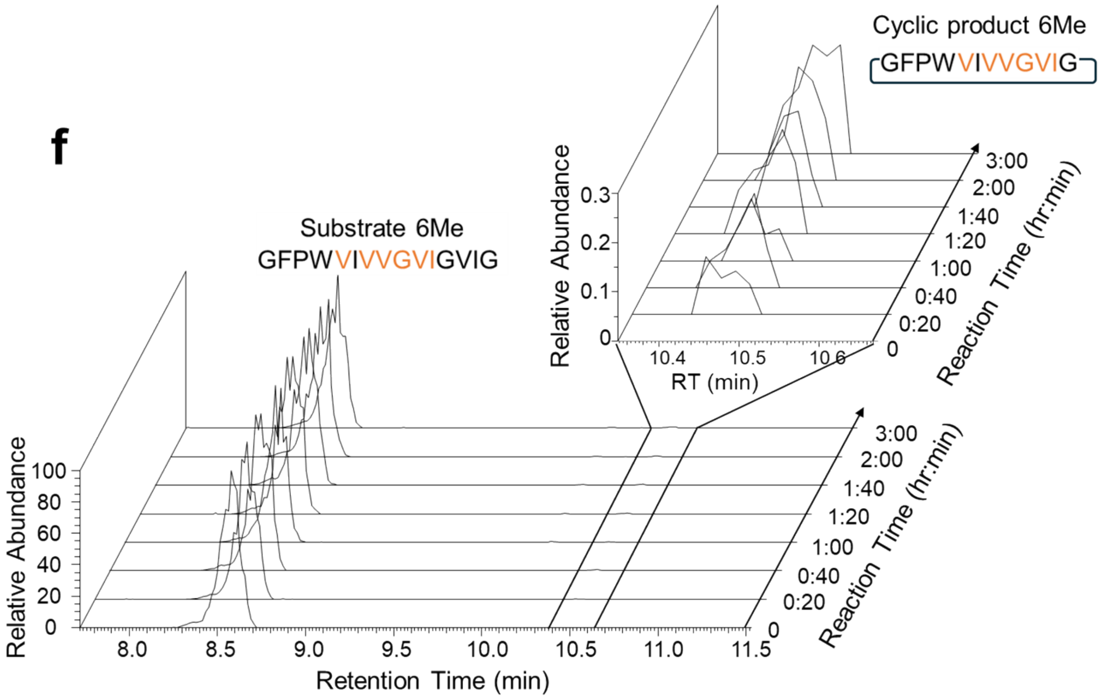

Cyclic product 6Me
GFPWVIVVGVIG
Relative Abundance
0.3
0.2
0.1
0
10.4
10.5
10.6
RT (min)
3:00
2:00
1:40
1:20
1:00
0:40
0:20
0
Reaction Time (hr:min)
Substrate 6Me
GFPWVIVVGVIGVIG
Relative Abundance
100
80
60
40
20
0
8.0
8.5
9.0
9.5
10.0
10.5
11.0
11.5
Retention Time (min)
3:00
2:00
1:40
1:20
1:00
0:40
0:20
0
Reaction Time (hr:min)

Figure S13. Activities of GmPOPB and OphP towards synthetic peptides PHA1 and AMA1. Peptides PHA1 and AMA1 are known to be processed by GmPOPB protease[1,5]. The graphs show the HPLC-UV-VIS chromatograms with optical density (OD) at 280nm. The synthetic peptides were stored at 1 mg/ml in phosphate buffer at pH 6.0. For the reaction, 20 µM substrate was used with 1 µM of OphP or GmPOPB in the standard assay buffer [50 mM HEPES pH 6.0 + 10 mM DTT]. The reaction mixture (50 µl) was incubated at 30°C, 600 rpm, overnight. The reaction was quenched by the addition of 50 µl methanol. After centrifugation at the highest speed at 4°C, 5 µl supernatant was used for HPLC-MS/MS analysis. No cyclic peptides were observed for PHA1 in the UV trace due to low abundance. The peak around 12 min, marked with an asterisk, is unspecific as it does not correspond to any product. The amanitin and phallacidin core peptides are underlined. The products are indicated by a blue arrow.

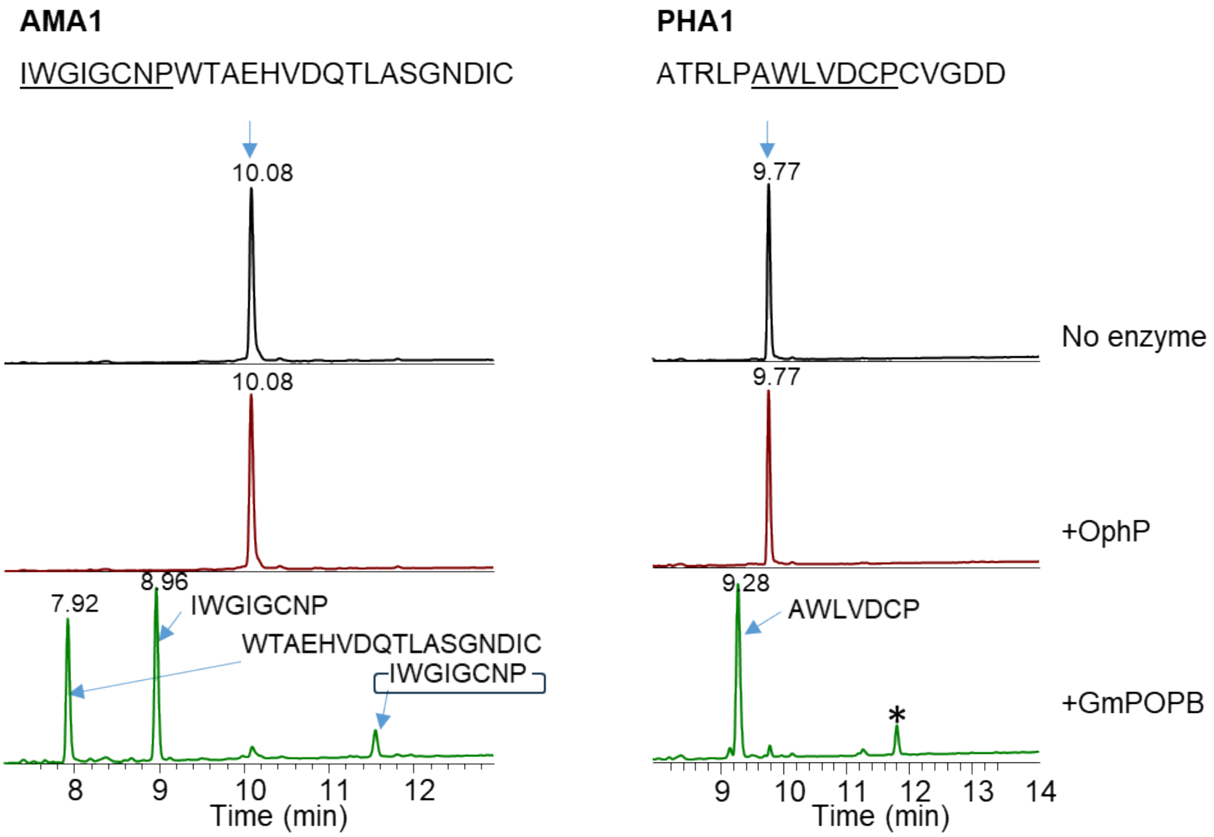

Figure S14. Overview, relative abundance and LC-MS/MS spectra of macrocyclic peptide products. Macrocyclic products obtained from incubation of OphP or GmPOPB with synthetic SFTI-1 variant peptides. (**a,b**) Overview and relative abundance of the obtained macrocyclic peptides. (**c-k**) LC-MS/MS with b-ions and y-ions in green are shown for one of the possible permutations for ring opening of the macrocyclic peptide. Mass tolerance between observed masses and the theoretical masses was set at 10 ppm. Mass precision was set at 4 decimals, but m/z values are rounded to two decimals in the figures. (**c**) cyclic TCTFSIPPICFPN^{me}G (**d**) cyclic TCTFSIPPICFPNP (**e**) cyclic TCTFSIPPICFPN^{me}A (**f**) cyclic TCTFSIPP (**g**) cyclic NGTCTFSIPPICFP (**h**) cyclic NGTCTFSIPP (**i**) cyclic TCTF^{me}SIPPICFPN^{me}G (**j**) cyclic TCTF^{me}SIPPICFP (**k**) cyclic TCTFSIPPICFP (**l**) cyclic ATRLPAWLVDCP (**m**) cyclic IWGIGCNPP (**n**) EIC peak area of SFTI-1A substrate and cyclic product NGTCTFSIPPICFP after incubation with or without GmPOPB

a

Peptide Substrate Name	Peptide substrate Sequence	Macrocyclic OphP product	Major macrocyclic GmPOPB product	Minor macrocyclic GmPOPB product
SFTI-1B	TCTFSIPPICFPNGSVMSTE	None	TCTFSIPP	None
SFTI-1C	TCTFSIPPICFPNGSVMSTE	TCTFSIPPICFPNG	TCTFSIPP	TCTFSIPPICFPNG
SFTI-1D	TCTFSIPPICFPNGSVMSTE	TCTFSIPPICFPNG	TCTFSIPPICFPNG	TCTFSIPPICFP
SFTI-1A	NGTCTFSIPPICFPWTAEHVDQTLASGNDIC	None	NGTCTFSIPPICFP	NGTCTFSIPP
SFTI-1E	TCTFSIPPICFPNGASGNDI	None	TCTFSIPP	TCTFSIPPICFP
SFTI-1F	TCTFSIPPICFPNGASGNDI	TCTFSIPPICFPNG	TCTFSIPP	TCTFSIPPICFPNG
SFTI-1G	TCTFSIPPICFPNGSIGNDA	None	TCTFSIPP	None
SFTI-1H	TCTFSIPPICFPNGSIGNDA	TCTFSIPPICFPNG	TCTFSIPPICFPNG	TCTFSIPP
SFTI-1I	TCTFSIPPICFPNASIGNDA	None	TCTFSIPP	TCTFSIPPICFP
SFTI-1J	TCTFSIPPICFPNASIGNDA	TCTFSIPPICFPNA	TCTFSIPPICFPNA	TCTFSIPP
SFTI-1M	TCTFSIPPICFPNPSIGNDA	TCTFSIPPICFPNP	TCTFSIPPICFPNP	TCTFSIPP
SFTI-1K	TCTFSIPPICFPNVSIGNDA	None	TCTFSIPP	TCTFSIPPICFP
SFTI-1L	TCTFSIPPICFPNVSIGNDA	None	TCTFSIPP	TCTFSIPPICFP
Oph-5^{high}	GFPWVIVVGVIGVIG	GFPWVIVVGVIG	None	None
PHA1	ATRLPAWLVDCPCVGDD	None	ATRLPAWLVDCP	None
AMA1	IWGIGCNPWTAEHVDQTLASGNDIC	None	IWGIGCNPP	None

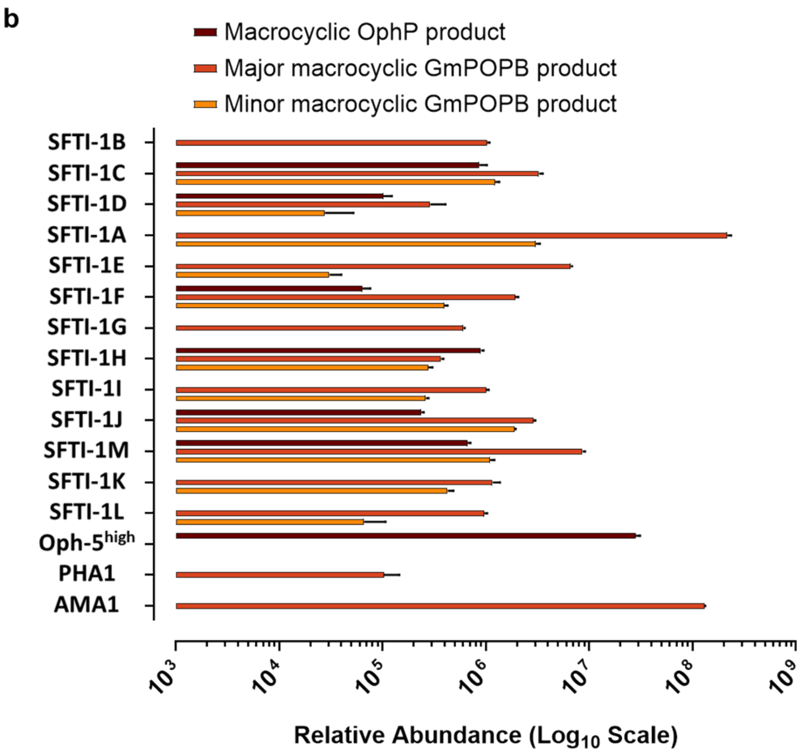
b
Macrocyclic OphP product
Major macrocyclic GmPOPB product
Minor macrocyclic GmPOPB product
SFTI-1B
SFTI-1C
SFTI-1D
SFTI-1A
SFTI-1E
SFTI-1F
SFTI-1G
SFTI-1H
SFTI-1I
SFTI-1J
SFTI-1M
SFTI-1K
SFTI-1L
Oph-5high
PHA1
AMA1
Relative Abundance (Log10 Scale)

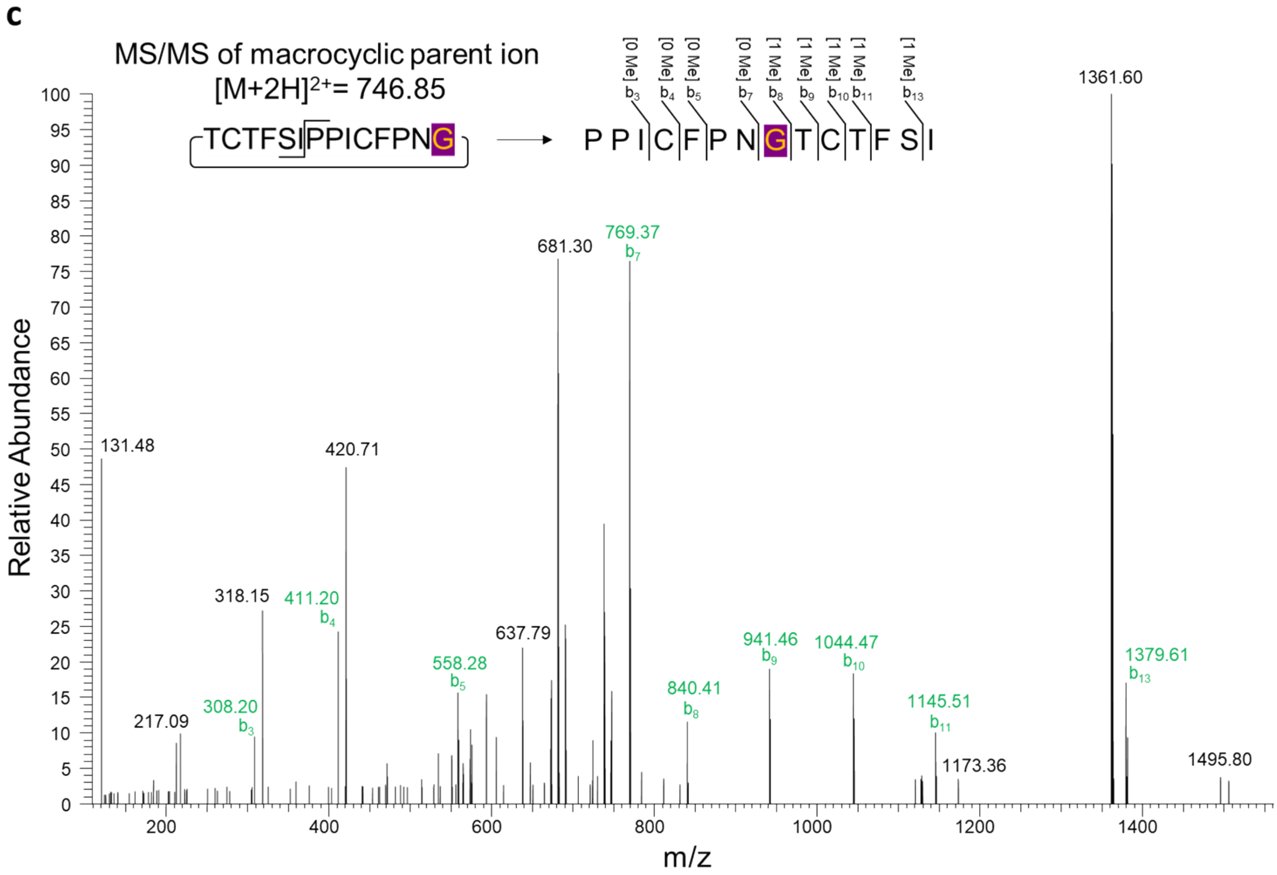
c
MS/MS of macrocyclic parent ion
[M+2H]2+= 746.85
TCTFSIPPICFPNG
P P I C F P N G T C T F S I
Relative Abundance
m/z
131.48
217.09
308.20 b3
318.15
411.20 b4
420.71
558.28 b5
637.79
681.30
769.37 b7
840.41 b8
941.46 b9
1044.47 b10
1145.51 b11
1173.36
1361.60
1379.61 b13
1495.80

d

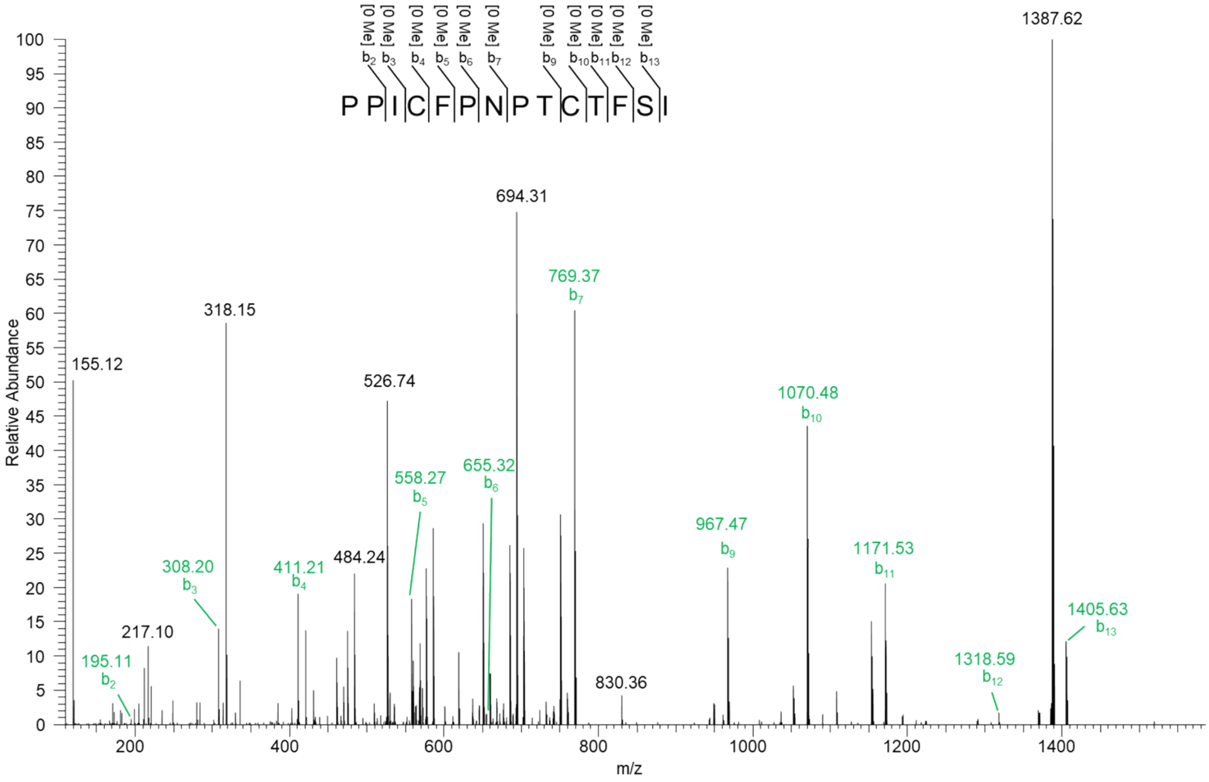

e

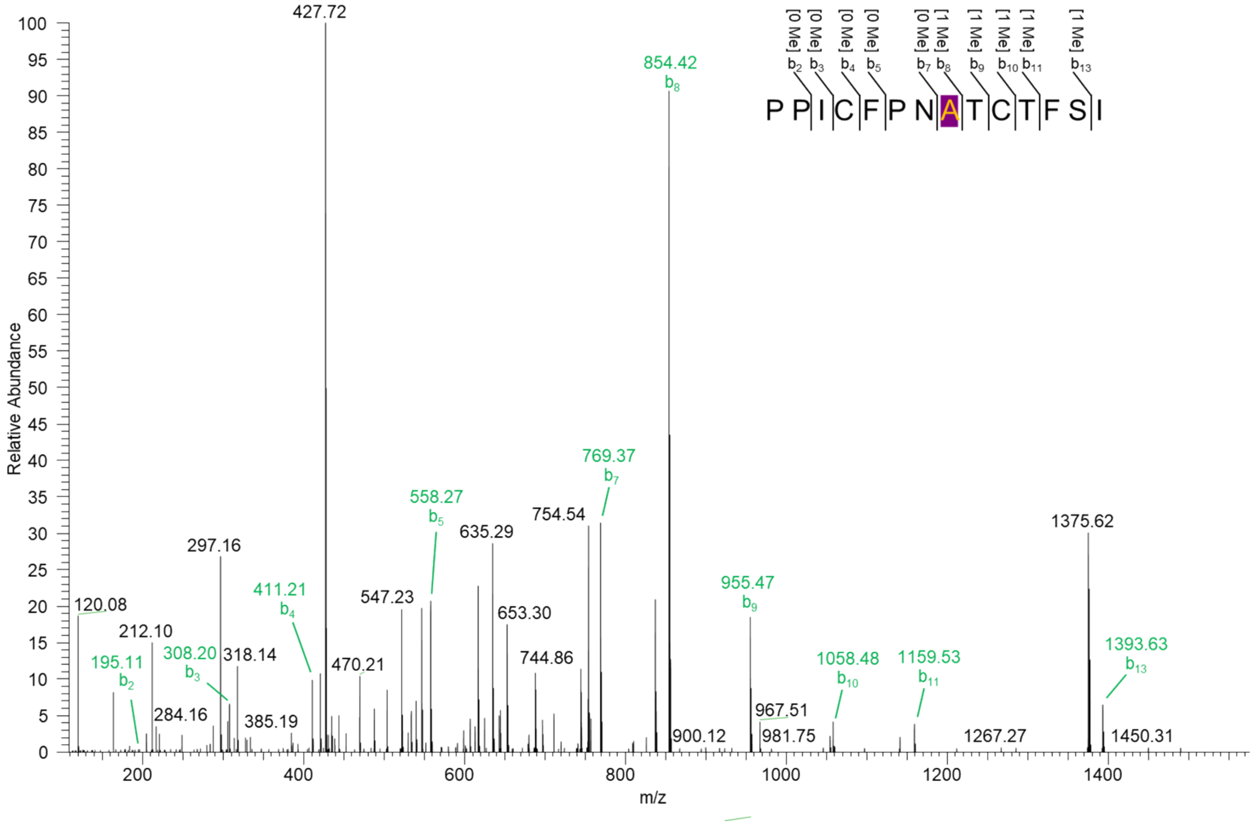

f

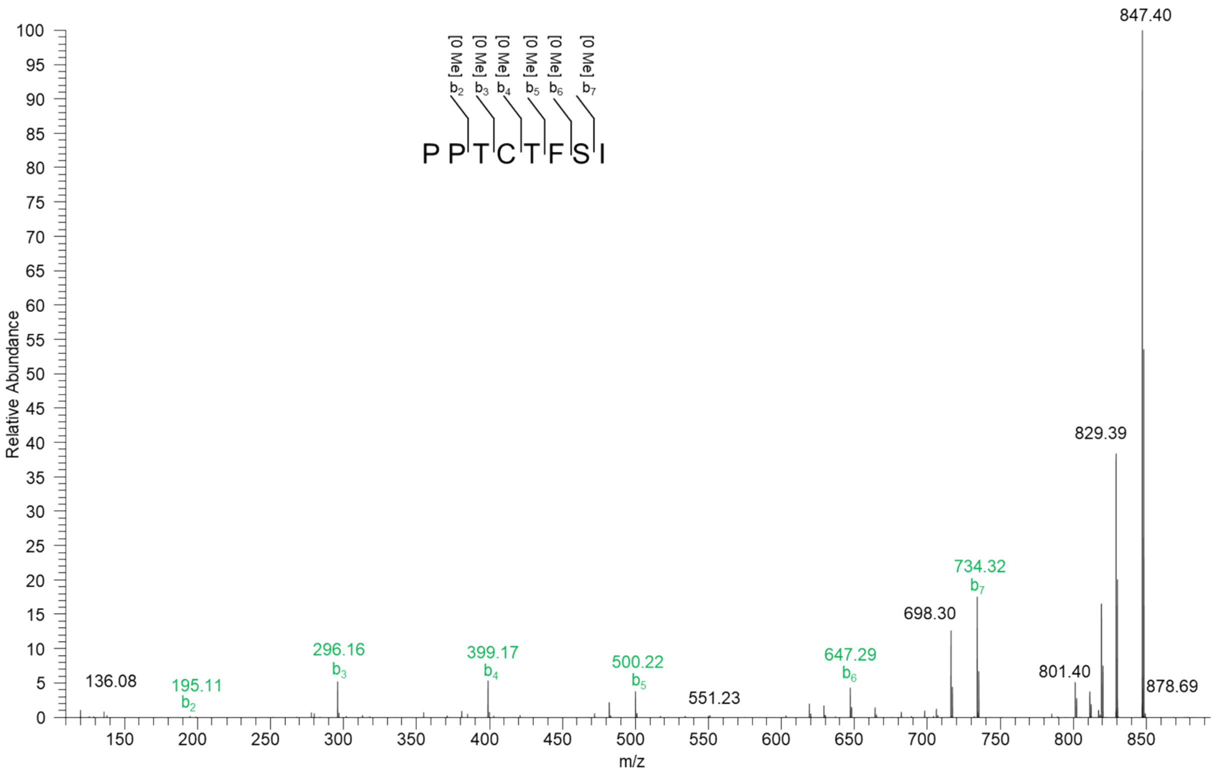

PPTCTFSI
[0 Me] b2
[0 Me] b3
[0 Me] b4
[0 Me] b5
[0 Me] b6
[0 Me] b7
847.40
829.39
734.32 b7
698.30
647.29 b6
500.22 b5
399.17 b4
296.16 b3
195.11 b2
136.08
551.23
801.40
878.69
Relative Abundance
m/z

g

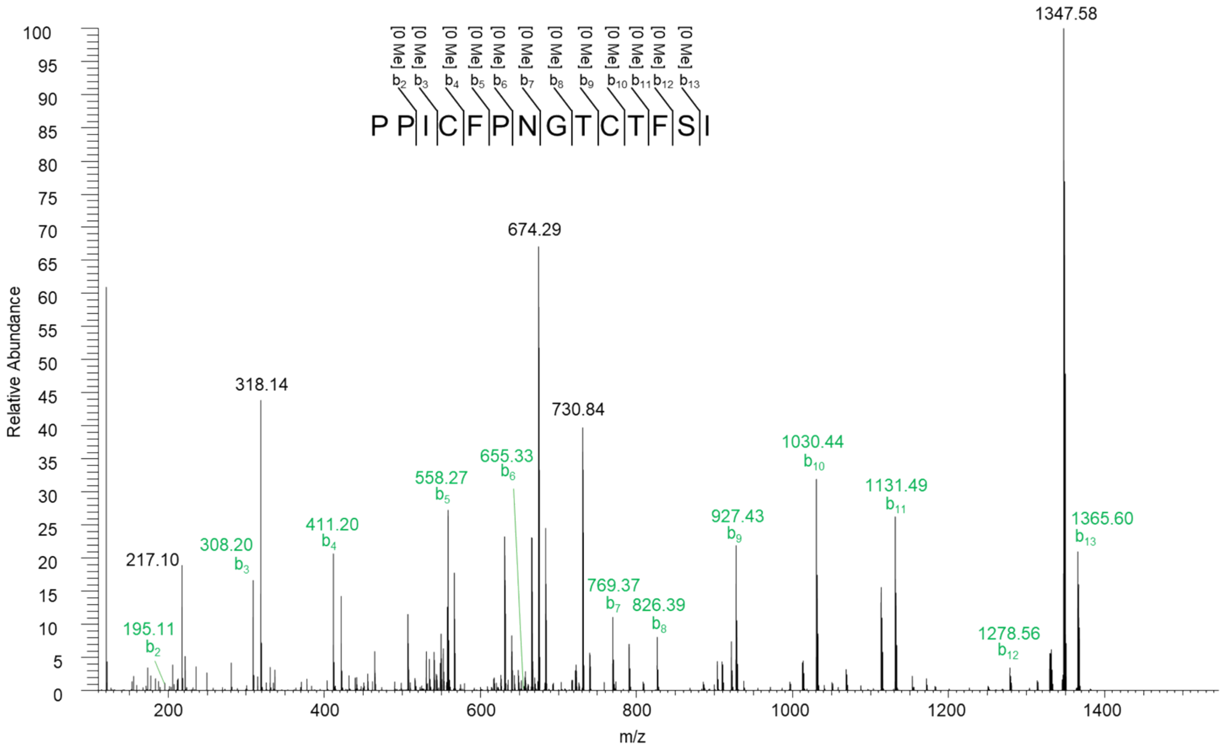

PPICFPNGTCTFSI
[0 Me] b2
[0 Me] b3
[0 Me] b4
[0 Me] b5
[0 Me] b6
[0 Me] b7
[0 Me] b8
[0 Me] b9
[0 Me] b10
[0 Me] b11
[0 Me] b12
[0 Me] b13
1347.58
674.29
318.14
730.84
1030.44 b10
1131.49 b11
655.33 b6
558.27 b5
927.43 b9
1365.60 b13
411.20 b4
308.20 b3
217.10
769.37 b7
826.39 b8
195.11 b2
1278.56 b12
Relative Abundance
m/z

h

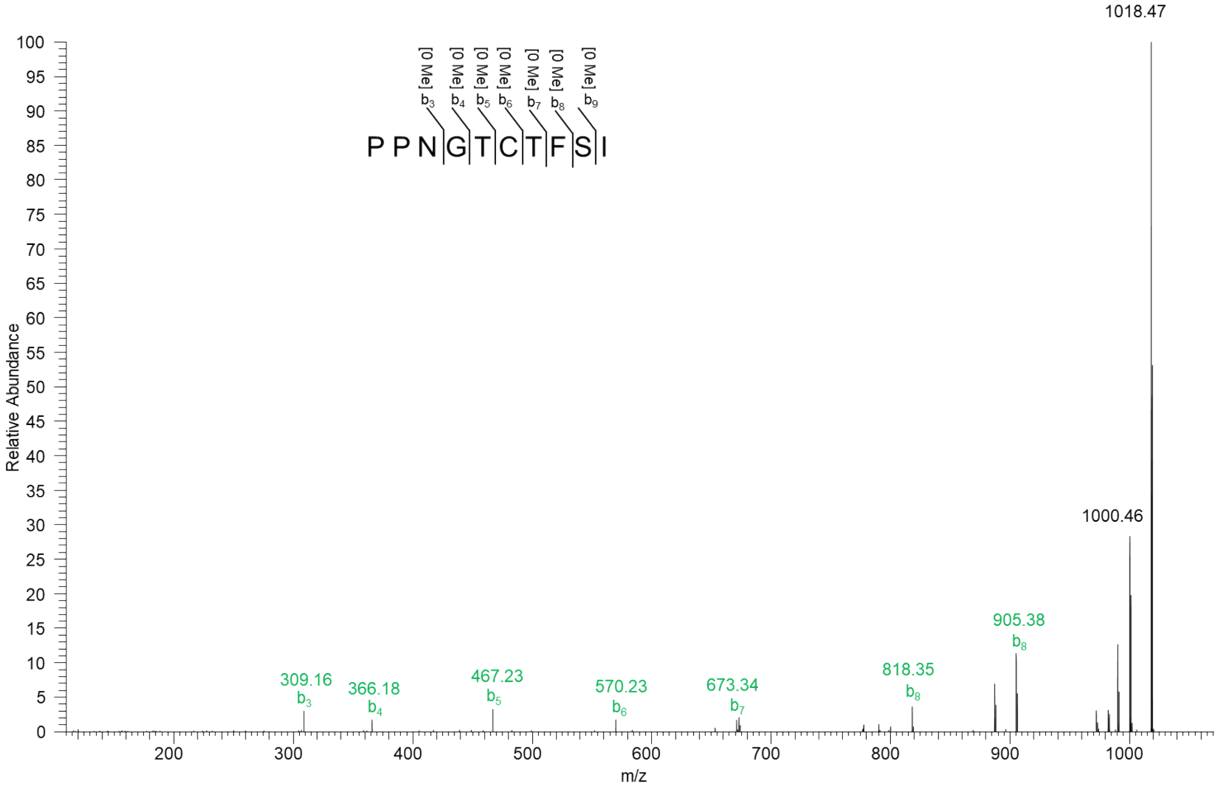

1018.47
1000.46
905.38
b8
818.35
b8
673.34
b7
570.23
b6
467.23
b5
366.18
b4
309.16
b3
[0 Me] b3
[0 Me] b4
[0 Me] b5
[0 Me] b6
[0 Me] b7
[0 Me] b8
[0 Me] b9
P P N G T C T F S I
Relative Abundance
m/z

i

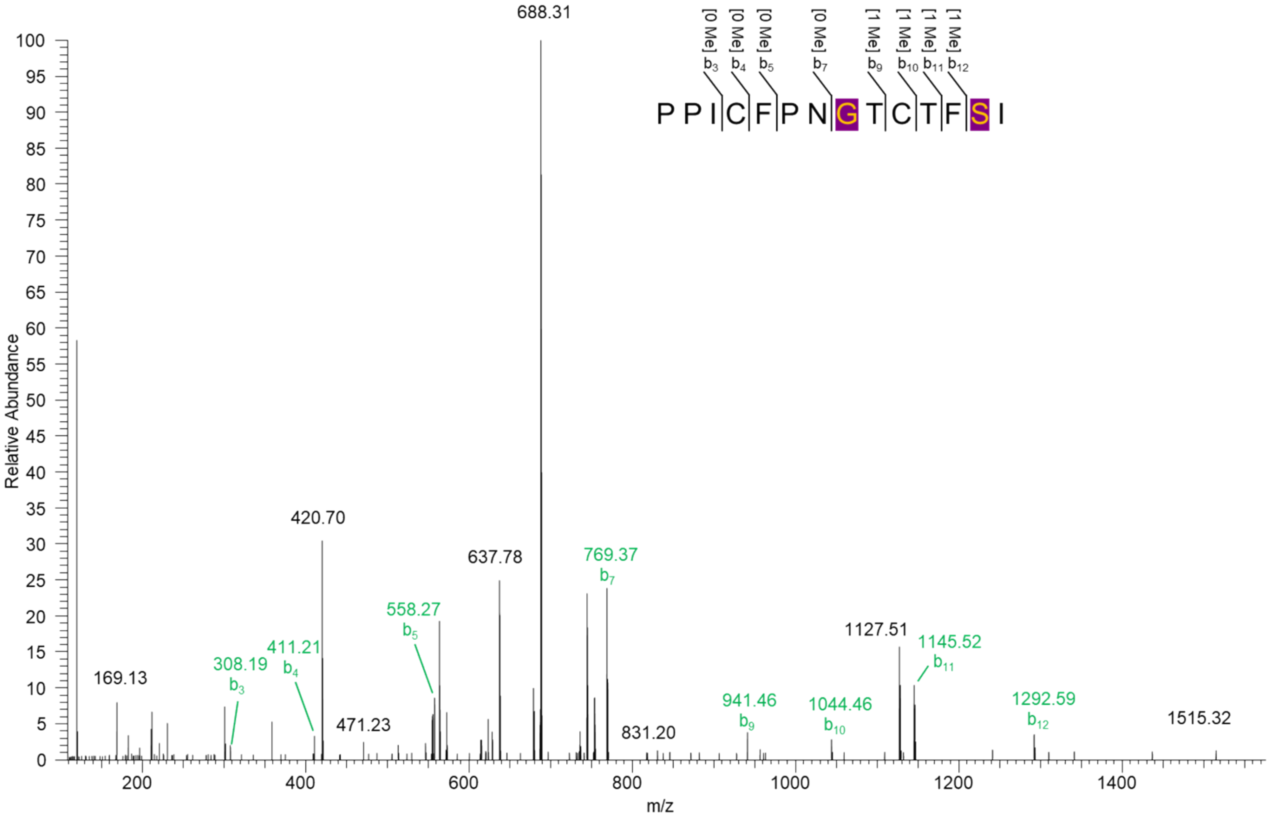

688.31
420.70
637.78
769.37
b7
558.27
b5
411.21
b4
308.19
b3
169.13
471.23
831.20
941.46
b9
1044.46
b10
1127.51
1145.52
b11
1292.59
b12
1515.32
[0 Me] b3
[0 Me] b4
[0 Me] b5
[0 Me] b7
[1 Me] b9
[1 Me] b10
[1 Me] b11
[1 Me] b12
P P I C F P N G T C T F S I
Relative Abundance
m/z

j

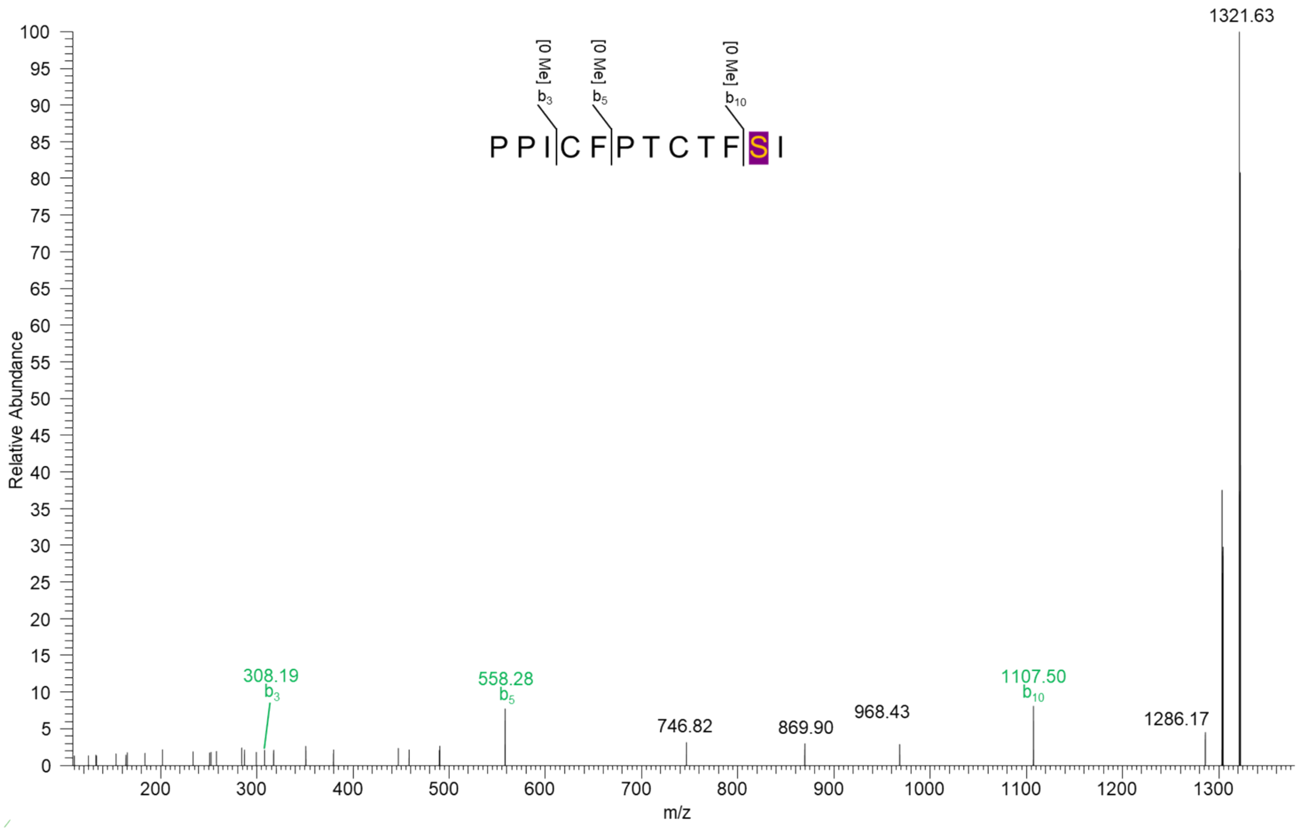
1321.63
Relative Abundance
m/z
PPICFPTCTFSI
[0 Me] b3
[0 Me] b5
[0 Me] b10
308.19 b3
558.28 b5
746.82
869.90
968.43
1107.50 b10
1286.17

k

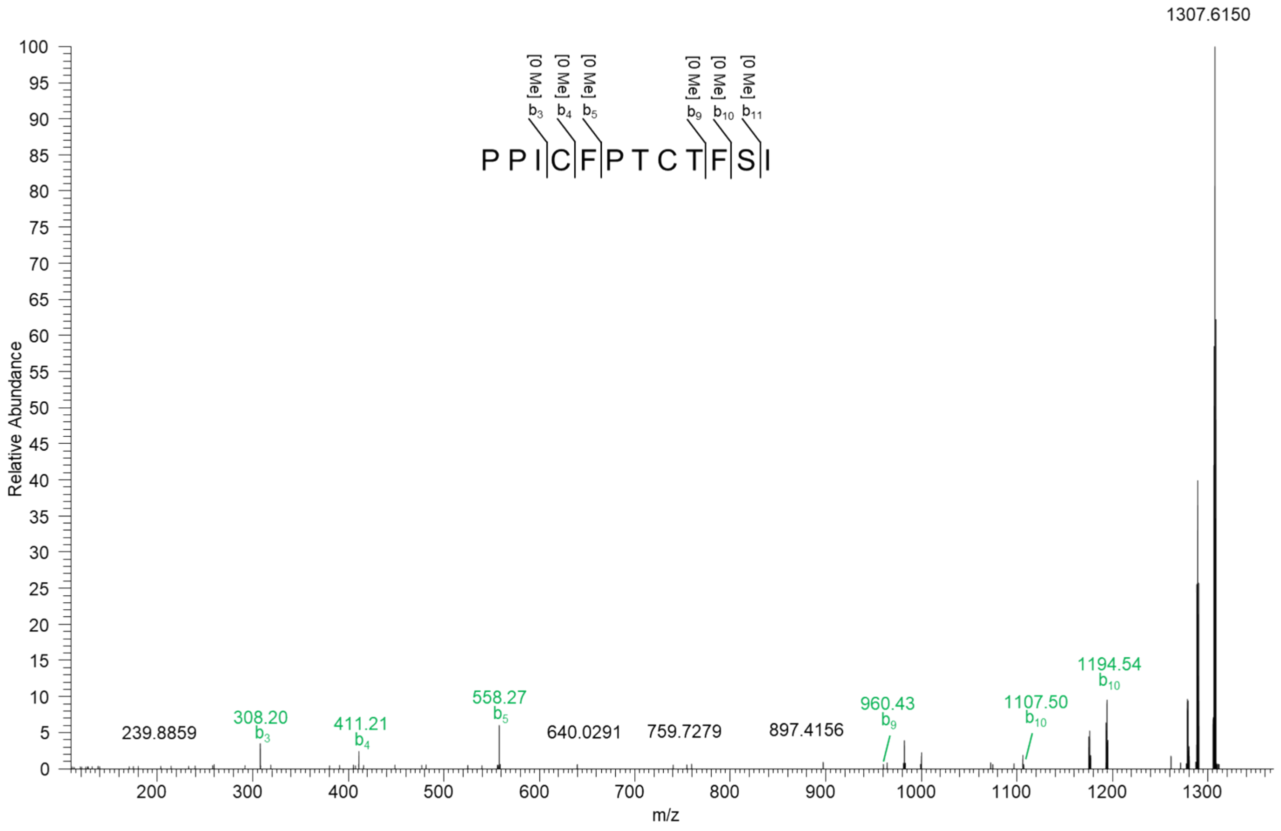
1307.6150
Relative Abundance
m/z
PPICFPTCTFSI
[0 Me] b3
[0 Me] b4
[0 Me] b5
[0 Me] b9
[0 Me] b10
[0 Me] b11
239.8859
308.20 b3
411.21 b4
558.27 b5
640.0291
759.7279
897.4156
960.43 b9
1107.50 b10
1194.54 b10

l

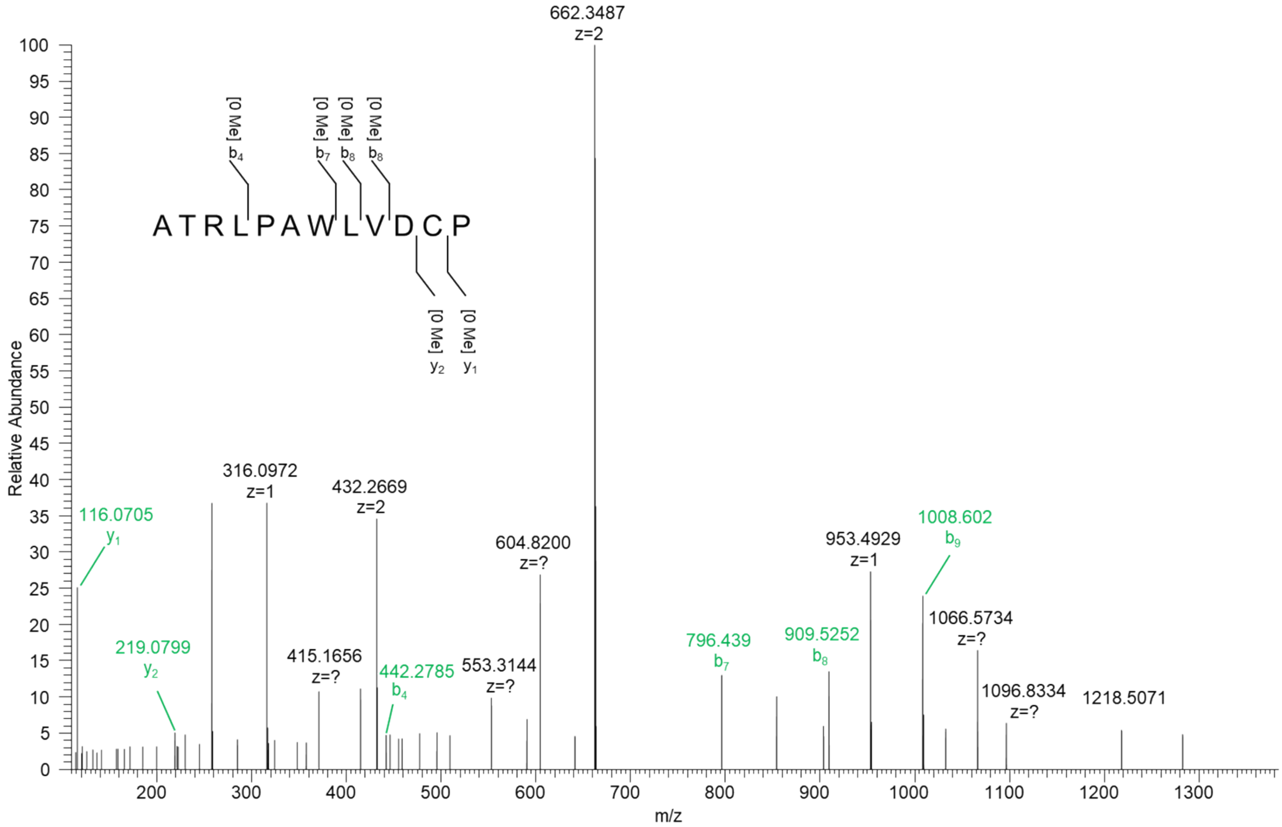

ATRLPAWLVDCP
[0 Me] b4
[0 Me] b7
[0 Me] b8
[0 Me] b8
[0 Me] y2
[0 Me] y1
662.3487 z=2
316.0972 z=1
432.2669 z=2
604.8200 z=?
953.4929 z=1
1008.602 b9
116.0705 y1
219.0799 y2
415.1656 z=?
442.2785 b4
553.3144 z=?
796.439 b7
909.5252 b8
1066.5734 z=?
1096.8334 z=?
1218.5071
Relative Abundance
m/z

m

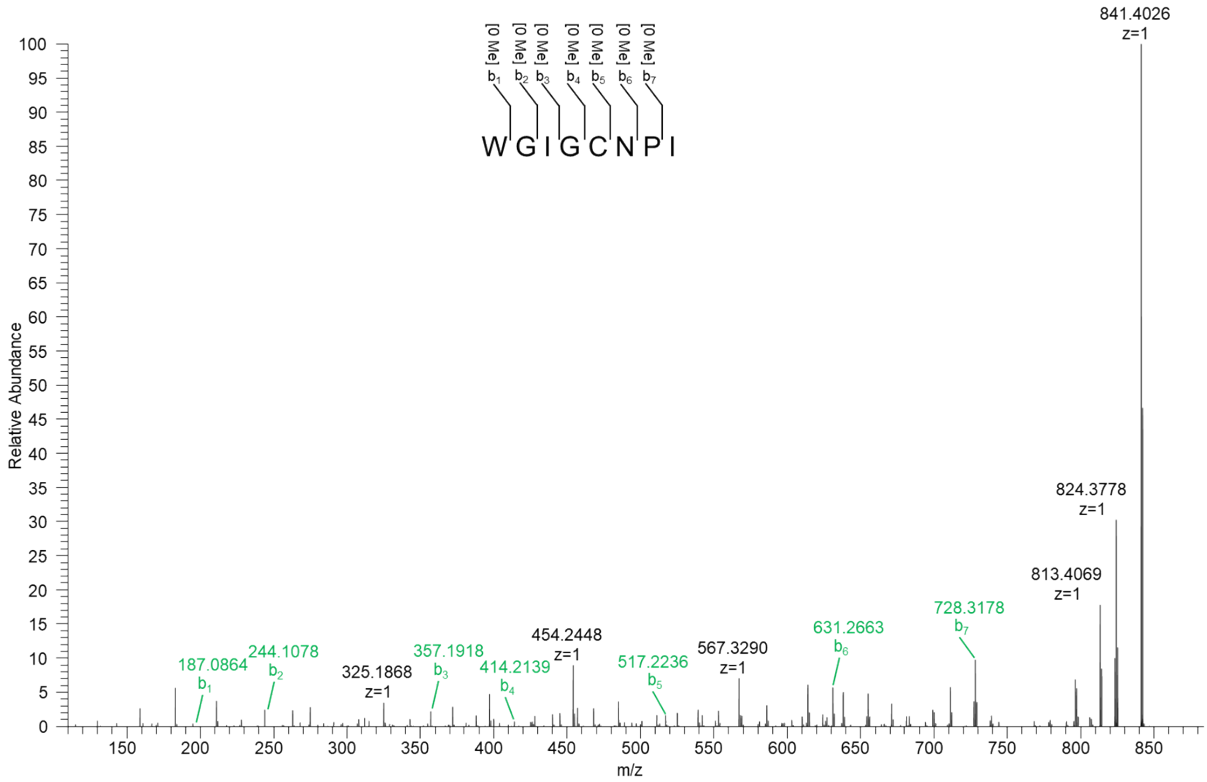

WGIGCNPI
[0 Me] b1
[0 Me] b2
[0 Me] b3
[0 Me] b4
[0 Me] b5
[0 Me] b6
[0 Me] b7
841.4026 z=1
824.3778 z=1
813.4069 z=1
728.3178 b7
631.2663 b6
567.3290 z=1
517.2236 b5
454.2448 z=1
414.2139 b4
357.1918 b3
325.1868 z=1
244.1078 b2
187.0864 b1
Relative Abundance
m/z

n

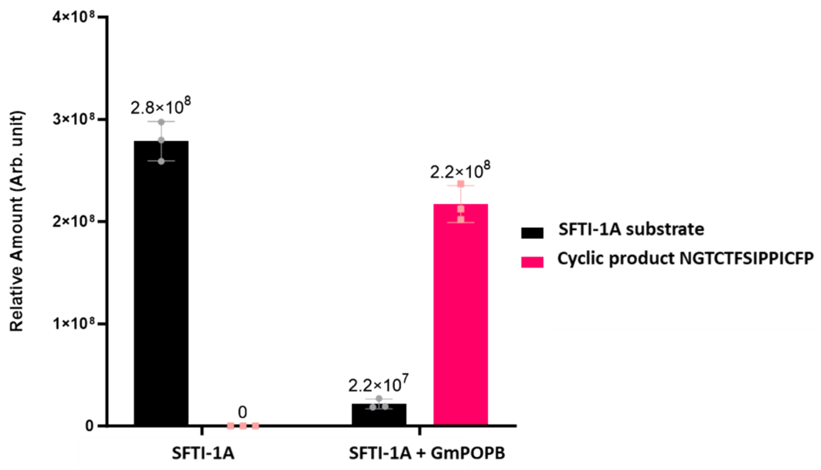

4×10⁸
3×10⁸
2×10⁸
1×10⁸
0
Relative Amount (Arb. unit)
2.8×10^8
0
2.2×10^7
2.2×10^8
SFTI-1A
SFTI-1A + GmPOPB
SFTI-1A substrate
Cyclic product NGTCTFSIPPICFP

Figure S15. Cartoon and electrostatic surfaces of apo OphP (PDB 7ZB2), apo GmPOPB (PDB 5N4F) and apo human dipeptidyl peptidase IV (DPP-IV, PDB 1NU6). The view through the β-propeller and side views of (**a**) apo OphP with the Oph-6 substrate (yellow spheres), (**b**) apo GmPOPB with the 35mer substrate (yellow spheres), and (**c**) apo DPP-IV with modelled tripeptide substrate (yellow spheres). Electrostatic calculation was performed using the APBS-PDB2PQR software suite under the default setting and drawn in PyMol. The electrostatic [illegible]

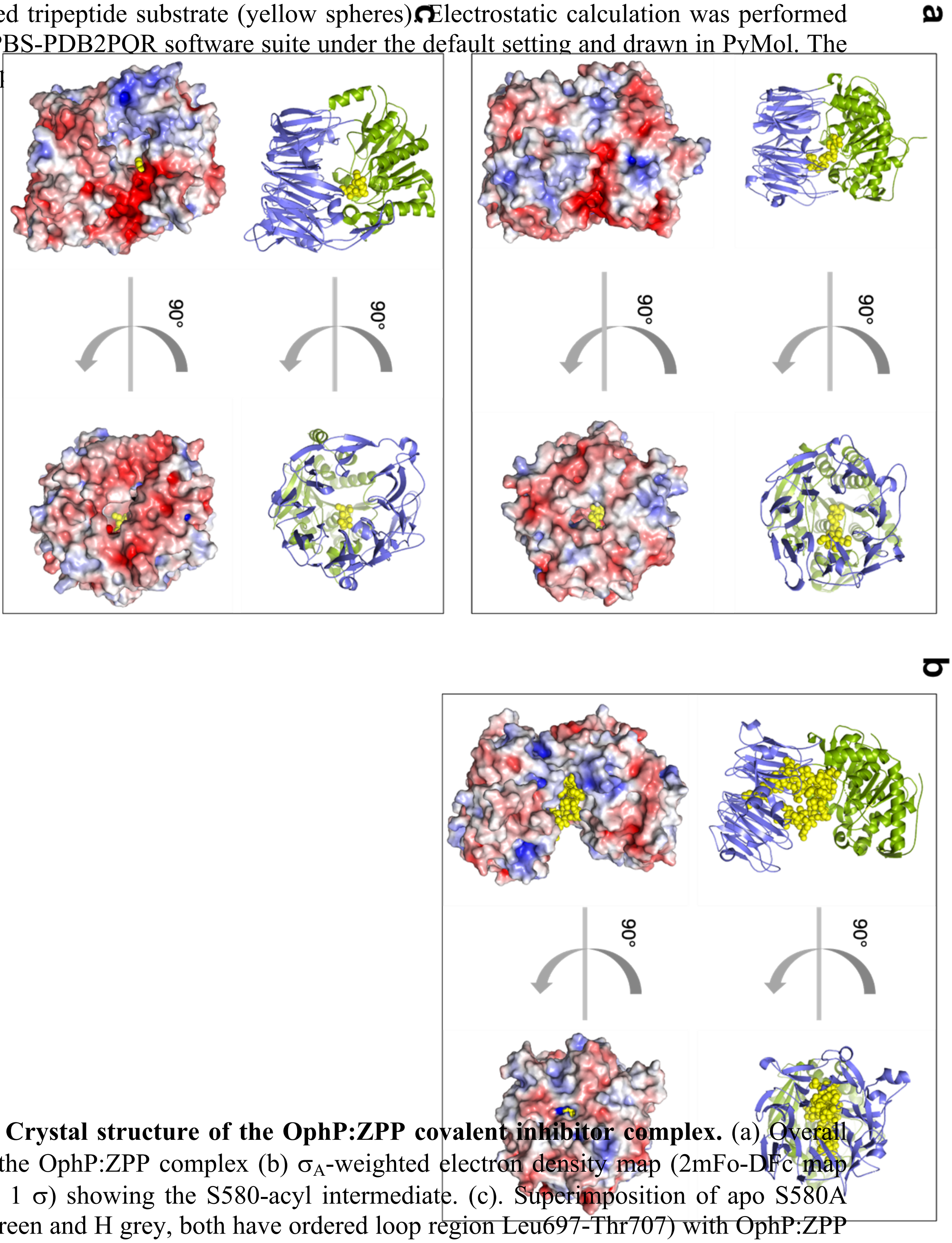

Figure S16. Crystal structure of the OphP:ZPP covalent inhibitor complex. (a) Overall structure of the OphP:ZPP complex (b) σ_A-weighted electron density map (2mFo-DFc map contoured at 1 σ) showing the S580-acyl intermediate. (c). Superimposition of apo S580A (subunit A green and H grey, both have ordered loop region Leu697-Thr707) with OphP:ZPP complex (subunit A colored in salmon). The black arrows illustrate the large movement of two loops (Ser164-Met171, and Leu697-Thr707) when ZPP is bound. As a result of the 7.6 Å shift of His701 it is now part of the catalytic triad in the OphP:ZPP complex. The movement of Asp156 upon ZPP results in a salt bridge and Arg667 in the OphP:ZPP complex.

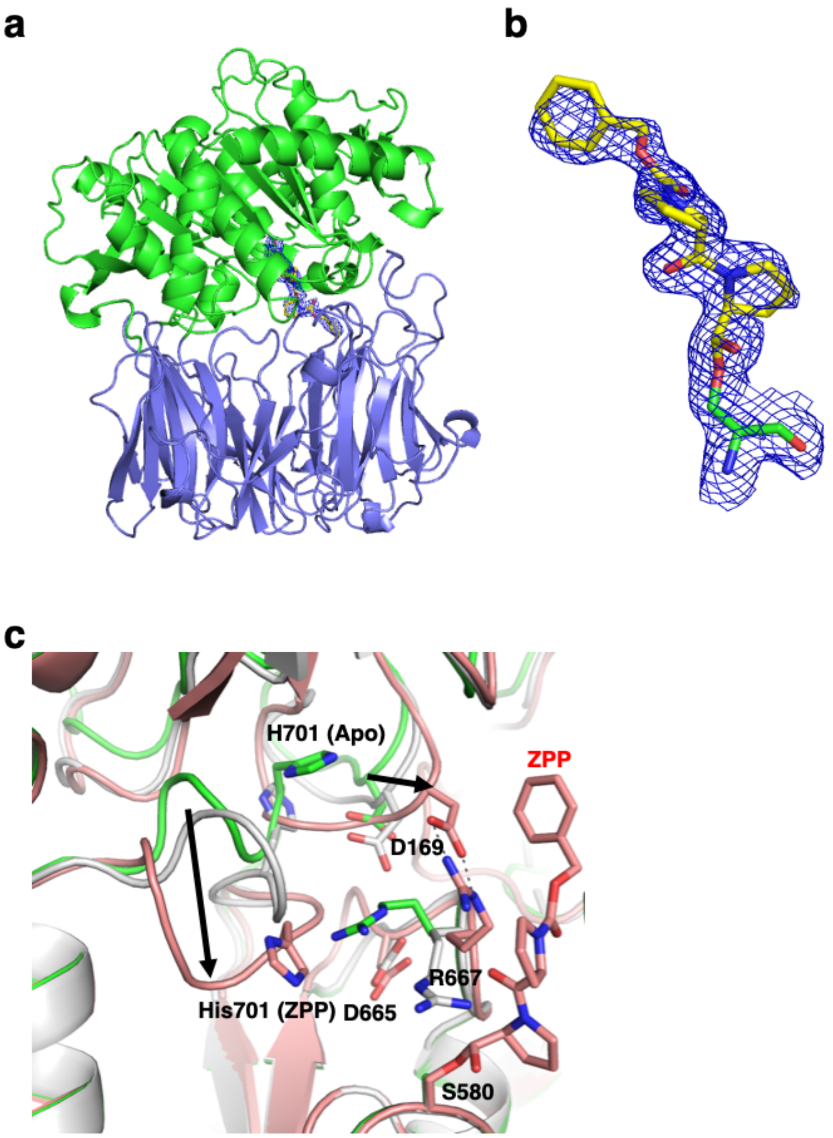
a
b
c
H701 (Apo)
ZPP
D169
R667
His701 (ZPP)
D665
S580

Figure S17. Multiple sequence alignment of OphP with post-proline cleaving prolyl oligopeptidases (POPs). The sequence of OphP is used as a query and blasted (protein-protein BLAST) against the UniProtKB/SwissProt database. Sequences are further selected by removing all the dipeptidyl peptidase family enzyme sequences. Multiple sequence alignment was created with MUSCLE [15] and rendered as white and black with ESPript 3.0[16]. The hydrophobic pocket residues accommodating P1 proline are highlighted by underlying solid red triangles and catalytic triad residues are shown in solid purple triangles. Secondary structure elements of OphP are depicted above the aligned sequence. Black box with white character shows strict identity, bold black character similarity, and black frame similarity across groups. Names, UniProt ID and corresponding organisms are as follows: PCY1, R4P353, *Gypsophila vaccaria*; GmPOPB, H2E7Q8, *Galerina marginata*; GmPOPA, H2E7Q7, *Galerina marginate*; AbPOPB, E2JFG2, *Amanita bisporigera*; AbPOPA, E2JFG1, *Amanita bisporigera*; PfuPOP, Q51714 (PDB entry 5T88), *Pyrococcus furiosus*; AhPOP, Q06903, *Aeromonas hydrophila*; EmPOP1, P27028, *Elizabethkingia meningoseptica*; EmPOP2, P27195, *Elizabethkingia meningoseptica*; DbPOP, Q86AS5, *Dictyostelium discoideum*; MmPOP, Q9QUR6, *Mus musculus*; P48147, *Homo sapiens*; BtPOP, Q9XTA2, *Bos taurus*; SsPOP, P23687 (PDB entry 1QFS), *Sus scrofa*. Sequence of DbiP from *Dendrothele bispora* is retrieved from Joint Genome Institute website (name: gm1.24165_g, protein ID: 871165, location: scaffold_621:21283-24607). The sequence of LedP from *Lentinula* edodes is obtained from NCBI database (GAW09065).

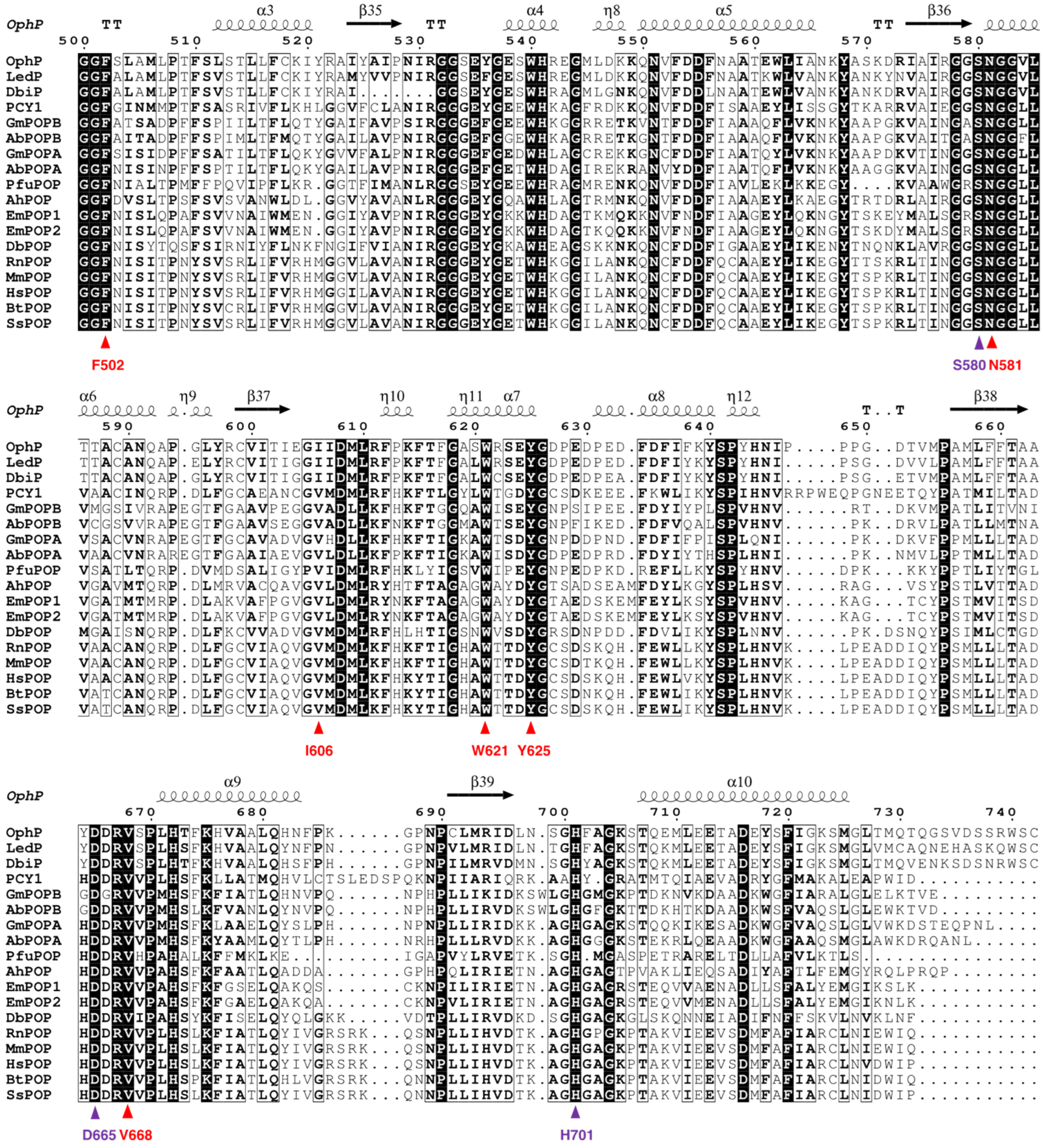
F502
S580 N581
I606
W621 Y625
D665 V668
H701

Figure S18. Surface of the P1 pocket of GmPOPB (PDB 5N4C), PCY1 (PDB 5UW6) and OphP (PDB 7ZAZ and 7ZB1) (all white) accommodating the covalent serine acyl intermediate of ZPP (yellow). (**a,b**) The canonical P1 pockets of GmPOPB and PCY1 can accommodate proline well at the cleavage P1 site. (**c,d**) Accommodation of the proline residue of ZPP in OphP (PDB 7ZAZ) requires conformational change of Ile606 compared to OphP-Oph-6 (PDB 7ZB1). In latter conformation, Cδ of I606 is only 2.3 Å away from Cβ of proline, producing a sterical clash and explaining the low efficiency of OphP towards substrates with a proline at the P1 site.

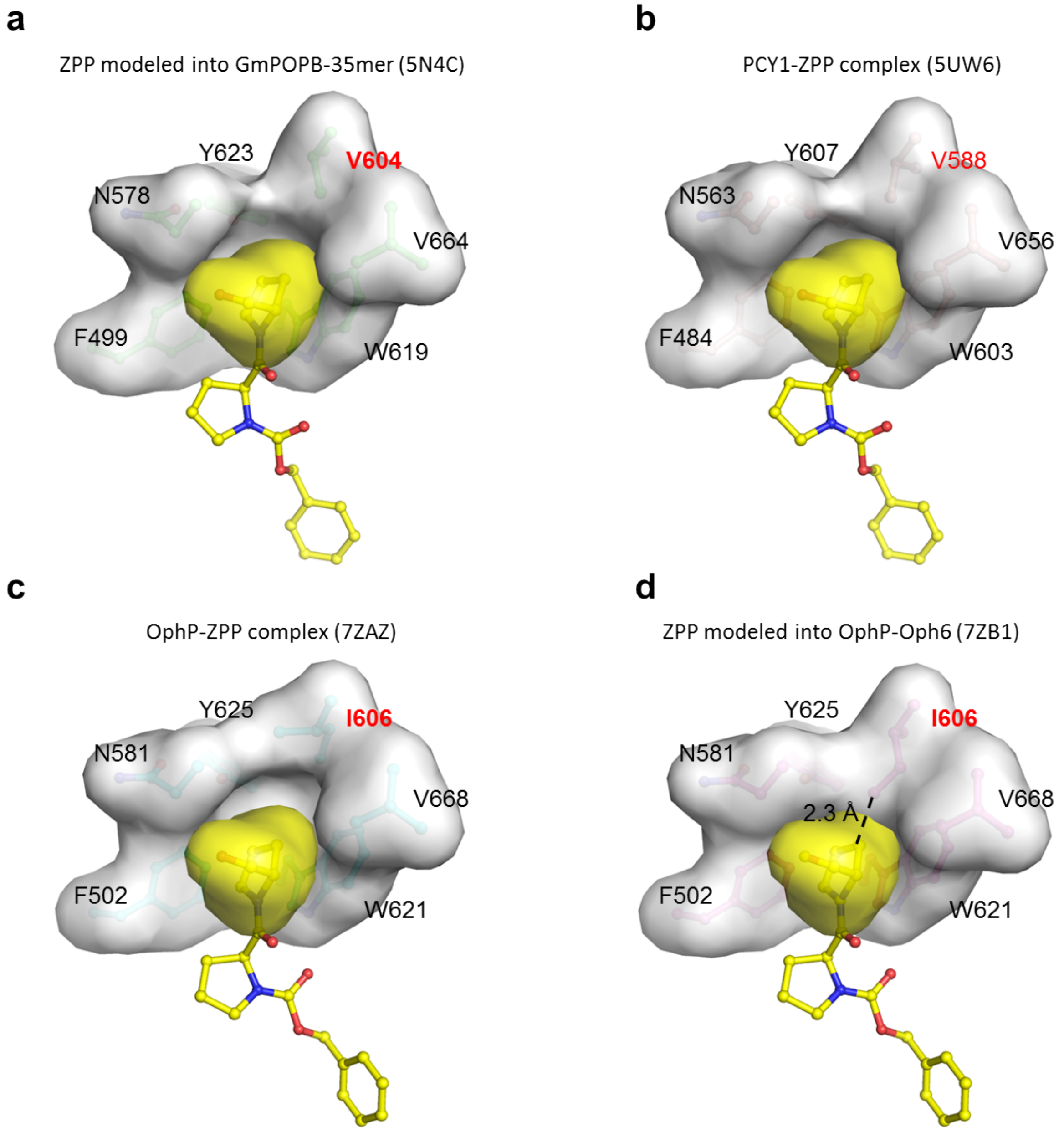

Figure S19. Crystal structures of OphP in complex with Oph-5. σ_A-weighted 2mFo-DFc map (contoured at 1) for Oph-5 (**a**) complexed with OphP S580A. Active site view of OphP: Oph-5 (**b**) in the complex structure showing the interactions. Side chains of the active site residues are shown in sticks with carbon green, nitrogen blue, oxygen red and the methyl groups orange. Broken lines show hydrogen bonds.

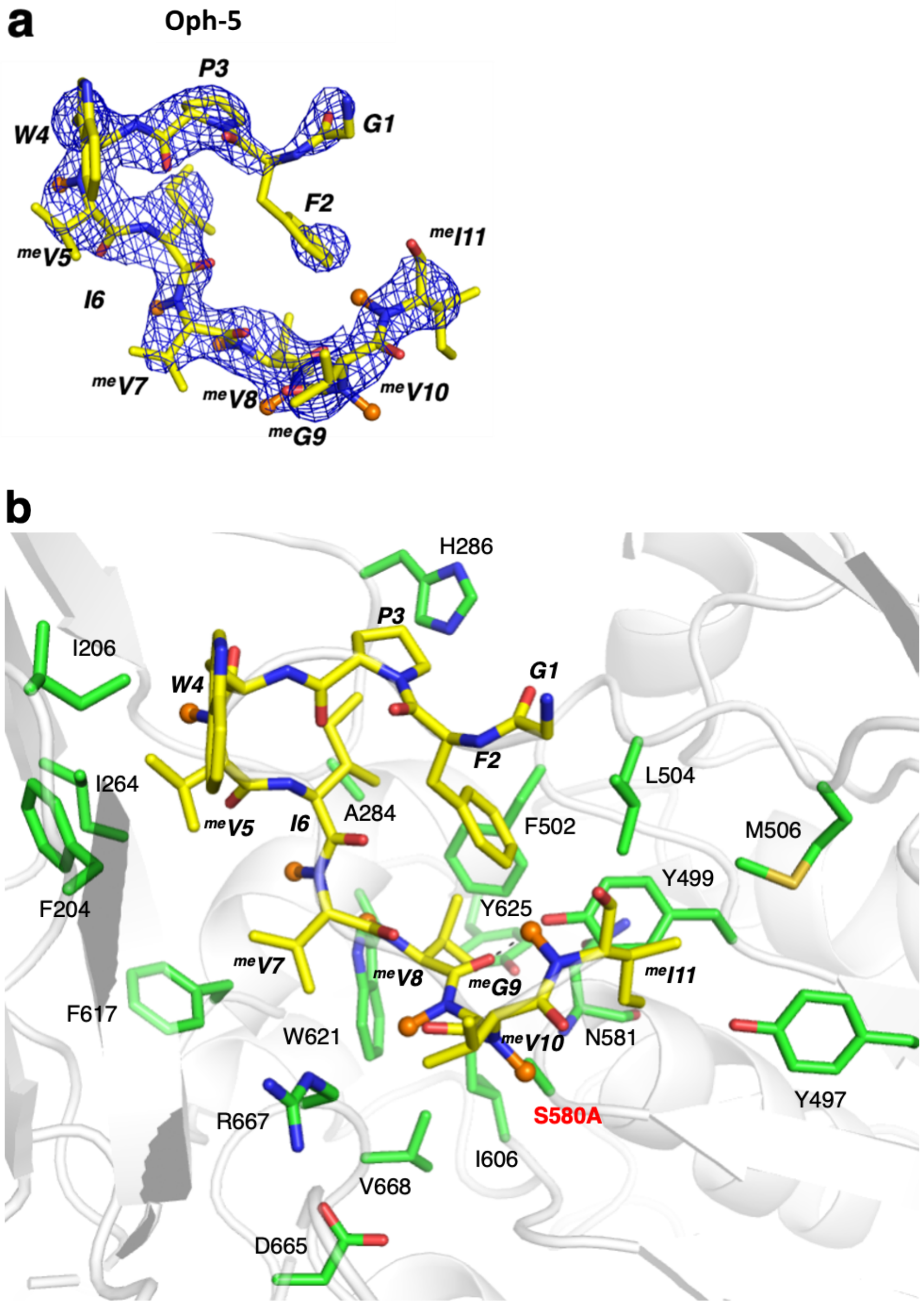

Figure S20. ITC experiments between OphP(S580A) and residues from the clasp domain (MPSSLLDAARESGEEASQNGFP), follower peptide (SVMSTE, and VIGSVMSTE), and Oph-5. Residues from the clasp domain (**a**) and follower peptides (**b** and **c**) showed no binding up to 2 mM in the presence of 50 μM of OphP(S580A). Oph-5 binds to OphP(S580A) with a K_D of 1.5 μM (**d**). The positive value of ΔH showed that the binding is endothermic suggesting the exclusion of water solvent during binding.

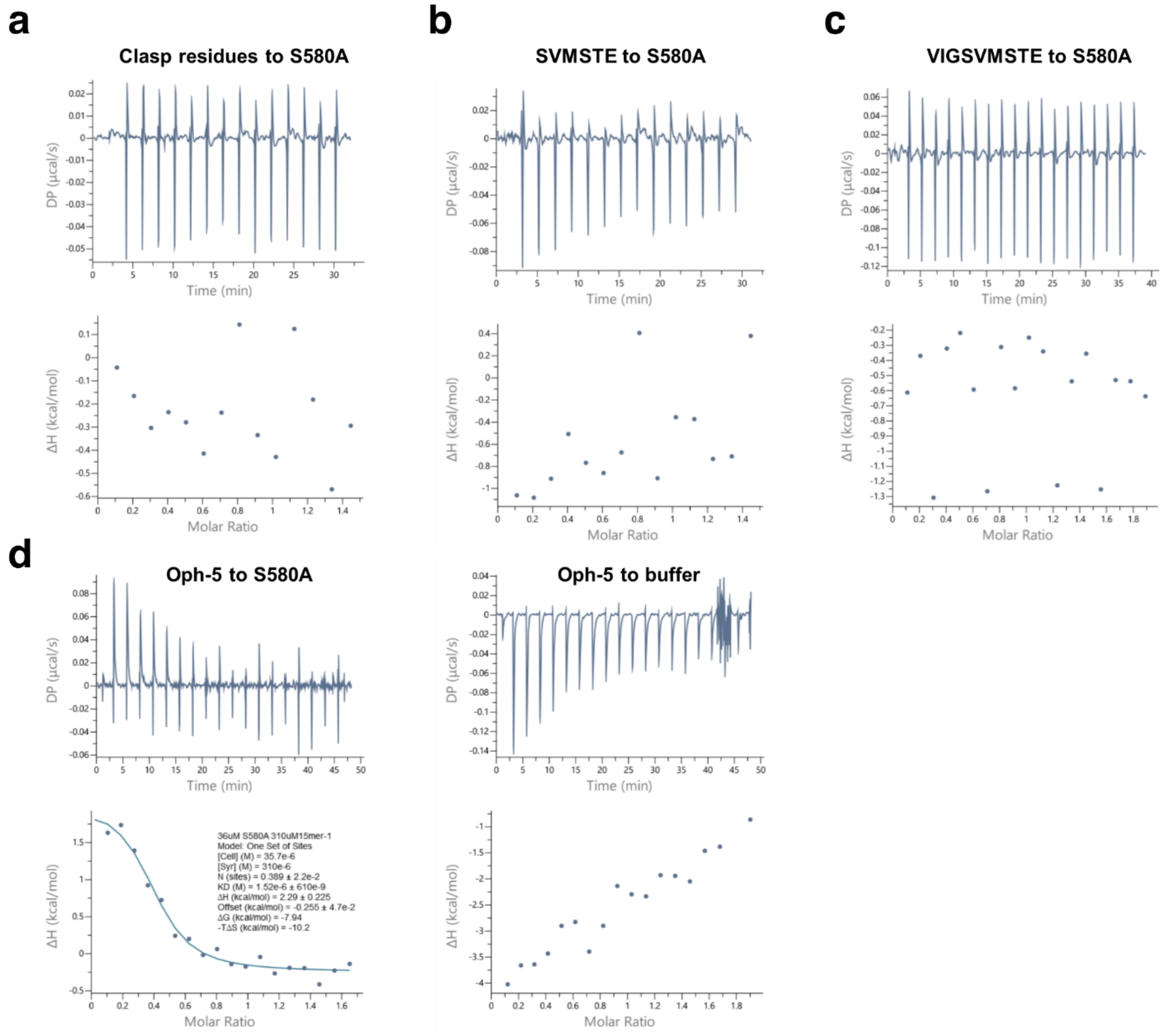

Figure S21. Production of omphalotin A in *P. pastoris* and *E. coli*. Production of omphalotin A in *P. pastoris* was performed as described previously[2,4]. In *E. coli,* production of omphalotin A was attempted by co expression of *ophMA* and *ophP* under the control of a T7 promoter. *E. coli* was grown in TB medium until OD_{600} 1.5 and expression was induced by addition of 0.2 mM IPTG. The induced cultures were incubated for five days at 16 °C. The bacterial cells were lysed using 0.1 mm glass beads in a FastPrep device and omphalotin A was extracted using a 1:1 mixture of ethyl acetate and n-hexane. The organic phase was evaporated, the pellets were redissolved in 100 µl of methanol and 10 µl of the solution was subjected to HPLC-MS/MS analysis. The graph shows the extracted ion chromatogram (EIC) peak area of omphalotin A extracted from *P. pastoris* and *E. coli* adjusted to culture volume.

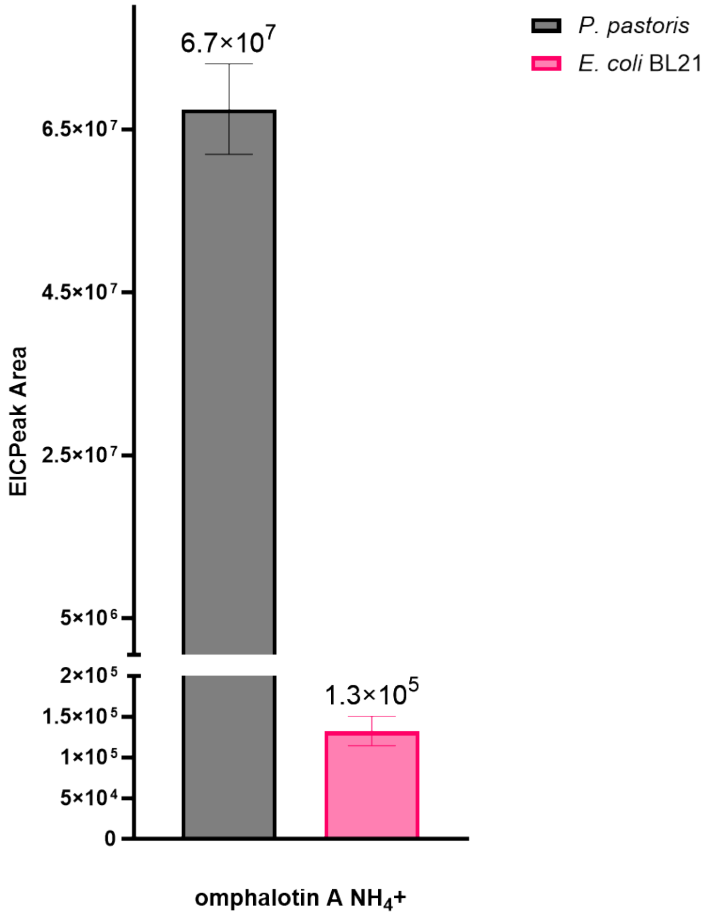

References

[1] C. M. Czekster, H. Ludewig, S. A. McMahon, J. H. Naismith, *Nat. Commun.* **2017**, *8*, 1045.

[2] E. Matabaro, H. Song, C. Chepkirui, H. Kaspar, L. Witte, J. H. Naismith, M. F. Freeman, M. Künzler, *Methods Enzymol.* **2021**, *656*, 429–458.

[3] H. Song, N. S. Van Der Velden, S. L. Shiran, P. Bleiziffer, C. Zach, R. Sieber, A. S. Imani, F. Krausbeck, M. Aebi, M. F. Freeman, M. Künzler, J. H. Naismith, *Sci. Adv.* **2018**, *4*, 2720.

[4] E. Matabaro, H. Kaspar, P. Dahlin, D. L. V Bader, C. E. Murar, F. Staubli, C. M. Field, J. W. Bode, M. Künzler, *Sci. Rep.* **2021**, *11*, 3541.

[5] H. Luo, S.-Y. Hong, R. M. Sgambelluri, E. Angelos, X. Li, J. D. Walton, *Chem. Biol.* **2014**, *21*, 1610–1617.
