# Supplementary material for: Macrocyclization of backbone N-methylated peptides by a prolyl oligopeptidase with a distinctive substrate recognition mechanism
Source: Chem Sci. 2025 Jul 2;16(31):14196–206. doi: 10.1039/d5sc03723a (PMC12235650; doi:10.1039/d5sc03723a)
Supplement: SC-016-D5SC03723A-s002 [file SC-016-D5SC03723A-s002.pdf]

Supplementary Information (SI) for Chemical Science.
This journal is © The Royal Society of Chemistry 2025

Contents

Materials and methods 2
Cells, antibodies, and reagents 2
Plasmid construction 2
Production of recombinant OphP in *P. pastoris* 2
Protein expression in *E. coli* 3
Peptide production and purification 3
Protein sequences 4
Enzyme activity assays 5
LC-ESI-MS/MS analysis 7
ESI-TOF MS and intact mass measurement 7
ITC studies of OphP with peptides 7
Crystallization, data collection, reduction, and refinement.....................................8
Table S1. List of oligonucleotides 9
Table S2. List of plasmids...9
Table S3. Data collection, phasing and refinement statistics for all the structures.............10
References 11

Materials and methods

Cells, antibodies, and reagents

ProAlanase (mass spec grade) was ordered from Promega. Expression plasmids for recombinant GmPOPB (plasmid PMA 1237: pJ414-GmPOPB) and TEV protease (pRK793_MBP-TEV site-His6-TEV-C4) were previously described [1] and provided by Alvar Gossert (ETH Zürich, Switzerland), respectively. Phusion high-fidelity DNA polymerase was ordered from ThermoFisher Scientific (USA). Peptides AMA1, PHA1, SFTI-1A, SFTI-1B, SFTI-1C, SFTI-1E, SFTI-1F, SFTI-1G, SFTI-1H, SFTI-1I, SFTI-1K and SFTI-1M were chemically synthesized by GenScript Biotech (USA). SFTI-1D, SFTI-1J, SFTI-1L, and the clasp and follower peptides for the ITC experiments were chemically synthesized by Biosynth AG (Switzerland). The chromogenic substrate Gly-Pro-pNA and the POP inhibitor Z-Pro-prolinal (ZPP) were ordered from Sigma-Aldrich. Follower and leader (clasp domain) peptides were synthesized by Genscript.

Plasmid construction

The plasmids expressing OphP or LedP were constructed from pPIC3.5K-strepII-SUMO*-TEVcs-OphP (PMA1382) and pPIC3.5K-StrepII-SUMO*-TEVcs-LedP (PMA1379) that were previously used in omphalotin A production in *P. pastoris* [2]. The His_8-tag was fused to SUMO* by PCR amplification using PMA1379 as a template and His-SUMOSTAR-FW and His-SUMOSTAR-RV as primers. The obtained fragment was cloned into the *Spe*I and *BamH*I restriction sites of plasmids PMA1382 and PMA1379 to generate pPIC3.5K-His_8-SUMO*-TEVcs-OphP (PMA1596) and pPIC3.5K-His_8-SUMO*-TEVcs-LedP (PMA1595), respectively. The plasmid coding for His_8-SUMO*-TEVcs-OphP(S580A) was constructed by exchanging the *Not*I-*Spe*I restriction fragment of PMA1596 by the respective fragment of PMA1374 (pCDFDuet-sMBP-OphP(S580A)). For most of the His_8-OphMA-TEVcs constructs, one common primer OphMA-FW was used. For plasmids expressing OphMA-TEVcs-ΔVIG, OphMA-TEVcs-DbiCterm and OphMA-TEVcs-LedCterm, primers OphMA-ΔVIG-RV, OphMA-DbiCterm-RV, OphMA-LedCterm-RV were used together with OphMA-FW on PMA1488, respectively. The obtained PCR fragments were digested with *Not*I and *Nde*I and inserted in the respective sites on the pET24b vector. For the amplification of the coding region of His_8-OphMA-TEVcs-24mer, primer OphMA-24mer-RV was used on PMA1004. The PCR fragment was inserted into *Nde*I and *Nco*I of PMA1488. In all cases, the plasmids were amplified in *E. coli* DH5α. Plasmid sequences were confirmed by Sanger sequencing. Plasmids encoding His_8-SUMO*-TEVcs-OphP(I606A), His_8-SUMO*-TEVcs-OphP(W721A), His_8-OphMA-TEVcs-ΔC6(G408A), His_8-OphMA-TEVcs-ΔC6(G408V) and His_8-OphMA-TEVcs-ΔC6(G408L) were created using published protocols [3]. Plasmids PMA1488 (His_8-OphMA-TEVcs-21mer) and PMA1304 (His_8-OphMA-TEVcs-15mer) were part of our plasmid library. They were used to generate Oph-3 and -5 peptides, respectively. PMA1304 and PMA1488 were derived from plasmid PMA1004 (pET24-His_8-OphMA-cDNA) described earlier [4]. Used primers and plasmids are listed in Supplementary File 1-Tables S1 and S2, respectively.

Production of recombinant OphP in *P. pastoris*

*Pichia pastori*s GS115 cells were transformed by electroporation using the plasmids pPIC3.5K-His_8-SUMO*-TEVcs-OphP or pPIC3.5K-His_8-SUMO*-TEVcs-LedP, linearized by *Bsp*EI for the integration into the *HIS4* locus. Transformants were selected first on a standard SD-HIS minimal medium and then on 250 µg/ml geneticin for high-copy number screening. To produce OphP, cells were cultured at 20 °C in a standard methanol-containing complex medium (BMMY) for three days following the recommendations for *Pichia* protein expression[5]. For harvesting, cells were separated from the culture medium by centrifugation,

and cell pellets were washed with cold lysing buffer (2xPBS) or double-distilled water (dd H_2O) and centrifuged again at 8000 × *g* for 10 min at 4 °C. Cell pellets were resuspended in cell lysis buffer [2xPBS, 10% glycerol, 1 mM DTT, 20 mM imidazole, and protease inhibitor cocktail (PIC) (Roche)]. Cells lysis was performed on a planetary mill (Pulverisette 7) as previously described [5]. Cell debris were removed by spinning down the cell lysate at 16, 000 *x g* for 30 min at 4 °C. The cell lysate supernatant was collected and subjected to purification via a Ni^{2+}-based immobilized metal affinity chromatography (Ni-IMAC) system using the washing buffer (50 mM HEPES pH 8.0, 10% glycerol and 20 mM imidazole), and elution buffer (50mM HEPES pH 8.0, 10% glycerol and 400 mM imidazole). The purified proteins were stored in storage buffer (50 mM HEPES pH 8.0 + 10% glycerol + 5 mM DTT) at -80 °C. The His_8SUMO* tag was cleaved off by incubation with TEV protease (1:10, w/w) in assay buffer [300 mM NaCl, 50 mM TRIS pH 8.0, 1mM DTT and 10% Glycerol] overnight, at 25 °C. The reaction mixture was reloaded on a Ni-IMAC system to remove the His_8-SUMO* tag. The fractions containing OphP were collected and concentrated using an Amicon filter column (50 kDa cut-off). OphP was further polished across an Äkta FPLC system for size exclusion chromatography (SEC) equipped with a Superdex 200 Increase column. For the this final purification step, HEPES buffer [50 mM HEPES pH 8, 1 mM DTT, 10% glycerol (v/v)] was used, as previously described [5].

Protein expression in *E. coli*

OphMA, OphMA-TEV, GmPOPB, and TEV protease were produced in *E. coli* BL21 as previously described [1, 5]. Briefly, chemically competent BL21 cells were transformed with respective expression plasmids and selected on appropriate antibiotics. A pre-culture was incubated overnight in 10 ml at 37 ºC, 180 rpm. 5 ml of the preculture was used to inoculate 1 L of terrific broth (TB) medium. The culture was maintained at 37ºC, 160 rpm until OD_{600} of 1.5 to 2.0. The culture was chilled on ice for 30 min before the addition of 200 µM isopropyl β-D-thiogalactopyranoside (IPTG) and maintained at 16 ºC, 160 rpm for 2 days (GmPOPB and TEV) and 3-5 days for OphMA and OphMA-TEV mutants. Cells were harvested by centrifugation at 8000 × *g* for 20min, at 4ºC. Cells pellets were resuspended in 100 mL ice-cold lysis buffer [300 mM NaCl, 50 mM Tris pH 8.0, 10% glycerol, 1 mM DTT, DNase I, 20 mM Imidazole and protease inhibitor cocktail (Roche)]. For OphMA and OphMA-TEV mutants, NaCl and Tris in the buffer above, were replaced by 50mM HEPES pH 8.0, 0.1% Triton x-100. Cells were lysed by a cold French press, two passages, and clearing of cell debris by centrifugation at 12 000 rpm, 30 min at 4 ºC. The cell lysate supernatant was subjected to protein purification either by Ni-IMAC systems (for TEV and GmPOPB) or by Ni-NTA beads (Macherey Nagel). Elution and wash buffers and the SEC method were the same as for OphP purification above. The protein concentration was determined by BCA assay (Thermo Scientific). To confirm the protein quality, 10 µg of each protein was loaded on a 12% SDS gel and run by electrophoresis and stained by Coomassie brilliant blue. The purified proteins were shock-frozen in liquid nitrogen and then stored at -80 ºC.

Peptide production and purification

To produce OphMA-C-terminal fragments, purified and TEV-cleavable OphMA proteins were mixed with TEV protease (1:10) to a concentration of 10 mg/ml in assay buffer [300 mM NaCl, 50 mM TRIS pH 8.0, 1 mM DTT and 10 % glycerol]. The reaction was mixed well by pipetting and kept overnight at 25 °C. The reaction mixture was loaded on a pre-equilibrated C8 or C18-SepPak cartridge following the user instruction and recommendations. The peptides were eluted in 1-3 ml 100% methanol depending on the concentration. 5 µl of the eluate was used for HPLC-MS/MS analysis before further purification on an Agilent 1260 infinity preparative HPLC harboring a C18 column (Luna 5 µm C18(2) 100Å, 250X10 mm. The mobile phase

consisted of a gradient between water (A) and acetonitrile (B), both supplemented with 0.1% formic acid. The flow rate was set at 1.2 ml/min, and one fraction each minute. The method started with a system equilibration for 5 min at 5% B, followed by a linear gradient up to 40 % B in 10 min, then increased to 98% B in 30 min. The column was finally washed with 98% B for 5 min before gradually dropping to 5% B in a 5 min span. Fractions corresponding to the expected products were collected, and 5 µl of each fraction are tested by HPLC-MS/MS for the presence of peptides. To determine the concentration, samples were analysed by an Agilent 1100 series UV-HPLC calibrated to the bovine albumin serum (BSA) as an internal standard, at $\lambda = 210$ nm. To produce Oph-1, recombinant His$_8$-OphMA lacking a TEV protease cleavage site was digested with trypsin. For this purpose, 100 µg of the purified protein was incubated with 1.25 mg trypsin in 25 µl HEPES buffer pH 8.0, 37 °C, 650 rpm, overnight. 3 µl of the reaction was used for MS analysis. To purify the peptide, C18 cartridges (Sep-Pak C18 1cc (100mg), Waters) were used. The cartridge was first flushed with 8 ml 100% methanol and then equilibrated with 8 ml water. The trypsin reaction mixture was then loaded on the cartridge, followed by 3 washes with 1 mL water supplemented with 1% methanol. The peptides were eluted in 1 mL 100% methanol. The sample was concentrated by evaporating the solvent using a speed vac, and the resulting peptide pellets were resuspended in the assay buffer.

All our attempts to produce and purify non-methylated peptides by proteolytic cleavage of either the catalytically inactive R72A variant of OphMA or maltose-binding protein fusions of OphMA C-terminal fragments, failed.

Protein sequences (the portion used after TEV protease cleavage site (cs) is underlined, the TEV protease cleavage site is in bold)

His$_8$OphMA-TEVcs(Oph-3)
MEHHHHHHHHTSTQTKAGSLTIVGTGIESIGQMTLQALSYIEAAAKVFYCVIDPATE
AFILTKNKNCVDLYQYYDNGKSRLNTYTQMSELMVREVRKGLDVVGVFYGHPGVF
VNPSHRALAIAKSEGYRARMLPGVSAEDCLFADLCIDPSNPGCLTYEASDFLIRDRPV
SIHSHLVLFQVGCVGIADFNFTGFDNNKFGVLVDRLEQEYGAEHPVVHYIAAMMPH
QDPVTDKYTVAQLREPEIAKRVGGVSTFYIPPKARKASNLDIIRRLELLPAGQVPDKK
ARIYPANQWEPDVPEVEPYRPSDQAAIAQLADHAPPEQYQPLATSKAMSDVMTKLA
LDPKALADYKADHRAFAQSVPDLTPQERAALELGDSWAIRCAMKNMPSSLLDAAR
ESG**ENLYFQG**FPWVIVVGVIGVIGSVMSTE

His$_8$OphMA-TEVcs-ΔVIG(Oph-4)
MEHHHHHHHHTSTQTKAGSLTIVGTGIESIGQMTLQALSYIEAAAKVFYCVIDPATE
AFILTKNKNCVDLYQYYDNGKSRLNTYTQMSELMVREVRKGLDVVGVFYGHPGVF
VNPSHRALAIAKSEGYRARMLPGVSAEDCLFADLCIDPSNPGCLTYEASDFLIRDRPV
SIHSHLVLFQVGCVGIADFNFTGFDNNKFGVLVDRLEQEYGAEHPVVHYIAAMMPH
QDPVTDKYTVAQLREPEIAKRVGGVSTFYIPPKARKASNLDIIRRLELLPAGQVPDKK
ARIYPANQWEPDVPEVEPYRPSDQAAIAQLADHAPPEQYQPLATSKAMSDVMTKLA
LDPKALADYKADHRAFAQSVPDLTPQERAALELGDSWAIRCAMKNMPSSLLDAAR
ESG**ENLYFQG**FPWVIVVGVIGSVMSTE

His$_8$OphMA-TEVcs-ΔC6(Oph-5)
MEHHHHHHHHTSTQTKAGSLTIVGTGIESIGQMTLQALSYIEAAAKVFYCVIDPATE
AFILTKNKNCVDLYQYYDNGKSRLNTYTQMSELMVREVRKGLDVVGVFYGHPGVF
VNPSHRALAIAKSEGYRARMLPGVSAEDCLFADLCIDPSNPGCLTYEASDFLIRDRPV
SIHSHLVLFQVGCVGIADFNFTGFDNNKFGVLVDRLEQEYGAEHPVVHYIAAMMPH

QDPVTDKYTVAQLREPEIAKRVGGVSTFYIPPKARKASNLDIIRRLELLPAGQVPDKK
ARIYPANQWEPDVPEVEPYRPSDQAAIAQLADHAPPEQYQPLATSKAMSDVMTKLA
LDPKALADYKADHRAFAQSVPDLTPQERAALELGDSWAIRCAMKNMPSSLLDAAR
ESG**ENLYFQG**FPWVIVVGVIGVIG

His$_8$OphMA-TEVcs-24mer.
MEHHHHHHHHTSTQTKAGSLTIVGTGIESIGQMTLQALSYIEAAAKVFYCVIDPATE
AFILTKNKNCVDLYQYYDNGKSRLNTYTQMSELMVREVRKGLDVVGVFYGHPGVF
VNPSHRALAIAKSEGYRARMLPGVSAEDCLFADLCIDPSNPGCLTYEASDFLIRDRPV
SIHSHLVLFQVGCVGIADFNFTGFDNNKFGVLVDRLEQEYGAEHPVVHYIAAMMPH
QDPVTDKYTVAQLREPEIAKRVGGVSTFYIPPKARKASNLDIIRRLELLPAGQVPDKK
ARIYPANQWEPDVPEVEPYRPSDQAAIAQLADHAPPEQYQPLATSKAMSDVMTKLA
LDPKALADYKADHRAFAQSVPDLTPQERAALELGDSWAIRCAMKNMPSSLLDAA**E**
NLYFQGSQNGFPWVIVVGVIGVIGSVMSTE

His8-SUMO*-TEVcs-OphP
MGHHHHHHHHGGSDSEVNQEAKPEVKPEVKPETHINLKVSDGSSEIFFKIKKTTPLR
RLMEAFAKRQGKEMDSLTFLYDGIEIQADQTPEDLDMEDNDIIEAHREQIGG**ENLYF**
QGTSMSFPGWGPYPPVERDETSAITYSSKLHGSVTVRDPYSQLEVPFEDSEETKAFV
HSQRKFARTYLDENPDREAWLETLKKSWNYRRFSALKPESDGHYYFEYNDGLQSQL
SLYRVRMGEEDTVLTESGPGGELFFNPNLLSLDGNAALTGFVMSPCGNYWAYGVSE
HGSDWMSIYVRKTSSPHLPSQERGKDPGRMNDKIRHVRFFIVSWTSDSKGFFYSRYP
PEDDEGKGNAPAMNCMVYYHRIGEDQESDVLVHEDPEHPFWISSVQLTPSGRYILFA
ASRDASHTQLVKIADLHENDIGTNMKWKNLHDPWEARFTIVGDEGSKIYFMTNLKA
KNYKVATFDANHPDEGLTTLIAEDPNAFLVSASIHAQDKLLLVYLRNASHEIHIRDLT
TGKPLGRIFEDLLGQFMVSGRRQDNDIFVLFSSFLSPGTVYRYTFGEEKGYRSLFRAIS
IPGLNLDDFMTESVFYPSKDGTSVHMFITRPKDVLLDGTSPVLQYGYGGFSLAMLPT
FSLSTLLFCKIYRAIYAIPNIRGGSEYGESWHREGMLDKKQNVFDDFNAATEWLIAN
KYASKDRIAIRGGSNGGVLTTACANQAPGLYRCVITIEGIIDMLRFPKFTFGASWRSE
YGDPEDPEDFDFIFKYSPYHNIPPPGDTVMPAMLFFTAAYDDRVSPLHTFKHVAALQ
HNFPKGPNPCLMRIDLNSGHFAGKSTQEMLEETADEYSFIGKSMGLTMQTQGSVDSS
RWSCVTV

Enzyme activity assays
For the chromogenic substrate and the POP inhibitor, stocks of 50 mM and 10 mM, respectively, were prepared in methanol. Before the reaction, the substrate was solubilized at 40 °C and then the required amounts were mixed with the standard assay buffer [50 mM HEPES pH 6.0 + 10 mM DTT]. The enzyme was added last, to make the total reaction volume 50 µL. The reaction mixture was kept at 30°C while shaking at 600 rpm. The reaction was quenched by the addition of 50 µl methanol. The enzymatic activity was monitored by absorbance measurement at 410 nm (for *p*-nitroanilide) on an Infinite 200Pro M Plex spectrophotometer (Tecan). Continuous reaction monitoring was ensured by incubating the 96-well plates at 30°C, with absorbance measurements each 30 min.

For peptide substrates, reactions were also carried out in 50 µl with a standard buffer [50 mM HEPES pH 7.0, 10 mM DTT], at 30°C, 600 rpm unless stated otherwise. The peptide was first mixed with the buffer before adding the enzymes. Both substrate and enzyme concentrations varied up to 100 µM. The reaction was stopped by the addition of 50 µl methanol, then spun down at the highest speed (20,000 *x g*), 4ºC. For HPLC-MS/MS analysis, 5 µl supernatant was used. For the synthetic peptides, PHA1 and AMA1 were stored in 1 mg/ml. For the reaction,

20 μM substrate was used with 1 μM of protease. For ProAlanase, a 1:50 enzyme/substrate (w/w) was used in a 25 μl reaction volume, in either 50 mM HEPES (pH 6.0) or 50 mM sodium acetate (pH 4.0). These reactions were carried out at 37°C for 4 h and stopped by adding 25 μl methanol. After centrifugation at the highest speed at 4 °C, 5 μl supernatant was used for HPLC-MS/MS analysis.

For the time course experiment with OphP and Oph-5^{high} (**Figure 3 b,c,d and S12 c, d**), the reactions were performed in buffer containing 20 mM HEPES (pH 7.0), 100 mM KOAc, 2mM Mg(OAc)2, 10% glycerol and 10 mM DTT at a concentration of 100 μM total Oph-5^{high} (90 μM 7-fold methylation, 9 μM 6-fold methylation) and 10 μM OphP. The reactions were performed in 70 μl reaction volume at 30°C and at each time point, 5 μl were sampled and added to 20 μl of MeOH to stop the reaction. The samples were centrifuged for 5 minutes at 15000 rcf and 10 μl were injected for HPLC-MS/MS analysis.

For the experiment of OphP with Oph-5^{high} (mixture of 7-fold and 6-fold methylations) and protease inhibitors (**Figure S9 b,c**), the reactions were performed in buffer containing 20 mM HEPES (pH 7.0), 100 mM KOAc, 2mM Mg(OAc)2, 10% glycerol and 10 mM DTT at a concentration of 100 μM total Oph-5^{high} (90 μM 7-fold methylation, 9 μM 6-fold methylation) and 10 μM OphP. DMSO stocks of the different inhibitors were diluted in the HEPES buffer and added to the reaction to the indicated final concentration. The highest used DMSO concentration was used in a control reaction without inhibitors The reactions were performed in 5 μl reaction volume at 30°C and 20 μl of MeOH were added after 30 minutes to stop the reaction. The samples were centrifuged for 5 minutes at 15000 xg and 10 μl were injected for HPLC-MS/MS analysis.
For the experiment testing for preferred production of 7-fold over 6-fold methylated species at equimolar substrate concentration (**Figure S12 b**), the reactions were performed with two different Oph-5 substrate concentrations of 100 μM and 1000 μM total Oph-5^{high} substrate.

The reactions of GmPOPB and OphP with the SFTI-1 variants, AMA1, PHA1 and Oph-5^{high} peptide substrates (**Figure 4 and S13)** were performed in buffer containing 20 mM HEPES (pH 7.0), 100 mM KOAc, 2mM Mg(OAc)2, 10% glycerol and 10 mM DTT at a concentration of 100 μM peptide substrate and 10 μM OphP or GmPOPB. The reactions were performed as triplicates in 10 μl reaction volume and incubated at 30°C for 60 minutes before addition of 20 μl of MeOH to stop the reaction. 10 μl were injected for HPLC-MS/MS analysis.

For assays of OphP variants with Oph-5^{high}, the reactions were performed in buffer containing 50 mM HEPES (pH 7.0), 100 mM NaCl, and 5 mM DTT at a concentration of 200 μM Oph-5 and 20 μM OphP. The reaction mixtures in Eppendorf tubes were incubated at 25 °C and 1000 rpm using a ThermoMixer C for 18 hours. Reactions were quenched by adding an equal amount of methanol and protein precipitation was removed by centrifugation. The supernatant was analysed using Waters Acquity H-Class plus UPLC equipped with SQ, PDA and ELS detectors. Buffer A (water with 0.1% FA) and buffer B (acetonitrile with 0.1% FA) are used as mobile phase. Typically, 10 μL of supernatant was loaded and separated on a ACQUITY UPLC BEH C18 column (1.7 μM, 2.1 x 100 mm) at a flow rate of 0.6 ml/min with following gradient: 0-0.25 min for 5% buffer B, 0.25-0.4 min from 5% to 40% buffer B, 0.4-3.6 min from 40% to 55% buffer B, 3.6-3.7 min from 55% to 60% buffer B, 3.7-6.0 min from 60% to 70% buffer B, 6.0-6.15 min from 70% to 95% buffer B, 6.15-7.6 min for 95% buffer B.

LC-ESI-MS/MS analysis

Peptide analysis was performed by following previous protocols[5] for HPLC-MS/MS analysis of borosins. Briefly, data were generated from Dionex Ultimate 3000 UHPLC HPLC coupled to an MS/MS system (Thermo Scientific Q Exactive classic). A list of expected masses (inclusion list) was always included in the HPLC-MS/MS method. The peptide was applied on a C18 column (Phenomenex Kinetex ®µm XB-C18 100 Å (150 x 2.1 mm)) pre-heated at 50 °C. For UV-vis spectra, the signals were recorded by a diode array detector (DAD) set at 210, 230, 250, and 280 nm. Water (solvent A) and acetonitrile (solvent B) both containing 0.1% FA served as a mobile phase, with a 0.5 ml/min flow rate. The HPLC method started by equilibrating the system for 2 min at 5% B, followed by a linear gradient up to 70 % B in 13 min, and then increased to 100% B in 3 min. The column was rinsed first with 100% B for 1 min and then with 5% B for 5 min. The MS/MS system used the heated electrospray ionization in a positive ion mode. The full MS was set as follows: an automatic gain control (AGC) target of 70,000 [AGC target 1e6, maximum ion trap (IT), 120 ms, and scan range of 200-2500m/z]. Data-dependent (dd) MS/MS was performed at a resolution of 17,500 [AGC target at 1e5, maximum IT 120 ms, and isolation window of 3.0 m/z]. The normalized collision energy (NCE) was set at 30% for macrocycles, and 18% or stepped NCE (16, 18, 22%) for linear peptides.

ESI-TOF MS and intact mass measurement

Intact protein mass analysis was performed using a Waters Xevo G2-QS QToF MS coupled to an ACQUITY UPLC I-Class LC system (Waters Corporation). The spectrometer was auto-calibrated throughout the experiment using an internal lock-spray mass calibrant (at 556.2771 m/z of leucine enkephalin, 1 s scan every 120 s interval). The data was acquired in Continuum format, in a 300 – 2500 m/z spectral window with a scan time of 1 s and an interscan time of 0.014 s. The mass spectrometer and ESI ionization source were operated under the following parameters: capillary voltage at 3 kV, cone voltage at 20 V, source offset voltage at 40 V, source temperature at 100 °C, desolvation temperature at 400 °C and desolvation gas (nitrogen) flow at 650 L/h, cone gas flow at 30 L/h. In a typical analysis, 1 µL (30 ng) of the protein sample was injected and desalted on a ProSwift™ RP-1S HPLC (250 x 4.6 mm x 5 µm, Thermo Scientific™) column maintained at 60 °C. The elution was performed using solvent A (water with 0.1% formic acid) and solvent B (acetonitrile with 0.1% formic acid) with a gradient elution using isocratic 95% solvent A (0 - 1 min), linear gradient to 95% solvent B (1.1 – 7 min), and isocratic 95% solvent A (7.1 – 10 min) at a flowrate of 0.2 ml/min. Combined mass spectra from the total chromatographic protein peak were used for intact mass reconstruction using MassLynx (Waters) Max(imum) Ent(ropy) 1 deconvolution algorithm (Resolution: 0.5 Da/channel, Width at half-height: ion series/protein-dependent, Minimum intensity ratios: 33% Left and Right). Spectra were deconvoluted between 40000 and 55000 for OphMA.

ITC studies of OphP with peptides

Isothermal titration calorimetry (ITC) experiments were performed using a MicroCal PEAQ-ITC (Malvern). Leader peptides and follower peptides (SVMSTE and VIGSVMSTE) were dissolved in DMSO at sufficient concentration as a stock. A typical ITC experiment was performed by repeated injections 2 µL of peptide solution eighteen times into a cell solution containing 0.4 ml of S580A. The measurements were done at 25 °C at a stirring rate of 750 rpm with a reference power (DP) of 5 mcal/s or 10 mcal/s. Control titrations were performed similarly except for the absence of protein in the cell solution. Data processing and fitting were performed using MicroCal PEAQ-ITC analysis software (Malvern).

Crystallization, data collection, reduction, and refinement

Freshly purified OphP and S580A proteins were screened for apo crystals using the sitting drop method using commercial screening kits from Hampton Research or Molecular Dimensions. OphP and S580A were eventually crystallized at 40-50 mg/ml by mixing 0.3 μL of proteins with 0.3 μL of the reservoir (0.2 M sodium acetate, 0.1 M Bis-Tris propane (pH 6.0-6.5) and 28% PEG 3350). Crystals appeared within eight days and grew to full size within three weeks at room temperature. Crystals were fished, transferred to cryo-protectant (reservoir solution supplemented with 40% PEG 3350) and flash-frozen in liquid nitrogen. To obtain the OphP-Z-Pro-prolinal (ZPP) complex, OphP crystals with 5 mM ZPP overnight at room temperature. Crystals employed the same cryoprotectant as the apo but supplemented with 1 mM ZPP. Crystals were flash-frozen in liquid nitrogen. For the S580A:Oph-15mer complex, apo S580A crystals were soaked with 1 mM purified Oph-15mer overnight at room temperature. Due to the poor solubility of Oph-18mer^{NL}, apo OphP(S580A) crystals were soaked with a cloudy reservoir solution containing 18mer overnight to obtain the OphP(S580A)-18mer^{CF} complex. Both complexes were cryoprotected in the same manner the apo crystals but with cryoprotectant supplemented with ligand before being flash flash-frozen in liquid nitrogen

All X-ray diffraction data were recorded at Diamond Light Source using automated beamlines (I03, I04, I04-1, and I24). The diffraction images were reduced, integrated, and scaled using xia2 [6], DIALS [7] pipeline without manual intervention. The 1.9 Å apo structure of S580A was determined by molecular replacement using a Phaser [8] with the GmPOPB structure (PDB entry 5N4C) as the search model. Automatic rebuilding of this initial molecular replaced structure was performed using Buccanner which also with changed the sequence from GmPOPB to OphP. This much improved structure was further refined cycling between Refmac5 [9] and manual intervention with Coot[10]. The structure was finalized using PDB-redo [11]. Complex structures were solved by molecular replacement using the finalized apo S580A structure as the search model and refined using Refmac5 [9]. More than 50 crystals dataset were collected for each complex, the residual electron density after initial refinement was assessed to determine whether a ligand was present and the interpretability of density. Based on judgement of the quality of the density for the ligand and the resolution of the data, a data set for the ZPP (2.0 Å), 15mer (2.47 Å) and 18 mer (2.0 Å) was selected. Simulated omit maps for the ligands were calculated using Phenix to guide the final positioning of the ligand for each complex [12]. Complex structures were finalized with the assistance of PDB-redo [11]. All crystallographic figures were generated using Pymol (Schrödinger LLC), CCP4mg [13] or Chimera [14]. Structure-based sequence alignment was created using MUSCLE [15] and ESPript 3.0 [16]. Full crystallographic details are given in Table 3.

Table S1. List of oligonucleotides

His-SUMOSTAR-FW	GAAGGATCCGAAACTATGGGTCATCATCATCATCATCATCATCATGGTGGTTCT GACTCCGAGGTC
SUMOSTAR-RV	AGACATACTAGTACCTTGGAAGTACAAGTTTTC
OphMA-NotI-RV	GAGTGCGGCCGCTTATTCCGTGCTCATGACTGATC
OphP-FW	AAGGTACTAGTATGTCGTTTCCAGGATGGGGACCAT
OphMA-FW	ATATACATATGGAGCATCATCATCATCATCATCATCATACTTCCACTC
OphMA-DbiCterm-RV	GCTCGAATTCTTACGCGCTGCTAACCACGCTTCCGATGACCCCAACGATACCCG TGACGATGACCCATGGGAAACC
OphMA-LedCterm-RV	CTCGAATTCTTACGCGCTGCTAACCACGCTACCAACCACGCCAACCACACCAAC CACAATGATCCATGGGAAACCTTGGAAGTAC
OphMA-ΔVIG-RV	GTAGCAGCAACCACAATAGCCAAGTCAGTACTCGTGCCTTATTCGCCGGCGGTC
OphMA-24mer-RV	TGACCCATGGGAAACCGTTTTGGGATTGGAAGTACAAGTTTTCACGAGCAGCGT CCAAGAGCGACG
OphP-I606A-FW	GAGGGTGCAATTGATATGCTTAGATTTCCTAAGTTTACATTTG
OphP-I606A-RV	CATATCAATTGCACCCTCAATTGTGATCACACAACGGTAGAG
OphP-W621A-FW	GCTAGCGCACGTTCTGAATATGGCGATCCTGAGGATCCTG
OphP-W621A-RV	TCAGAACGTGCGCTAGCGCCAAATGTAAACTTAGGAAATCTA
OphMAΔC6-G408A-FW	GTCGTTGGTGTTATCGCTGTCATCGGATAAGCGGCCGC
OphMAΔC6-G408A-RV	AGCGATAACACCAACGACGATGACCCATGGGAAACCGTTT
OphMAΔC6-G408V-FW	GTCGTTGGTGTTATCGTCGTCATCGGATAAGCGGCCGC
OphMAΔC6-G408V-RV	GACGATAACACCAACGACGATGACCCATGGGAAACCGTTT
OphMAΔC6-G408L-FW	GTCGTTGGTGTTATCCTCGTCATCGGATAAGCGGCCGC
OphMAΔC6-G408L-RV	GAGGATAACACCAACGACGATGACCCATGGGAAACCGTTT

Table S2. List of plasmids

Name	#	Reference/Source
pPIC3.5K-strepII-SUMO*-TEVcs-OphP	PMA1382	[2]
pPIC3.5K-strepII-SUMO*-TEVcs-OphP	PMA1379	[2]
pCDFDuet-sMBP-OphP(S580A)	PMA1374	E. Matabaro, unpublished
pPIC3.5K-His_8-SUMO*-TEVcs-OphP	PMA1596	This study
pPIC3.5K-His_8-SUMO*-TEVcs-LedP	PMA1595	This study
pPIC3.5K-His_8-SUMO*-TEVcs-OphP(S580A)	PMA1597	This study
pPIC3.5K-His_8-SUMO*-TEVcs-OphP(I606A)		This study
pPIC3.5K-His_8-SUMO*-TEVcs-OphP(W721A)		This study
pET24-His_8-OphMA(cDNA)	PMA1004	[4]
pET24-His_8-OphMA-TEVcs-ΔC6(15mer*)	PMA1304	[4]
pET24-His_8-OphMA-TEVcs-ΔVIG(18mer*)	PMA1637	This study
pET24-His_8-OphMA-TEVcs(21mer*)	PMA1488	[4]
pET24-His_8-OphMA-TEVcs-24mer*	PMA1640	This study
pET24-His_8-OphMA-TEVcs-ΔC6(G408A)		This study
pET24-His_8-OphMA-TEVcs-ΔC6(G408V)		This study
pET24-His_8-OphMA-TEVcs-ΔC6(G408L)		This study

*Size of peptide released by TEV protease

Table S3. Data collection, phasing and refinement statistics for all the structures.

	Apo S580A	OPhP-ZPP	S580A:Oph-5	S580A:Oph-6
PDB Entry	**7ZB2**	**7ZAZ**	**7ZB0**	**7ZB1**
**Data collection**				
Space group	P 1	P 1	P 1	P 1
Cell dimensions				
a, *b*, *c* (Å)	69.86, 113.43, 186.32	69.68, 102.65, 110.27	70.22,106.18, 114.79	70.01, 104.61, 110.65
α, β, γ (°)	83.97, 82.09, 76.93	116.23, 101.09, 92.18	113.04, 101.67, 93.24	115.81, 98.87, 93.79
Wavelength (Å)	0.9795	0.9763	0.9763	0.9762
Resolution (Å)	66.00-1.94 (1.97-1.94)	67.71-2.00 (2.03 -2.00)	64.35-2.47 (2.51-2.47)	55.84 -2.00 (2.03-2.00)
R_{merge}	0.075 (0.993)	0.115 (1.099)	0.120 (1.375)	0.090 (1.234)
$I / \sigma I$	7.3 (0.9)	14.3 (1.2)	13.0 (0.9)	15.0 (1.0)
Completeness (%)	97.6 (96.6)	97.6 (92.3)	98.6(97.96)	98.0 (96.9)
Redundancy	2.5 (2.4)	3.5 (3.4)	3.6 (3.6)	3.3 (3.5)
No. Unique reflections	999365 (397483)	611334 (175661)	370936 (104483)	598522 (182773)
$CC_{1/2}$	0.996 (0.315)	0.990 (0.580)	0.993 (0.530)	0.996 (0.545)
**Refinement**				
Resolution (Å)	66.00-1.94 (1.97-1.94)	67.71-2.0 (2.03-2.0)	64.35-2.47 (2.51-2.47)	55.84-2.0 (2.03 -2.0)
R_{work} / R_{free}	0.214/0.241	0.210/0.245	0.227/0.263	0.201/0.235
No. atoms				
Protein	46235	22799	22933	23204
Ligand/ion	89	257	276	251
Water	898	1328	53	682
B-factors (Å^2)				
Protein	46	37	77	47
Ligand/ion	61	56	103	77
Water	37	37	62	40
R.m.s deviations				
Bond lengths (Å)	0.007	0.006	0.007	0.006
Bond angles (°)	1.35	1.32	1.42	1.33
Ramachandran				
Allowed (%)	99.7	99.7	99.8	99.8
Outliers (%)	0.3	0.3	0.2	0.2

*Values in parentheses are for highest-resolution shell.

References

[1] C. M. Czekster, H. Ludewig, S. A. McMahon, J. H. Naismith, *Nature communications* **2017**, *8*, 1-10.

[2] E. Matabaro, H. Kaspar, P. Dahlin, D. L. Bader, C. E. Murar, F. Staubli, C. M. Field, J. W. Bode, M. Künzler, *Scientific reports* **2021**, *11*, 1-12.

[3] H. Liu, J. H. Naismith, *BMC biotechnology* **2008**, *8*, 1-10.

[4] N. S. van der Velden, N. Kälin, M. J. Helf, J. Piel, M. F. Freeman, M. Künzler, *Nature Chemical Biology* **2017**, *13*, 833-835.

[5] E. Matabaro, H. Song, C. Chepkirui, H. Kaspar, L. Witte, J. H. Naismith, M. F. Freeman, M. Künzler, in *Methods in Enzymology, Vol. 656*, Elsevier, **2021**, pp. 429-458.

[6] G. Winter, *Journal of applied crystallography* **2010**, *43*, 186-190.

[7] G. Winter, D. G. Waterman, J. M. Parkhurst, A. S. Brewster, R. J. Gildea, M. Gerstel, L. Fuentes-Montero, M. Vollmar, T. Michels-Clark, I. D. Young, *Acta Crystallographica Section D* **2018**, *74*, 85-97.

[8] A. J. McCoy, R. W. Grosse-Kunstleve, P. D. Adams, M. D. Winn, L. C. Storoni, R. J. Read, *Journal of applied crystallography* **2007**, *40*, 658-674.

[9] G. N. Murshudov, A. A. Vagin, E. J. Dodson, *Acta Crystallographica Section D: Biological Crystallography* **1997**, *53*, 240-255.

[10] P. Emsley, B. Lohkamp, W. G. Scott, K. Cowtan, *Acta Crystallographica Section D: Biological Crystallography* **2010**, *66*, 486-501.

[11] R. P. Joosten, K. Joosten, G. N. Murshudov, A. Perrakis, *Acta Crystallographica Section D: Biological Crystallography* **2012**, *68*, 484-496.

[12] D. Liebschner, P. V. Afonine, M. L. Baker, G. Bunkóczi, V. B. Chen, T. I. Croll, B. Hintze, L.-W. Hung, S. Jain, A. J. McCoy, *Acta Crystallographica Section D: Structural Biology* **2019**, *75*, 861-877.

[13] S. McNicholas, E. Potterton, K. Wilson, M. Noble, *Acta Crystallographica Section D: Biological Crystallography* **2011**, *67*, 386-394.

[14] E. F. Pettersen, T. D. Goddard, C. C. Huang, G. S. Couch, D. M. Greenblatt, E. C. Meng, T. E. Ferrin, *Journal of computational chemistry* **2004**, *25*, 1605-1612.

[15] R. C. Edgar, *Nucleic acids research* **2004**, *32*, 1792-1797.

[16] X. Robert, P. Gouet, *Nucleic acids research* **2014**, *42*, W320-W324.
